# Age-related susceptibility to insulin resistance arises from a combination of CPT1B decline and lipid overload

**DOI:** 10.1186/s12915-021-01082-5

**Published:** 2021-07-30

**Authors:** Marcel A. Vieira-Lara, Marleen B. Dommerholt, Wenxuan Zhang, Maaike Blankestijn, Justina C. Wolters, Fentaw Abegaz, Albert Gerding, Ydwine T. van der Veen, Rachel Thomas, Ronald P. van Os, Dirk-Jan Reijngoud, Johan W. Jonker, Janine K. Kruit, Barbara M. Bakker

**Affiliations:** 1 Laboratory of Pediatrics, Systems Medicine of Metabolism and Signaling, University Medical Center Groningen, University of Groningen, Postbus 196, 9700 AD Groningen, The Netherlands; 2 Department of Analytical Biochemistry, Groningen Research Institute of Pharmacy, University of Groningen, Groningen, The Netherlands; 3 Dutch Molecular Pathology Centre, Department of Pathobiology, Faculty of Veterinary Medicine, Utrecht University, Utrecht, The Netherlands; 4 Central Animal Facility, Mouse Clinic for Cancer and Aging, University Medical Center Groningen, University of Groningen, Groningen, The Netherlands

**Keywords:** Insulin resistance, Skeletal muscle, Mitochondrial β-oxidation, Ageing

## Abstract

**Background:**

The skeletal muscle plays a central role in glucose homeostasis through the uptake of glucose from the extracellular medium in response to insulin. A number of factors are known to disrupt the normal response to insulin leading to the emergence of insulin resistance (IR). Advanced age and a high-fat diet are factors that increase the susceptibility to IR, with lipid accumulation in the skeletal muscle being a key driver of this phenomenon. It is debated, however, whether lipid accumulation arises due to dietary lipid overload or from a decline of mitochondrial function. To gain insights into the interplay of diet and age in the flexibility of muscle lipid and glucose handling, we combined lipidomics, proteomics, mitochondrial function analysis and computational modelling to investigate young and aged mice on a low- or high-fat diet (HFD).

**Results:**

As expected, aged mice were more susceptible to IR when given a HFD than young mice. The HFD induced intramuscular lipid accumulation specifically in aged mice, including C18:0-containing ceramides and diacylglycerols. This was reflected by the mitochondrial β-oxidation capacity, which was upregulated by the HFD in young, but not in old mice. Conspicuously, most β-oxidation proteins were upregulated by the HFD in both groups, but carnitine palmitoyltransferase 1B (CPT1B) declined in aged animals. Computational modelling traced the flux control mostly to CPT1B, suggesting a CPT1B-driven loss of flexibility to the HFD with age. Finally, in old animals, glycolytic protein levels were reduced and less flexible to the diet.

**Conclusion:**

We conclude that intramuscular lipid accumulation and decreased insulin sensitivity are not due to age-related mitochondrial dysfunction or nutritional overload alone, but rather to their combined effects. Moreover, we identify CPT1B as a potential target to counteract age-dependent intramuscular lipid accumulation and thereby IR.

**Supplementary Information:**

The online version contains supplementary material available at 10.1186/s12915-021-01082-5.

## Background

The skeletal muscle is one of the principal tissues to increase postprandial glucose uptake in response to insulin. If the normal metabolic response to insulin is compromised, a condition known as insulin resistance (IR) arises [1]. Given its major role in maintaining whole-body glucose homeostasis, skeletal muscle IR precedes the onset of type 2 diabetes [2]. Lipid-induced IR is the most important form of IR in the skeletal muscle [1, 3, 4], for which ageing is a risk factor [5].

Different mechanisms have been proposed to explain how lipids could interfere with normal glucose uptake and utilization in the skeletal muscle. The first mechanism, initially described by Randle, is a fatty acid-glucose cycle in which fatty acids and glucose reciprocally inhibit each other’s oxidation by allosteric enzyme inhibition [6]. Since then, the focus has shifted to the insulin signalling cascade, which triggers the recruitment of the glucose transporter GLUT4 to the plasma membrane [1]. Diacylglycerols (DGs) and ceramides (Cers) are the two major acknowledged lipid classes to cause IR by inhibiting the insulin signalling cascade [1, 4, 7].

The accumulation of such lipid species and lipid-induced IR can be ascribed to an imbalance between fatty acid uptake and mitochondrial oxidation [7–9]. The current debate concerns the question of whether lipids accumulate due to mitochondrial dysfunction [9] or as a result of an overload by excess nutrients [10, 11]. On the one hand, the idea of an underlying mitochondrial defect is supported by reports that increasing the fatty acid β-oxidation capacity protects against IR [12, 13]. Moreover, increased age has been associated with a decrease in mitochondrial function across different species [14–16]. On the other hand, different studies have shown that inhibiting the first steps of β-oxidation can prevent the development of lipid-induced IR [10, 17, 18], which led to the idea that overloading the pathway causes a metabolic ‘gridlock’ [11]. In line with this, computer simulations suggested that the β-oxidation is intrinsically susceptible to such a gridlock by an accumulation of intermediate metabolites, leading to inhibition of downstream metabolism [19].

In this study, we address the interaction between mitochondrial dysfunction and nutritional overload in mice. A potential interaction between both mechanisms is supported by the finding that advanced age leads to a higher susceptibility to high-fat diet (HFD)-induced IR due to changes in fatty acid handling [20, 21]. We investigated by lipidomics profiling how advanced age exacerbates HFD-induced lipid accumulation in mouse quadriceps and its relation to peripheral insulin sensitivity. Subsequently, we used a systems biology approach to characterize mitochondrial lipid handling by high-resolution respirometry, targeted proteomics and computational modelling. We found that aged mice lose their mitochondrial flexibility in the quadriceps to respond to a HFD and identified a specific role for CPT1B.

## Results

### Ageing increases susceptibility to develop diet-induced insulin resistance

**Fig. 1:**
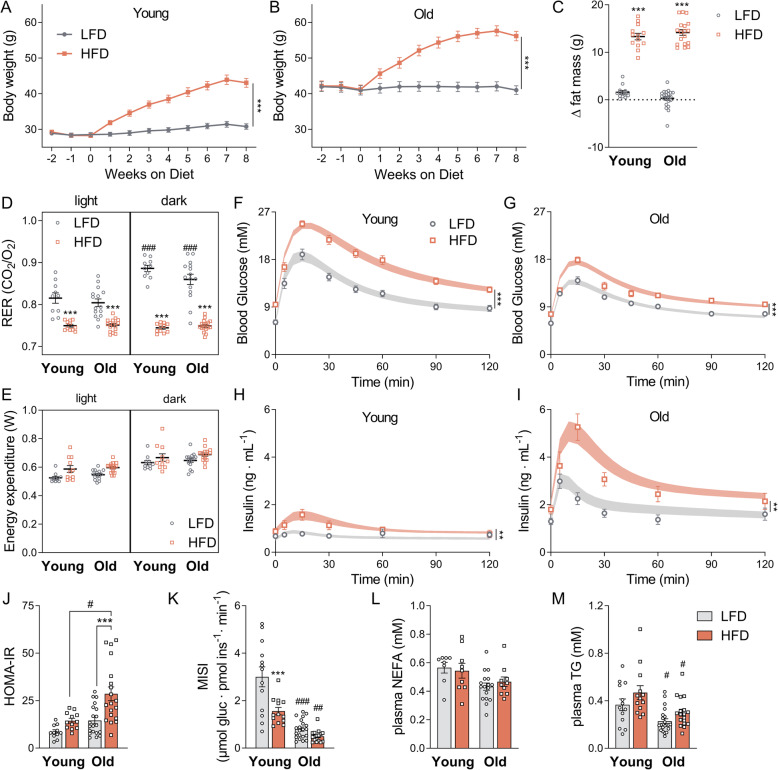
Aged mice are more susceptible to develop diet-induced insulin resistance. **a**, **b** Weight increase during 8 weeks in young and old mice, respectively, when submitted to either a LFD or HFD. **c** Increase in fat mass after 8 weeks on a HFD. **d** Average respiratory exchange ratios (RER) in both light/dark phases following 11 weeks on each diet. **e** Energy expenditure in both light/dark phases after 11 weeks on each diet. Data were corrected with ANCOVA with BW as a covariate for both light phases in old and aged mice (adjusted body weight = 45 g). Plasma glucose concentrations for young (**f**) and old (**g**) mice and plasma insulin levels for young (**h**) and old (**I**) mice are plotted for 120 min following an oral glucose tolerance test (OGTT) (1.5 g/kg BW glucose), performed after 9 weeks on either a LFD or a HFD. The points represent experimental data, and fitted curves are summarized in the shaded area. **j** HOMA-IR indexes adjusted to mice were calculated from the OGTT data as [fasting glucose in mM] × [insulin in mU/L]/14.1. **k** Muscle Insulin Sensitivity Index (MISI) calculated from fitted glucose and insulin curves, adapted from O’Donovan et. al. [22]. **l**, **m** Plasma non-esterified fatty acids (NEFAs) and triglycerides (TG). Data are shown as mean ± SEM. Statistical analysis was conducted for each group according to the “Materials and Methods” section, n = 10–13 (young groups) or 15–22 (old groups); ^###^*p* < 0.001 (old vs young), ^##^*p* < 0.01 (old vs young), ^#^*p* < 0.05 (old vs young), ***p < 0.001 (LF vs HF), **p < 0.01 (LF vs HF), *p < 0.05 (LF vs HF). Each pairwise comparison analysis means that differ by only one factor, meaning that age comparisons have matched diet and diet comparisons have matched age. Supporting data values can be found in Additional file 10.

Young (3 months) and old (18 months) C57BL/6J mice were given either a low- (LFD) or high-fat diet (HFD) for 12 weeks (Additional file 1: Fig. S1A). The diets consisted of 20% and 60% of kcal from fat, respectively (Additional file 2: Supp. Table 1). Despite old mice being already heavier than young mice, both age groups fed with a HFD gained more weight than their LFD counterparts (Fig. 1a, b). Young and old mice gained the same absolute quantity of body fat (Fig. 1c) and body weight (Additional file 1: Fig. S1B) on the HFD. Aged mice had, on average, a 17% higher food intake than young mice, in agreement with higher body weight (Additional file 1: Fig. S1C). Respiratory exchange ratios (RER) showed a substrate switch from carbohydrate (high RER) to fat (low RER) in response to the HFD (p_diet<_ 0.0001) (Fig. 1d; Additional file 1: Fig. S1D). After adjustment for body weight, higher energy expenditure was observed in mice fed a HFD vs LFD independently of age (p_diet_ < 0.0001, Fig. 1e). Mouse physical activity did not differ between the age groups but was decreased by the HFD (p_diet_ < 0.0001; Additional file 1: Fig. S1E). Quadriceps mass is known to decrease with age in C57BL/6J mice (around 30% from 6 to 21 months [16]), but the total lean mass was slightly increased in aged animals (Additional file 1: Fig. S2A). Maximal running time and hanging time were negatively affected by advanced age and further decreased by the HFD (Additional file 1: Fig. S2B-C). These correlated with body weight (Additional file 1: Fig. S2E-F). As a further marker of frailty, grip strength was assessed and showed a 20% decreased by advanced ageing independently of the diet (Additional file 1: Fig. S2D).

In the oral glucose tolerance test (OGTT), the HFD-treated groups were more glucose intolerant than the LFD groups (Fig. 1f, g), while plasma insulin levels were much higher in old animals than in young animals, reaching around 5 ng/mL in HFD old mice (Fig. 1h, i). From fasting insulin and glucose levels, we calculated the HOMA-IR index as a parameter for total body IR. The HOMA-IR was strongly increased by both age and diet (p_age_ = 0.0002_,_ p_diet_ < 0.0001). Interestingly, the HOMA-IR of old mice increased more strongly with the HFD than that of young mice (Fig. 1j). The Muscle Insulin Sensitivity Index (MISI) estimates the rate of glucose removed per minute over average insulin concentration during the OGTT [22]. Based on this parameter, the HFD acted on both age groups to lower the insulin sensitivity, which was further decreased in old animals (p_age_ and p_diet_ < 0.0001) (Fig. 1K). Surprisingly, old mice had decreased levels of non-esterified fatty acids (NEFAs, p_age_ = 0.007) when compared to young animals (Fig. 1l). Plasma TGs were also decreased by advanced age to a higher extent, and an increase of about 30% was observed in both age groups when fed a HFD (p_diet_ = 0.009) (Fig. 1m). No differences were observed in the plasma levels of branched-chain amino acids, previously associated with the development of IR [23] (Additional file 1: Fig. S1F). Altogether, these data show that HFD-induced insulin resistance was exacerbated in the aged animals.

### Extensive lipidome remodelling in the quadriceps of old, but not young mice on a HFD

**Fig. 2:**
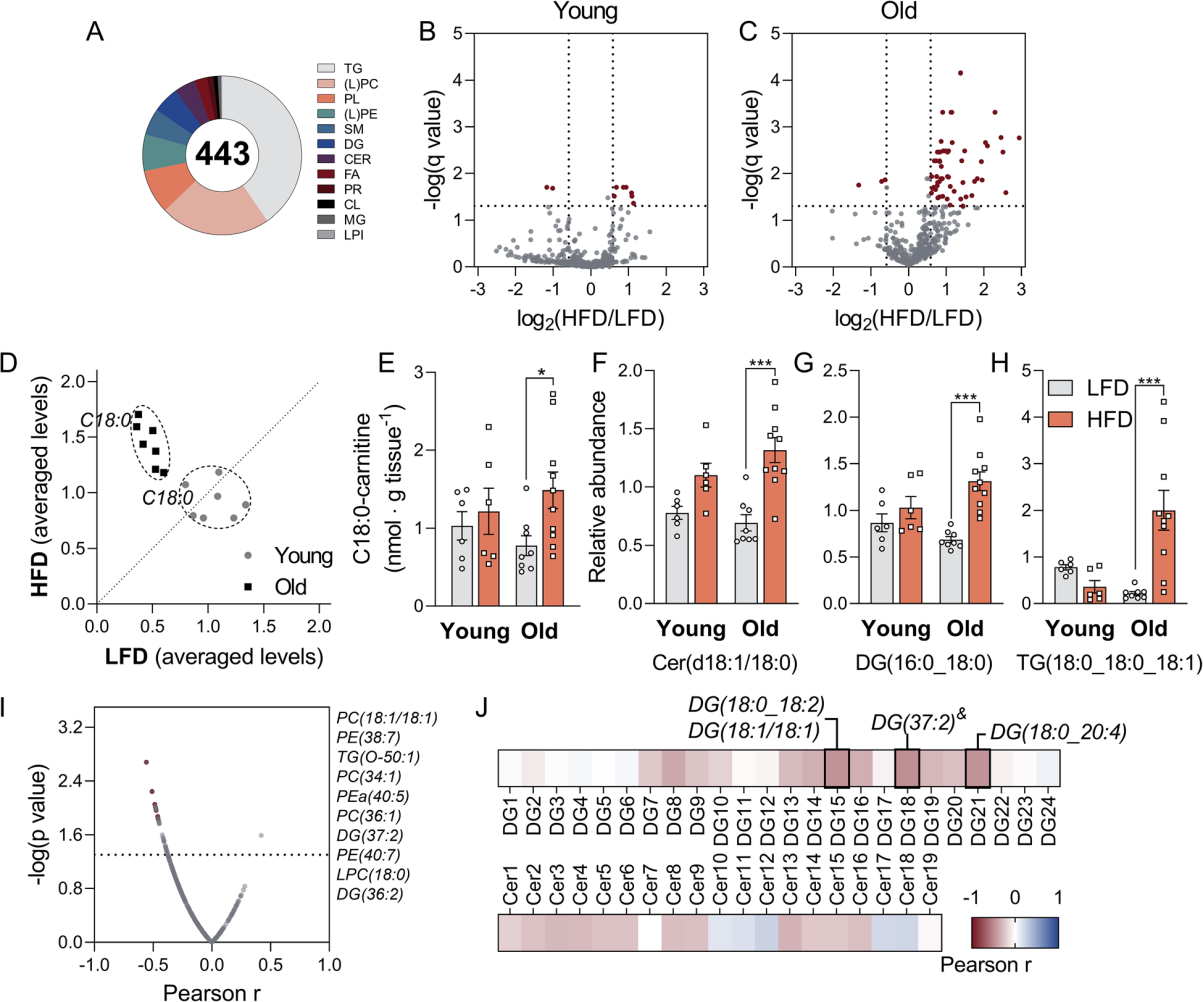
HFD causes extensive lipid remodelling in the quadriceps of aged mice. **a** Number of detected lipid species per category. TG, triglycerides; (L)PC, (lyso)phosphatidylcholine; PL, plasmalogen; (L)PE, (lyso)phosphatidylethanolamine; SM, sphingomyelin; DG, diacylglycerols; CER, ceramides; FA, fatty acids; PR, prenol lipids; CL, cardiolipin; MG, monoacylglycerols; LPI, lysophosphatidylinositol. Volcano plots comparing the effect of a HFD (vs LFD) on young (**b**) and old (**c**) mice, corrected for multiple comparisons with the false discovery rate (FDR) method, with Q = 5%. A fold change threshold was defined as higher than 1.5 or smaller than 0.66. **d** Scatter plot of normalized levels of long-chain acylcarnitines detected via untargeted lipidomics (C14:0, C16:0, C18:0, C20:0, C16:0-OH, C18:0-OH, C18:1-OH). Each dot represents the mean value of a specific metabolite on the HFD (y-axis) vs the LFD (x-axis). Prior to plotting, the levels of each acylcarnitine were normalized to the average value of the same acylcarnitine in all animals. Metabolites along the dashed line are not regulated by the diet (mean LFD = mean HFD), while metabolites above or below the line are up- or downregulated, respectively, by the HFD. **e** Concentrations of C18:0-carnitine detected by LC-MS. **f**–**h** Comparison between diets and age for Cer(d18:1/18:0), DG(16:0_18:0) and TG(18:0_18:0_18:1), respectively. **I** Pearson correlation coefficients (r) between detected lipid species and MISI (x-axis) plotted against -log(p-value) (y-axis). The top 10 lipids with the highest correlation coefficients are specified next to the figure. **J** Heatmap for Pearson correlation coefficients for detected DGs and Cers (from all groups) vs MISI (^&^low signal lipid, only summed structure information). Statistically significant correlations (p < 0.05) are highlighted. Data are shown as mean ± SEM, n = 6 (young groups) or 10 (old groups). Statistical analysis was conducted for each group according to the “Materials and Methods” section; ^###^*p* < 0.001 (old vs young), ^##^*p* < 0.01 (old vs young), ^#^*p* < 0.05 (old vs young), ***p < 0.001 (LFD vs HFD), **p < 0.01 (LFD vs HFD), *p < 0.05 (LFD vs HFD). Supporting data values can be found in Additional file 10 and lipidomics dataset in Additional file 3.

To test whether the susceptibility of aged mice to develop IR is associated with changes in the skeletal muscle lipidome, we used an untargeted lipidomics approach. In total, we detected 443 lipid species in the quadriceps samples (Fig. 2a; Additional file 3: full lipidomics dataset). In young mice, the HFD caused only minor changes in the lipidome, with 7 lipid species mildly increased and 2 lipid species decreased (Fig. 2, Additional file 2: Supp. Table 2). In contrast, in old mice, a substantial number of 58 lipid species were higher in the HFD group than in the LFD group, and 3 lipid species were decreased (Fig. 2c, Additional file 2: Supp. Table 2). A lipid class that was specifically upregulated in old mice on HFD comprised long-chain acylcarnitines (Fig. 2d). This was confirmed by targeted LC-MS for C18:0-carnitine (Fig. 2e, Additional file 2: Supp. Table 3). Such accumulation of long-chain acylcarnitines could represent an imbalance in old mice between uptake and consumption of fatty acids. This could then drive the conversion into complex lipid species that cause IR. Cer(d18:1/18:0) was previously demonstrated to induce IR, specifically in the muscle [24]. This lipid was increased by the HFD in our study in an age-dependent manner (Fig. 2f). Overall, C18:0 side chains were overrepresented in the HFD-induced lipids (Additional file 2: Supp. Table 2). For instance, the diacylglycerol DG(16:0_18:0) was induced by the HFD in old mice (Fig. 2g). Out of the 11 TGs increased by the HFD, all were increased only in old mice and TG(18:0_18:0_18:1) showed the highest fold change (7.7; Fig. 2h). H&E staining of the quadriceps sections indicated that the HFD increased the infiltration of adipocytes into the quadriceps. Aged mice had a higher level of fat infiltration than their young counterparts together with a high presence of vacuolization, indicative of intramyocellular lipid accumulation. Such vacuolization was absent from the quadriceps of young mice (Additional file 1: Fig. S3A-B).

Among the detected lipids, 39 species correlated negatively with the MISI index with *p* < 0.05 (Figs. 1k and 2i). PC(18:1/18:1) had the strongest correlation (r = − 0.56, p = 0.002, Additional file 1: Fig. S3C). A direct role of this phosphatidylcholine, however, has not been previously addressed. Given the previously established roles of Cers and DGs on the insulin signalling pathway [1, 4], we focused on the association between the detected Cers and DGs and the MISI index. We found three DGs and no Cer significantly negatively associated with MISI (Fig. 2j). In conclusion, we found that old mice were more prone than young mice to accumulate lipids in the quadriceps in response to a HFD, particularly C18:0-containing species, and that this correlated with the MISI index.

### Mouse quadriceps display decreased mitochondrial flexibility during ageing

**Fig. 3:**
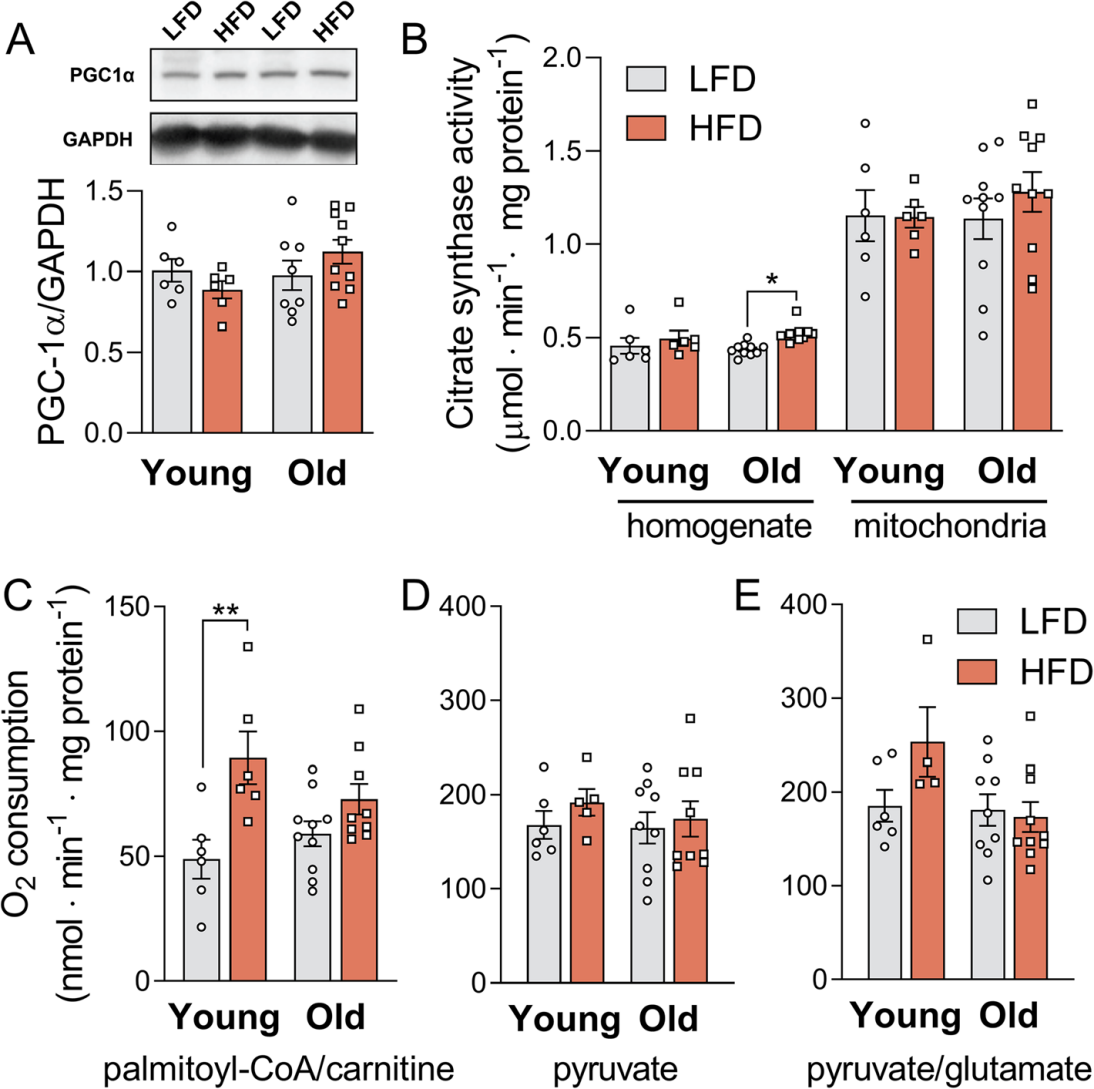
A HFD increases mitochondrial capacity to oxidize fatty acids only in young mice. **a** Total PGC1α protein content in quadriceps homogenates normalized by glyceraldehyde 3-phosphate dehydrogenase (GAPDH) levels. **b** Citrate synthase activities measured in quadriceps homogenates and mitochondria-enriched suspensions.  Mitochondrial O_2_ consumption fluxes were corrected for mitochondria enrichment of suspensions using the ratio between citrate synthase activity (CS) in the mitochondria vs in total homogenate (R = CS_mito_/CS_homog_). Maximal ADP-stimulated O_2_ consumption per tissue protein is shown for palmitoyl-CoA and carnitine (**c**), pyruvate (**d**) and pyruvate and glutamate (**e**) as substrates, all in the presence of malate. Data are shown as mean ± SEM and were analysed using two-way ANOVA. Multiple comparisons were performed using Tukey’s correction, n = 4–6 (young groups) or 8–10 (old groups); **p < 0.01 (HFD vs LFD), *p < 0.05 (HFD vs LFD), matched ages. Supporting data values can be found in Additional file 10

The observed lipid accumulation in old mice on a HFD suggests that oxidation cannot meet the increased lipid supply in these animals. To assess the muscle oxidative capacity, we analysed the mitochondrial content and function. The protein levels of the peroxisome proliferator-activated receptor γ coactivator 1-alpha (PGC-1α), a regulator of mitochondrial biogenesis, were unchanged among the groups (Fig. 3a). Citrate synthase activity in cell homogenates (CS), a marker of mitochondrial mass, was not changed by age and hardly affected by the diet (Fig. 3b). Also in isolated mitochondria, the CS activity remained unaltered (Fig. 3b). O_2_ consumption was measured in isolated mitochondria (Table 1) and then expressed per total tissue protein (Fig. 3c–e). Young mice had an 83% higher oxidative capacity towards palmitoyl-CoA on a HFD than on a LFD (*p* < 0.01). In contrast, the HFD did not significantly affect oxidative capacity in old mice (Fig. 3c). Diet and age did not affect O_2_ consumption when pyruvate or pyruvate with glutamate were used as substrates (Fig. 3d, e). In contrast, in liver mitochondria, respiration on either pyruvate or palmitoyl-CoA was mildly increased by age (p_age_ < 0.05), but not modulated by diet (Additional file 1: Fig. S4a-b). Together, these results point to specific alterations in the β-oxidation pathway, rather than in the TCA cycle or electron transport chain (ETC), since the latter are shared by all three substrates. Apparently, ageing does not cause mitochondrial deficiency per se but instead blunts the flexibility of the β-oxidation pathway to respond to the different diets.

**Table 1:** O_2_ consumption rates (states 3 and 4) for isolated mitochondria from quadriceps

Tissue		Substrates					
		Pyruvate		Pyruvate/glutamate		Palmitoyl-CoA/carnitine	
Quadriceps		Young	Old	Young	Old	Young	Old
State 3	**LFD**	428 ± 56	424 ± 58	476 ± 68	458 ± 57	131 ± 29	153 ± 20
	**HFD**	524 ± 56	415 ± 48	627 ± 77	419 ± 43	204 ± 14*	184 ± 20*
State 4	**LFD**	20.9 ± 5.9	20.2 ± 4.5	21.9 ± 6.8	30.2 ± 9.7	36.1 ± 10.2	26.5 ± 3.6
	**HFD**	20.2 ± 2.6	18.9 ± 3.7	27.6 ± 8.1	14.9 ± 2.2	35.6 ± 4.4	26.1 ± 5.1
RCR	**LFD**	26.0 ± 4.7	26.3 ± 3.7	38.6 ± 13.1	25.3 ± 3.9	4.2 ± 0.8	6.1 ± 0.5
	**HFD**	28.2 ± 5.1	27.0 ± 4.0	30.3 ± 10.9	31.8 ± 3.9	5.9 ± 0.5	7.6 ± 0.8

All substrates were added in the presence of malate. Values are shown as mean ± SEM (nmol ⋅ mg mitochondrial protein^−1^ ⋅ min^−1^). State 3: ADP-stimulated maximal respiration. State 4: carboxyatractyloside-induced inhibition of ATP synthesis (via ATP-ADP translocase inhibition), RCR: respiratory control ratio (State 3/State 4), *p_diet_ = 0.025

### Ageing downregulates CPT1B despite HFD-induced β-oxidation remodelling

**Fig. 4:**
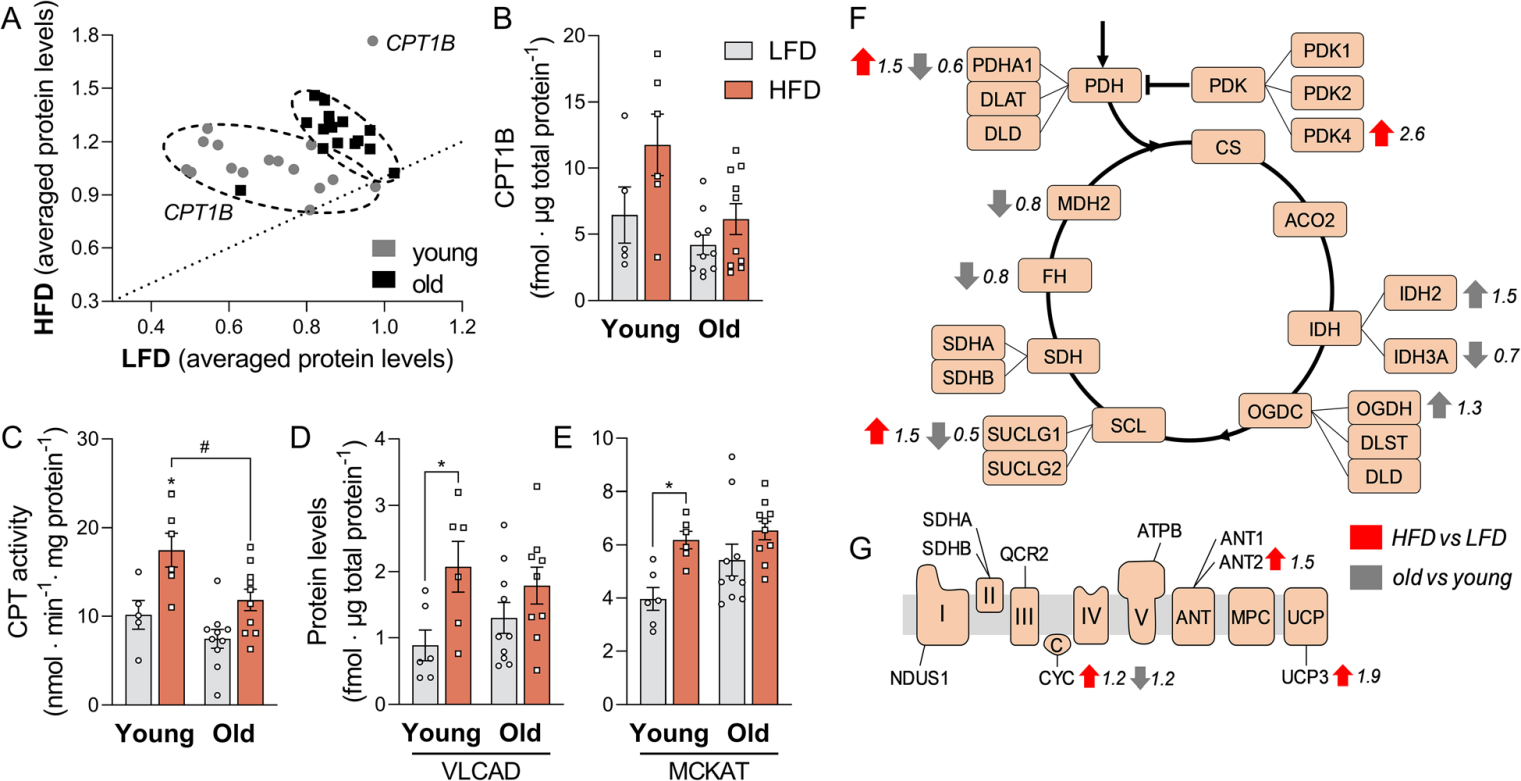
Upregulation of β-oxidation proteins by the HFD, despite accompanying loss of CPT1B with ageing. **a** Scatter plot of normalized levels of β-oxidation proteins detected in mouse quadriceps via targeted proteomics Each dot represents the mean value of a specific protein on the HFD (y-axis) vs the LFD (x-axis). Prior to plotting, the levels of each protein were normalized to the average value of the same protein in all animals. Proteins along the dashed line are not regulated by the diet (mean LFD = mean HFD), while proteins above or below the line are up- or downregulated, respectively, by the HFD. CPT1B, carnitine palmitoyltransferase 1B. **b** Relative CPT1B content in all groups. **c** Total CPT activity measured in mitochondrial samples and corrected for the enrichment of the preparation (CS_mito_/CS_homog_) as mentioned in Fig. 3D. **d**, **e** Quantification of very-long-chain acyl-CoA dehydrogenase (VLCAD) and medium-chain ketoacyl-CoA thiolase (MCKAT). **f**, **g** Schematic representation of the effects of age and diet on the TCA cycle and ETC, respectively. Each box represents a protein that has been measured by our method, and the specific isoforms or subunits that have been measured are annotated. Red arrows represent the effect of diet (HFD vs LFD) while the grey arrows indicate the age effect (old vs young) based on significant two-way ANOVA results. PDH, pyruvate dehydrogenase complex; CS, citrate synthase; ACO2, aconitase; IDH, isocitrate dehydrogenase; OGCD, α-ketoglutarate dehydrogenase complex; SCL, succinyl-CoA ligase; SDH, succinate dehydrogenase (complex II); FH, fumarate dehydrogenase; MDH2, malate dehydrogenase; ANT, adenine nucleotide translocator; MPC, mitochondrial phosphate carrier protein; UCP, uncoupling protein. Data are shown as mean ± SEM and were analysed using two-way ANOVA, and multiple comparisons were performed using Tukey’s correction. N = 6–10 per group; **p < 0.01 (HFD vs LFD), *p < 0.05 (HFD vs LFD), matched ages, and ^#^*p* < 0.05 (old vs young), matched diets. Supporting data values can be found in Additional file 10 and proteomics dataset in Additional files 4 and 5

To explain the loss of flexibility of mitochondrial β-oxidation, we took a targeted and quantitative proteomics approach, based on isotopically labelled peptide standards [25]. Sixteen mitochondrial β-oxidation proteins were quantified, 14 of which were increased by the HFD relative to the LFD in both age groups (p_age_ < 0.05) (Fig. 4a, Additional files 4, 5: full proteomics dataset and average values, respectively). Proteins clustered together per age group, with the highest protein concentrations in old mice (Fig. 4a). The exception was carnitine palmitoyltransferase 1B (CPT1B, muscle isoform); both protein levels and activity were increased by the HFD, but decreased with advanced age (p_age_ and p_diet_ < 0.05) (Fig. 4b, c). Closer inspection of both CPT1B protein concentration and enzyme activity showed a similar loss of flexibility that was earlier observed in the lipidome: in young mice on HFD protein and activity reached levels around 90% and 50% higher than in old mice, respectively (Fig. 4b, c). This loss of flexibility in old mice was also observed for very long-chain acyl-CoA dehydrogenase (VLCAD) and medium-chain ketoacyl-CoA thiolase (MCKAT), two other key β-oxidation enzymes [19, 26] that were increased by the HFD mostly in younger animals (Fig. 4d, e).

Further analysis of the downstream pathways revealed minor changes in the TCA cycle and ETC (Fig. 4f, g) by age. Pyruvate dehydrogenase kinase 4 was increased by HFD (PDK4, FC: 2.6), as reported previously [27]. Increased PDK4 expression leads to inhibition of pyruvate dehydrogenase (PDH) by phosphorylation of the PDH-E1α subunit [27] and contributes to the shift from carbohydrate to fatty acid utilization. Here, the effect was, however, counteracted by an upregulation of the PDH-E1α (PDHA1, FC: 1.5) and the net effect of both changes is unclear. Uncoupling protein 3 (UCP3) was upregulated by the diet (FC: 1.9), also a known effect of increased fatty acid availability [28]. The concentrations of TCA cycle intermediates were barely affected by age and/or diet (Additional file 1: Fig. S5A-E), corroborating the notion that changes in TCA cycle do not play a major role in the observed phenotype. In conclusion, the decrease in flexibility of the mitochondrial β-oxidation pathway could be traced down to a loss of flexibility and content of CPT1B with age in mice fed a HFD, with possibly a contribution by VLCAD and MCKAT.

### HFD-proteome remodelling is overruled by low and less flexible CPT1B in aged animals

**Fig. 5:**
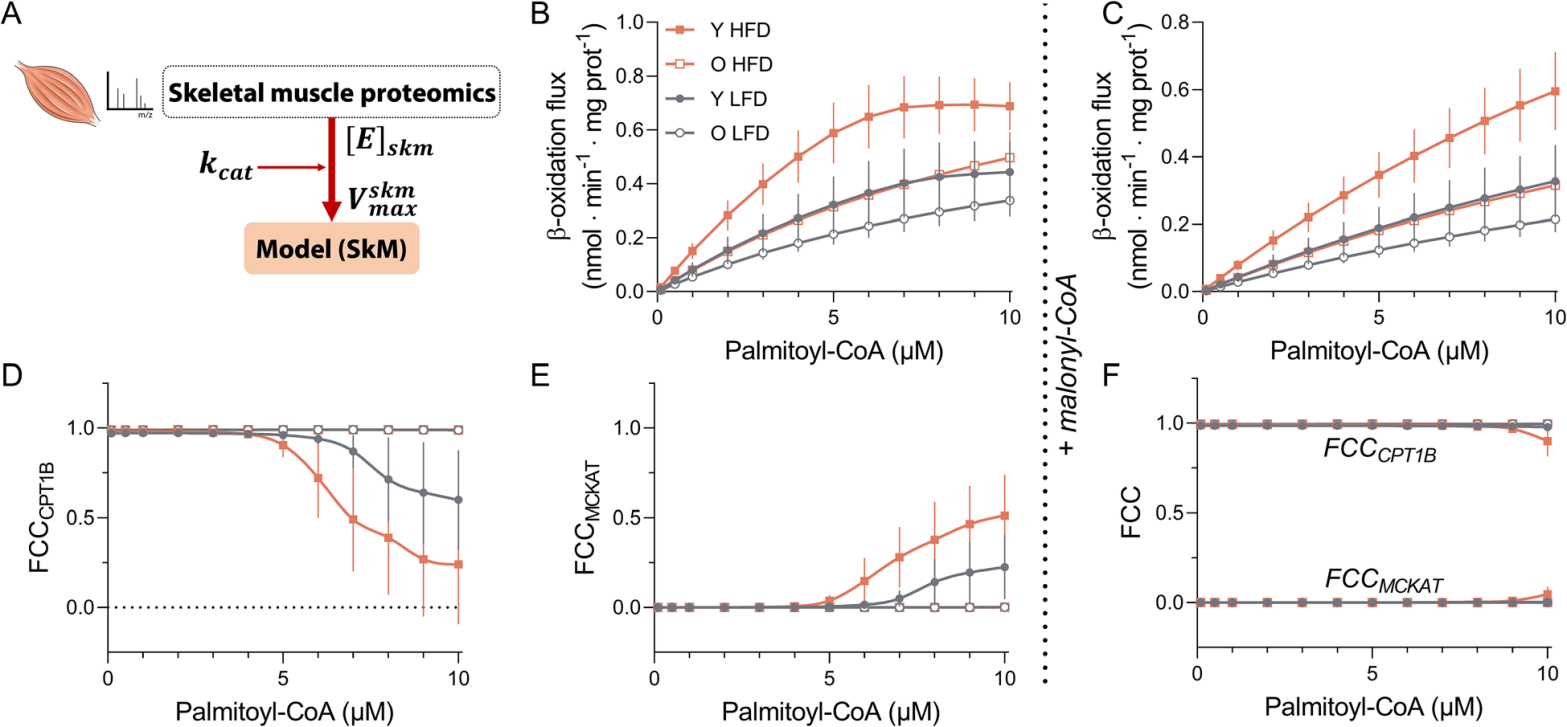
In silico β-oxidation simulations confirm flux dependence on CPT1B. **a** Schematic representation of the strategy used to parameterize the computational model of mitochondrial β-oxidation to the skeletal muscle. Proteomics data from the liver previously published were used to estimate k_cat_s for the proteins involved in the pathway and applied to proteomics data obtained from quadriceps samples to calculate V_max_s for each animal. **b**: Estimated steady-state β-oxidation fluxes (defined as the fluxes through CPT1B) based on in vivo-like conditions for different concentrations of substrate (palmitoyl-CoA) ranging from 0.1 to 10 μM and normalized for total homogenate protein. **c** Estimated steady-state β-oxidation-fluxes in the presence of 0.2 μM malonyl-CoA. Flux control coefficients (FCC) were calculated for the same substrate range. FCC is defined as ∂J_ss_/∂E_i_, which represents to what extent an enzyme ‘i’ (E_i_) can alter the steady-state flux of a pathway (J_ss_). FCCs for CPT1B (**d**) and MCKAT (**e**) are depicted. **f** FCCs for CPT1B and MCKAT in the presence of 0.2 μM malonyl-CoA. All simulations were performed for the V_max_s estimated for each mouse in the dataset. Y stands for young (LFD: grey full circles; HFD: orange full squares) and O for old (LFD: grey open circles; HFD: orange open squares). Data are shown as mean ± SEM and were analysed using two-way ANOVA, n = 5–6 (young groups) or 10 (old groups). A complete description of the model and the assumptions made can be found in Additional file 6. Supporting data values can be found in Additional file 10.

While there was a substantial upregulation of most fatty acid oxidation enzymes in both HFD-treated groups, CPT1B content and activity were significantly lower in old than in young animals. This raised the question of what is the net in vivo effect of these opposing responses in the proteome. We tested this computationally, using a dynamic β-oxidation model previously developed by our group [29]. To parameterize it to skeletal-muscle mitochondria, the proteomics data were used (see Additional file 6 for details). A version of the β-oxidation model was created for each individual mouse in the dataset. The steady-state palmitoyl-CoA consumption flux, representing the conversion of palmitoyl-CoA to acetyl-CoA, was plotted as a measure of the β-oxidation. Palmitoyl-CoA concentrations were chosen within the likely physiological range of skeletal-muscle tissue [30] (Fig. 5b). The results indicated a significant diet and age effect (p_age_ < 0.05, p_diet_ < 0.05), and a stronger upregulation of the β-oxidation fluxes by the HFD in young mice, reaching values 75% higher than in aged mice in the given substrate range. In vivo, however, CPT1B is inhibited by endogenous malonyl-CoA [26]. Acetyl-CoA carboxylase (ACC) levels, as well as its active phosphorylated form (pACC) which synthesizes malonyl-CoA, remained unchanged in all four groups. Malonyl-CoA decarboxylase (MCD) levels did not differ either (Additional file 1: Fig. S6A-D). Therefore, we applied the same malonyl-CoA concentration to all groups. As expected, the addition of malonyl-CoA reduced the β-oxidation fluxes in the simulations (Fig. 5c), but resulted in a similar pattern among groups as observed without malonyl-CoA (Fig. 5b). The model showed that age tended to decrease the C16-acylcarnitine levels, due to the lower CPT1 activity, while increased substrate concentration tended to increase C16-acylcarnitine (Additional file 1: Fig. S6E). This suggests that the elevated long-chain acylcarnitine levels measured in the quadriceps of old HFD animals (Fig. 2d, e) are primarily caused by the higher substrate influx.

Flux control coefficients (FCC) quantify how the pathway flux is modulated when the maximal capacity of a particular enzyme is changed [31], i.e. to which extent the enzyme controls the flux. In the absence of malonyl-CoA, the FCC_CPT1B_ remained approximately constant (~ 1) for old animals, implying that this was nearly the sole rate-limiting step within the analysed concentration range of palmitoyl-CoA. In contrast, FCC_CPT1B_ tended to decrease with a palmitoyl-CoA overload in young animals (Fig. 5d). This drop was compensated by increased control in MCKAT (Fig. 5e). The control of CPT1B is, however, expected to remain close to 1 within a wider range of palmitoyl-CoA in vivo due to the action of malonyl-CoA [26], which was confirmed in our simulations (Fig. 5f). These results suggest that, despite the remodelling of most β-oxidation proteins by the HFD in both age groups, the lack of flexibility at the level of CPT1B overruled this regulation and constrained the fluxes through the pathway specifically in aged animals.

### The glycolytic proteome shows less flexibility in aged mice

**Fig. 6:**
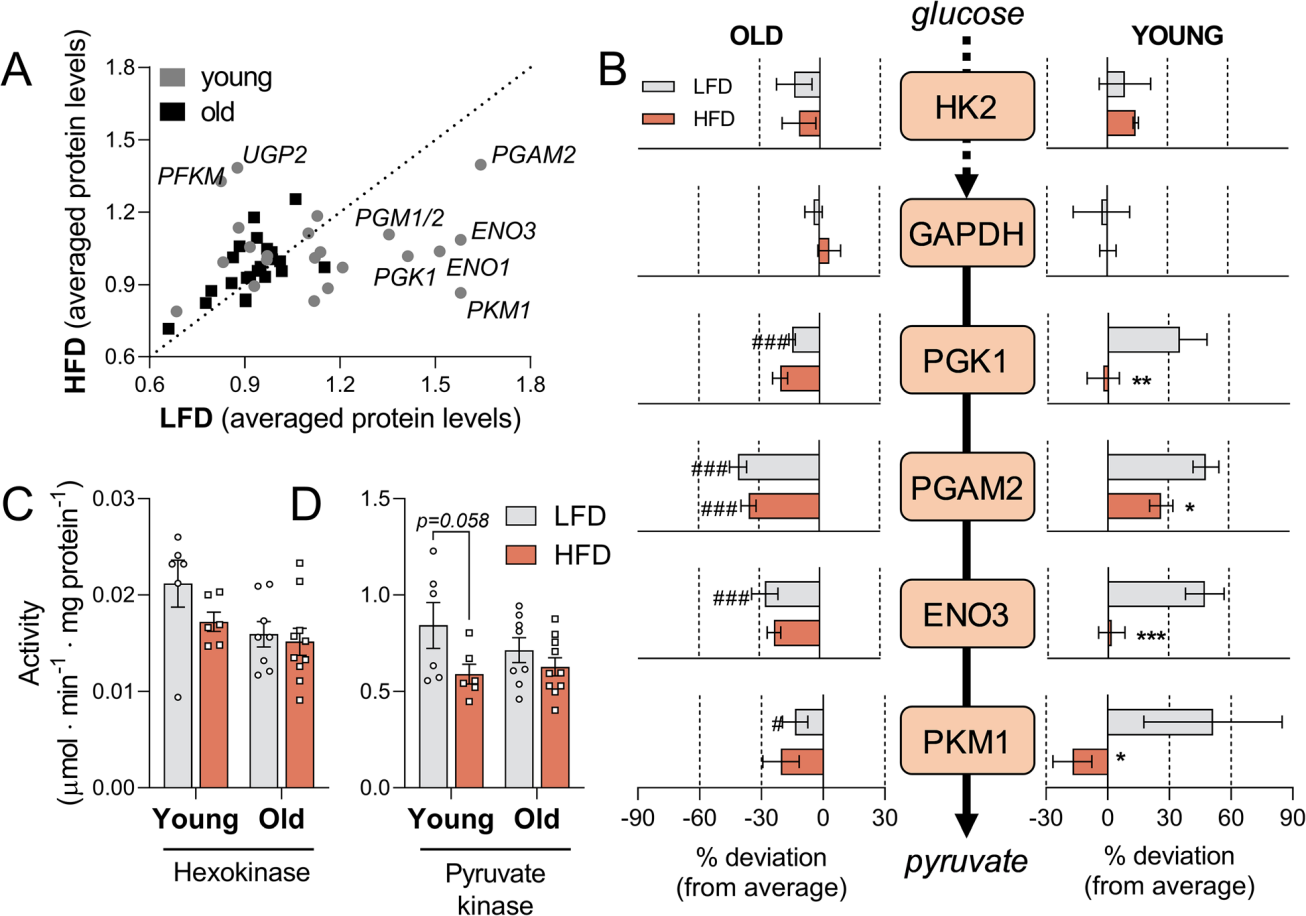
Glycolytic proteins are downregulated in aged mice, which also shows less flexibility to the diet. **a** Scatter plot for proteomic targets involved in glucose metabolism (glycolysis, pentose phosphate pathway and glycogen metabolism) measured in quadriceps samples. Each dot represents the mean value of a specific protein on the HFD (y-axis) vs the LFD (x-axis). Prior to plotting, the levels of each protein were normalized to the average value of the same protein in all animals. Proteins along the dashed line are not regulated by the diet (mean LFD = mean HFD), while proteins above or below the line are up- or downregulated, respectively, by the HFD. PFKM, phosphofructokinase, muscle type; UGP2, UDP-glucose pyrophosphorylase 2; PGAM2, phosphoglycerate mutase 2; ENO3, enolase 3 (muscle isoform); ENO1, enolase 1; PGK1, phosphoglycerate kinase 1; PGM1/2, phosphoglucomutase isoforms 1 and 2; PKM1, pyruvate kinase, muscle isoform. **b** Simplified glycolysis scheme. The depicted values represent the % deviation from the average of the 4 groups (young/old and LFD/HFD) for each protein. The values are shown for old animals (panel on the left) and young animals (right panel). **c**, **d** Hexokinase and pyruvate kinase activities measured in quadriceps homogenates. Data are shown as mean ± SEM and were analysed using two-way ANOVA and multiple comparisons were performed using Tukey’s correction, n = 5–6 (young groups) or 10 (old groups), ^###^p < 0.001 (old vs young), ^##^*p* < 0.01 (old vs young), ^#^*p* < 0.05 (old vs young), matched diets, ***p < 0.001 (LFD vs HFD), **p < 0.01 (LFD vs HFD), *p < 0.05 (LFD vs HFD), matched ages. Supporting data values can be found in Additional file 10 and proteomics dataset in Additional files 4 and 5.

Finally, we investigated the flexibility of glucose metabolism at the proteome level. Again, using isotopically labelled standards peptides, we quantified 40 peptides, spanning 31 proteins involved in glycolysis and glycogen metabolism (Additional files 4, 5). In old mice, the proteins in glucose metabolism did not respond to the diet (Fig. 6a). In contrast, in young mice, 11 proteins responded to the diet, as reflected by a deviation from the identity line in Fig. 6a, 4 of which were confirmed to be statistically significant and involved in lower glycolysis (Fig. 6b). These enzymes, namely enolase 3 (ENO3, muscle isoform), phosphoglycerate kinase 1 (PGK1), phosphoglycerate mutase 2 (PGAM2), phosphoglucomutase 1/2 (PGM1/2) and pyruvate kinase (PKM1, muscle isoform) followed a similar pattern among the tested groups: decreased by the HFD in young mice (therefore flexible to the diet) and decreased levels at old vs young age (p_age_ < 0.05) (Fig. 6b). Glyceraldehyde 3-phosphate dehydrogenase (GAPDH, Fig. 6b) was modulated by neither age nor diet. Besides control at the level of ATP demand, skeletal muscle glycolysis is also controlled by hexokinase (main muscle isoform in mice: HK2) [32]. Age decreased HK2 content in quadriceps by about 25% (p_age_ = 0.026) (Fig. 6b). The proteomics results were also reflected at the level of enzyme activities. Hexokinase activity was reduced by 18.5% by advanced age (p_age_ = 0.03) (Fig. 6c). Pyruvate kinase activity was modulated by diet (p_diet_ = 0.02) and tended to be reduced by the HFD relative to the LFD, specifically in young animals (Fig. 6d). In the glycogen metabolism pathway, glycogen debranching enzyme (AGL), glycogen phosphorylase (PYGM) and phosphoglucomutase-1 (PGM) were reduced by advanced age, suggesting that glycogen breakdown could be adversely impacted by age (Additional File 5). Together, these results demonstrate that glucose metabolism in young mice was more flexible to changes in diet and that aged mice had a lower content of glycolytic proteins. Altogether, this is in line with reduced glucose utilization by the skeletal muscle in older animals and could further contribute to the IR phenotype.

## Discussion

This study addressed the combined effects of age and diet on skeletal muscle lipid handling and its implications for IR. We found that neither the HFD nor ageing alone led to profound lipid accumulation in the skeletal muscle, but only the combination of both. We did not observe a general decline of mitochondrial function in the quadriceps of old animals, but rather a specific failure to upregulate the β-oxidation capacity in response to the HFD, which was traced back to CPT1B. Finally, old animals had lower levels of key glycolytic proteins, which were also less responsive to the diet and could further contribute to a less flexible glucose handling.

Whereas we obtained unambiguous results concerning mitochondrial lipid handling, its relation to IR is more complex. Age strongly decreased the peripheral insulin sensitivity as quantified by the MISI index and this was further exacerbated by the HFD. The loss of peripheral insulin sensitivity in the LFD aged group, without concomitant lipid accumulation, may be explained by the progressive loss of muscle mass that accompanies ageing irrespective of diet [16, 33]. Consistent with this, old mice performed worse in functional running and hanging tests than their young counterparts. Diet also affected performance, but this was correlated to body weight and may not be directly related to muscle wasting per se.

Insulin-resistant skeletal muscle displays a C18:0-ceramide signature across different species [24, 34, 35], a lipid species that directly causes IR in mice [24]. Consistent with this finding, we have found a higher increase of long-chain ceramides such as C18:0 and C22:0 in old animals than in young animals in response to a HFD. Nonetheless, we failed to find a direct association between C18:0-ceramide and the MISI index. This may be related to quadriceps not being the exclusive contributor to peripheral insulin sensitivity. It may also be due, however, to modifying the effects of other lipids. We found, for instance, significant negative correlations between C18:0-containing DGs and MISI (Fig. 2m), which could indicate that C18:0-ceramide acts in synergy with DGs to cause IR. Two of the 5 most abundant DGs in the tissue correlated with MISI, namely DG (36:2) and DG(18:0_20:4). Interestingly, in the total membrane fraction from the vastus lateralis (which belongs to the quadriceps group), DG(C18:0_20:4) also correlated negatively with insulin sensitivity in obese and diabetic individuals [36]. However, a few mechanistic studies have pointed to a causal relationship between specific DG species and IR [37]. Ceramides and DGs are known to inhibit specific steps of the insulin transduction cascade in the skeletal muscle [1]. As a limitation, the animals used for this study were fasted and not stimulated with insulin prior to sacrifice. For this reason, we have not included data on basal AKT phosphorylation, which is not necessarily diminished in insulin-resistant animals in the basal state [38].

Diet and age did not have a major influence on mitochondrial biogenesis and content, as indicated by PGC-1α and citrate synthase activity, respectively. However, the proteome remodelling of young mice increased the β-oxidation capacity of quadriceps mitochondria in response to a HFD. This phenomenon appears to be a physiological response to increased lipid availability [16, 39] and may compensate for the fatty acid overload, thereby preventing lipid accumulation [9, 13]. Proteome remodelling was specific for the β-oxidation pathway and was not observed for the TCA cycle and ETC, in agreement with previous studies [40]. This HFD-induced upregulation of oxidative capacity was blunted in aged animals (FC 1.8 in young vs 1.2 in old mice), despite a general upregulation of proteins involved in the β-oxidation.

The most prominent exception in the β-oxidation proteome was CPT1B, which showed decreased content and activity in old compared to young mice, and was hardly upregulated by the HFD in old mice. This observation is consistent with mRNA data from the quadriceps of aged C57BL mice [41, 42], suggesting that at least part of the defect could be at the transcriptional level. CPT1B has, in addition, been shown to be reduced by ageing in fast IIa fibres from human vastus lateralis [43] and to be decreased in obese vs lean individuals [44]. In contrast, higher expression of CPT1B was observed in the skeletal muscle of aged human individuals that were more insulin sensitive (vs less sensitive) [45], which corroborates our findings. Moreover, CPT1B overexpression has been reported to improve HFD-induced insulin resistance in a rat model [12]. In agreement with previous findings [26], we observed a shift of flux control from CPT1B at low levels of palmitoyl-CoA towards MCKAT at high levels of palmitoyl-CoA, particularly in the absence of malonyl-CoA. This may indicate that the pathway operates close to a shift of flux control, yet the integrated results indicate CPT1B as the key enzyme in the mitochondrial flexibility under the studied conditions. Therefore, an age-dependent CPT1B decline (reducing the demand for fatty acids) could be the culprit to lead to lipid accumulation when combined with increased fatty acid supply. The latter can be caused by the HFD itself, but might be aggravated by the upregulation of CD36 (fatty acid transport facilitator) that accompanies the HFD [20]. We therefore propose that the reduction of CPT1B with age specifically in the skeletal muscle plays a key role to predispose individuals to diet-induced insulin resistance. We have found no evidence that a similar mechanism would play a role in the liver, based on repirometry data. Moreover, RNASeq data from white adipose tissue has not shown differences in the CPT1B expression with age either in humans or mice [46, 47].

Although most studies focus on the relationship between lipids and insulin signalling [1], glucose utilization in response to insulin can be partly regulated via the content of glycolytic enzymes. Upon insulin stimulation or exercise, for instance, the control of glucose utilization can shift from glucose uptake towards hexokinase [32]. In the present study, we observed decreased content and flexibility of several glycolytic proteins and activities in aged animals, including enzymes that may share the control of flux, such as hexokinase and pyruvate kinase [32, 48]. These results are in line with the previously reported trend of decreased levels of enzymes of lower glycolysis in old C57BL/6 mice [43, 49]. Quadriceps is a fast-twitch muscle in which type II fibres predominate. We found earlier that the contribution of type IIb fast glycolytic fibres decreased with age and by a high-fat/sucrose diet, while that of type IIa fast-twitch oxidative-glycolytic fibres increased [16]. The changes that we found in the proteome, at the level of both lipid and glucose metabolism, are in agreement with such modulation of fibre type.

## Conclusion

We focused on the alterations in the metabolic pathways that explain the decline of flexibility in lipid and glucose handling with age. We propose CPT1B to be the main driver of the loss of flexibility to the HFD in aged mice. Future studies should elucidate the underlying transcriptional or translational mechanisms behind its regulation with age, as well as its causative role on the flexibility to the diet. Strategies that enhance muscle CPT1B activity could, nonetheless, offer the potential to attenuate lipid accumulation and consequently lipid-induced insulin resistance in the elderly.

## Methods

### Animals and diet

Male C57BL/6J mice were obtained from the Mouse Clinic for Cancer and Aging of the University of Groningen and kept under standard housing conditions with ad libitum access to food (rodent chow diet (RM1) SDS Diets, Woerden, The Netherlands) and water, a 12-h light/dark cycle and a temperature-controlled environment. The mouse experiments here reported have been part of a larger study performed at the University Medical Center Groningen, the Netherlands. Therefore, both old and young control mice on a low-fat diet (LFD) correspond to mice on a medium-protein diet (MP), as reported by Dommerholt et. al, 2020 [46]. Prior to the experiment, 3- and 18-month-old mice (referred to as young and old, respectively) mice were put on a 2-week run-in period, containing a control diet (10% fat) to normalize microbial health. In order to assign animals to either a low- or high-fat diet (HFD), old animals were normalized for body weight and 4 h fasting glucose, insulin, and cholesterol levels before division into experimental groups. Young animals were randomly placed on one of the experimental diets. The diets consisted on either a 20% LFD or 60% HFD (based on the AIN-93G breeding diet (D10012G), Open Source Diets, New Brunswick, NJ, USA, Additional File 2: Supp. Table 1) for 12 weeks. One week before experimental diets were introduced, mice were housed individually (Additional file 1, Fig. S1A: detailed timeline). Animals were weighed weekly and body composition was determined, both during the run-in period and after 8 weeks of the experimental diet, using a Bruker’s Minispec Whole Body Composition Analyzer. Food intake was measured several times over a 72-h period. Energy expenditure (EE) and respiratory exchange ratio (RER) were measured using LabMaster metabolic cages (TSE Systems, Bad Homburg, Germany). Mice were acclimatized to the cages overnight before measurements were recorded for 48 h. The system measured O_2_ consumption and CO_2_ production to calculate the EE. The RER was determined as the ratio of the volumes of produced CO_2_ vs consumed O_2_. Infrared beams recorded locomotor activity according to the number of beam break events in the horizontal (x) and vertical (z) plane [46]. In terminal blood samples, plasma triglycerides, total cholesterol and free fatty acids were determined with commercially available kits (Roche Diagnostics, Mannheim, Germany and DiaSys DiagnosticSystems, Holzheim, Germany). Amino acid levels were quantified using cation-exchange high-performance liquid chromatography followed by post-column ninhydrin derivatization, on a Biochrom30 analyzer (Pharmacia Biotech, Cambridge, UK) [50]. Norleucine was used as an internal standard. Lean mass, maximal running time, hanging time and grip strength were measured according to Dommerholt et al. [46].

### Assessment of glucose and insulin tolerance

To determine the changes in glucose homeostasis and insulin sensitivity, an oral glucose tolerance test (OGTT) was performed following oral administration of 1.5 g/kg body weight D-glucose after overnight (10 h) fasting. A 25% glucose solution (w/v) was given by oral gavage. Blood glucose levels were measured at 0, 5, 15, 30, 45, 60, 90 and 120 min after administration of the glucose bolus, using a OneTouch Select Plus glucose meter (Lifescan, Zug, Switzerland).

To measure insulin response, additional blood spots were taken at time points 0, 5, 15, 30, 60 and 120 min after glucose administration. Insulin was extracted from the blood spots and concentrations were determined using the rat insulin ELISA kit from Crystal Chem (Cat. 90010, Zaandam, The Netherlands) and a mouse insulin standard (Cat. 90020, Zaandam, The Netherlands) according to the manufacturer’s protocol. Homeostatic Model Assessment of Insulin Resistance (HOMA-IR) indexes were calculated as previously described using both the fasting insulin and fasting glucose levels and corrected for mice [51].

To avoid reinfusing blood cells as performed in frequently sampling protocols, the relatively small datasets of blood glucose and plasma insulin over time were fitted to equation 1 below with the use of SAAM II v2.1 (The Epsilon Group, Charlottesville, VA, USA), where C_t_ is the concentration of glucose over time. This was performed to generate enough data points for the calculation of the Muscle Insulin Sensitivity Index (MISI). This index has the units of a glucose clearance rate (dC_t_/dt) divided by the average insulin concentration during the OGTT (I), as previously defined [22]. To overcome the need to fit a linear equation to the glucose clearance part of the glucose tolerance curve [22, 52], we obtained the maximal dC_t_/dt after the glucose concentration peak using GraphPad Software Inc., version 8.0, 2018.

1 $$ {C}_t={C}_b+{C}_1\left({e}^{-{k}_{e_1}\cdot t}-{e}^{-{k}_{\mathrm{a}}\cdot t}\right)+{C}_2\cdot {e}^{-{k}_{e_2}\cdot t} $$

### Lipid extraction, LC-MS analysis and data processing

Frozen quadriceps tissue was homogenized in 0.9% NaCl (15% w/v) using a BeadBeater system (Precellys® Evolution, Bertin Technologies). Lipid extraction was performed following the protocol of Matyash et al. [53] with slight modifications. LC-MS grade acetonitrile (ACN), methanol (MeOH), isopropanol (IPA) and chloroform were purchased from Biosolve BV (Valkenswaard, The Netherlands). Ammonium formate, formic acid and methyl tert-butyl ether (MTBE) were purchased from Sigma Aldrich (St. Louis, MO). Various lipid standards were purchased from Avanti Polar Lipids, Inc. (Alabaster, AL). Twenty microlitres of muscle homogenates (~ 3 mg muscle) was mixed with 300 μL MeOH. A defined amount of lipid standards was added to each tube followed by 10-min sonication [composition: 2 nmol of phosphatidylcholine PC(15:0_18:0-d7), 2 nmol lysophosphatidylcholine LPC(18:1-d7), 3 nmol plasmenyl-PC(p18:0_18:1), 6 nmol phosphatidylinositol PI(15:0_18:1-d7), 4 nmol phosphatidylethanolamine PE(15:0_18:1-d9), 6 nmol lyso PE(18:1-d9), 3 nmol plasmenyl-PE(p18:0_18:1-d9), 4 nmol cardiolipin CL(14:0), 2 nmol sphingomyelin SM(18:1-d9), 3 nmol ceramide Cer(d18:1-d7/15:0), 10 nmol monoglyceride MG(18:1-d7), 2 nmol diglyceride DG(15:0_18:1-d7), 2 nmol DG(17:0_17:0-d5), 0.6 nmol triglyceride TG(15:0_18:1-d7_15:0), 0.6 nmol TG(17:0_17:1_17:0-d5) and 10 nmol cholesterol-d7. Subsequently, 1 mL MTBE was added to the mixture and kept under 25 °C on a shaker (900 rpm) for 30 min. Phase separation was induced by adding 190 μL H_2_O. The mixture was then centrifuged at 3000*g* for 10 min, and 850 μL of the upper phase was transferred to a new tube. The re-extraction was performed by adding 600 μL MTBE/MeOH/H_2_O (10:3:2.5, v/v/v) into the lower phase and 500 μL was collected after centrifugation to combine with the previous organic phase. The combined lipid extract solutions were dried in a vacuum centrifuge at 45 °C. Subsequently, dried lipid extracts were resuspended in 50 μL chloroform/MeOH/ H_2_O (60:30:4.5, v/v/v) and further diluted with 150 μL IPA:ACN:H_2_O (2:1:1 v/v/v). Twenty-four microlitres was injected in both positive and negative modes for LC-MS analysis. In addition, 20 μL of lipid solution taken from each sample was combined to generate a quality control pool sample and measured repeatedly throughout the measurements to monitor the technical reproducibility.

LC-MS lipid analysis was performed on an Ultimate 3000 High-Performance UPLC coupled with a Q Exactive MS (Thermo Fisher Scientific, Darmstadt, Germany). Chromatography separation was achieved with an Acquity UPLC CSH column [1.7 μm, 100 × 2.1 mm, (Waters Corporation, Milford, MA)] under 55 °C with a flow rate of 0.4 mL/min. Mobile phase A was composed of H_2_O/ACN 40:60 (v/v), 10 mM ammonium formate and 0.1% formic acid. Mobile phase B contained ACN/IPA 10:90 (v/v) with 10 mM ammonium formate and 0.1% formic acid. The LC gradient ( [54], modified) started with 40% B and raised up to 43% B at 2 min. The percentage of B raised up to 50% in the next 0.1 min and increased to 54% in the next 9.9 min. B raised to 70% in 0.1 min and increased to 99% in 5.9 min and maintained in 99% for 1 min. Subsequently, the percentage of B went back to 40% in 0.1 min, and the system was equilibrated for 3.9 min before the next run started. The MS was configured under both positive and negative modes for data-dependent acquisition. A full MS scan ranging from 200 to 1750 m/z was acquired at resolution 70,000 FWHM (AGC target 1E6, maximum injection time 50 ms) followed by up to 8 MS/MS events with a collision energy at the resolution of 17,500 FWHM. The precursor isolation window was set to 1.5 Da with the dynamic exclusion time of 6 s. The ionization settings were as follows: capillary voltage, + 3.2 kV; capillary temperature, 320 °C; and sheath gas/auxiliary gas, 60/20.

The Thermo Xcalibur® software [(version 3.2.63), Thermo Scientific, Waltham, MA] was used for data acquisition. The Progenesis QI® software (Waters Corporation, Milford, MA) was used for data preprocessing including retention time alignment, peak picking and annotation of the LC-MS data. Peak picking was performed with an absolute ion intensity filter of 200,000 counts. Lipid annotation was performed by searching published lipid databases (Human Metabolome Database and Lipidmaps) based on mass accuracy (< 5 ppm), isotopic similarities, adduct type, MS/MS spectra and elution behaviour. Analysis of the fatty-acyl composition of selected lipids was performed with the Lipidhunter2 software [55]. Lipid intensities were normalized to the intensities of corresponding lipid standards from the same class. In case of the absence of lipid standards, the intensity was normalized to the average of all standards.

LC-MS lipid analysis was performed on an Ultimate 3000 High-Performance UPLC coupled with a Q Exactive MS (Thermo Fisher Scientific, Darmstadt, Germany). Data acquisition was performed with the Thermo Xcalibur® software [(version 3.2.63), Thermo Scientific, Waltham, MA], Progenesis QI® software (Waters Corporation, Milford, MA) and Lipidhunter2 software [55].

### Acylcarnitine profiling

Acylcarnitines were measured in 15% quadriceps homogenate prepared in DPBS (Gibco 14190) using a BeadBeater system. Ten microlitres was collected per sample, and 100 μL of acetonitrile was added, followed by the addition of 100 μL of internal standard ([8,8,8-^2^H_3_]-octanoyl-L-carnitine and [10,10,10-^2^H_3_]-decanoyl-L-carnitine). Samples were then centrifuged at 20,000*g* for 10 min, collected into glass vials and analysed as previously described [56].

### Mitochondrial isolation and respiration

Mitochondria were isolated as previously described with adaptations from fresh quadriceps [16] and liver [46]. For the quadriceps, the tissue was dissected on ice to remove the excess of fat, finely minced in isolation buffer (220 mM mannitol, 70 mM sucrose, 5 mM 2-[Tris(hydroxymethyl)-methylamino]-ethanesulfonic acid (TES), 0.1 mM EGTA, pH 7.3) supplemented with 0.2 mg/mL proteinase (Sigma P8038) and incubated for 5 min. Subsequently, BSA was added to a final concentration of 0.5 mg/mL and homogenized using a Potter system (VWR, VOS power basic). Samples were centrifuged at 800*g* for 10 min, the pellet was discarded and the remaining supernatant was centrifuged at 7200*g* for 10 min. The pellet was then resuspended in 5 mL of isolation buffer and centrifuged one more time at 7200*g* for 10 min. The remaining pellet was resuspended in mitochondrial buffer (200 mM sucrose, 10 mM Tris, pH 7.4), and protein content was determined via the Pierce BCA Protein Assay Kit (Thermo Fisher 23225).

Oxygen consumption rates were measured in MiR05 buffer (respiration buffer) containing 110 mM sucrose, 60 mM potassium lactobionate, 20 mM taurine, 20 mM HEPES, 0.5 mM EGTA, 10 mM KH_2_PO_4_, 3 mM MgCl_2_, 1 mg/mL bovine serum albumin (BSA), pH 7.1 [57] at 37 °C using a two-channel high-resolution Oroboros oxygraph-2k (Oroboros, Innsbruck, Austria). Different substrate combinations were used, namely 2 mM pyruvate and 2 mM malate (with or without 5 mM glutamate) or 25 μM palmitoyl-CoA and 2 mM L-carnitine, all in the presence of 2 mM malate. Maximal ADP-stimulated respiration (state 3) was measured in the presence of 1.5 U/mL hexokinase, 10 mM glucose and 1 mM ATP. State 4 rates were measured after the addition of 1.25 μM carboxyatractyloside (CAT).

### Proteomics

Fifteen per cent (w/v) quadriceps homogenates were prepared in DPBS (Gibco 14190) with a BeadBeater system (Precellys® Evolution, Bertin Technologies). Samples were centrifuged at 15,000*g* for 5 min, supernatants were collected and cOmplete proteinase inhibitor cocktail was added (1:25, Merck 11836145001). Protein concentrations were measured with the Pierce BCA Protein Assay Kit (Thermo Fisher 23225) Protein levels of the proteins related to the mitochondrial and glycose pathways were quantified from the skeletal muscle homogenates using targeted proteomics [25]. Briefly, in-gel digestion was performed on 50 μg total protein for the skeletal muscle homogenates using trypsin (1:100 g/g sequencing grade modified trypsin V5111; Promega) after reduction with 10 mmol/L dithiothreitol and alkylation with 55 mmol/L iodoacetamide, followed by solid-phase extraction (SPE C18-Aq 50 mg/1 mL, Gracepure, Thermo Fisher Scientific) for sample clean-up. In-gel digestion with trypsin, LC-MS analysis and data analysis have been performed according to Wolters et al. [25]. Briefly, liquid chromatography (LC) on a nano-ultra high-performance liquid chromatography (UHPLC) system (Ultimate UHPLC focused; Dionex, Thermo Fisher Scientific) was performed to separate the peptides using a nanocolumn (Acclaim PepMap100 C18, 75 μm × 500 mm 2 μm, 100 Å) with a linear gradient from 3 to 60% v/v acetonitrile plus 0.1% v/v formic acid in 110 min at a flow rate of 200 nL/min. The target peptides were analysed by a triple quadrupole mass spectrometer (MS) equipped with a nano-electrospray ion source (TSQ Vantage; Thermo Scientific). For the LC-MS measurements, an amount of the digested peptides equivalent to a total protein amount of 1 μg total protein starting material was injected together with 0.2 (low abundant subset targets) or 1 (high abundant subset targets) ng isotopically labelled concatemer-derived standard peptides (QconCAT technology, PolyQuant GmbH Germany, containing ^13^C-labelled arginines and lysines), plus 5 pmol GAPDH and 1 fmol SLC25A20 isotopically labelled standard peptides (PEPotec grade 2, Thermo Scientific, containing ^13^C^15^N-labelled arginines and lysines). The MS traces were manually curated using the Skyline software [58] prior to the integration of the peak areas for quantification. The sum of all transition peak areas for the endogenous peptide and isotopically labelled peptide standard was used to calculate the ratio between the endogenous and standard peptides. The concentrations of the endogenous peptides were calculated from the known concentration of the standard and expressed in femtomoles per microgram of total protein. For proteins with more than one detected peptide, values were averaged in order to obtain an estimation for the total protein concentration. For the newly developed peptides spanning proteins in glucose metabolism (details in Additional file 4), the glucose transporters (GLUT family) could not be detected endogenously, probably due to a limited extraction efficiency of plasma-membrane proteins.

### Enzyme activities

Citrate synthase (CS), hexokinase and pyruvate kinase activities were measured in homogenates prepared in the same way as described above for proteomics assay. Citrate synthase activity was measured as previously described [59] in homogenates and mitochondrial preparations. Briefly, samples were incubated in an experimental buffer containing 100 mM Tris-HCl, 5 mM triethanolamine-HCl, 0.5 mM oxaloacetate, 0.1 mM dithionitrobenzoate and 0.1% Triton-X, pH 8.1. The final protein concentration of samples was between 25 and 40 μg/mL. Reactions were initiated by the addition of 0.5 mM acetyl-CoA (final concentration) and thionitrobenzoate (oxidized product) production was followed by absorbance measurements at 412 nm and 37 °C for 5 min. The ratio between CS activity in the homogenate and the mitochondria gives the ratio between mitochondria protein per total tissue protein; thus, it reflects the enrichment of mitochondrial preparations. Consequently, this ratio can be used to express results per total tissue capacity [16].

Hexokinase and pyruvate kinase activities were carried out using NAD(P)H-linked assays at 37 °C in a Synergy H4 plate reader (BioTek™) at 340 nm for 6 min as previously described [60]. Briefly, for both enzyme measurements, the assay buffer contained 100 mM Tris-HCl, 15 mM NaCl, 0.5 mM CaCl_2_, 140 mM KCl and 5 mM potassium phosphate buffer (pH 7.0). For hexokinase activity measurements, 1.2 mM NADP^+^ (Sigma N0505), 10 mM Glucose (Merck 1.083420), 1.8 U/mL glucose-6-phosphate dehydrogenase (Sigma G7877), 10.5 mM MgSO_4_ and 10 mM ATP (Sigma A2383) as start reagent were used. The buffer for pyruvate kinase measurements contained 0.15 mM NADH (Sigma N8129), 1 mM ADP (Sigma A5285), 1 mM fructose 1,6-bisphosphate (Sigma F0752), 60 U/mL L-lactate dehydrogenase (Sigma L2500) and 2 mM phosphoenolpyruvate (Sigma P7252) was used as start reagent. Four dilutions were performed per sample (0.4–4.5 mg protein/mL) to check for linearity over time and samples were further diluted 50× for pyruvate kinase and 10× for hexokinase in the experimental buffer for measurements.

Carnitine palmitoyltransferase (CPT) activity was measured using mitochondrial enriched suspensions. The experimental buffer contained 25 mM Tris-HCl, 2 mM EDTA, 150 mM KCl, 5 mM KCN, 1 mg/mL BSA, 4.5 mM reduced glutathione and 0.02 mM potassium phosphate buffer (pH 7.0); 50 μM palmitoyl-CoA (Sigma P9716) and 2 mM L-carnitine (Sigma C0158) were used as substrates and the mixture was incubated for 10 min at 37 °C. Two sample dilutions were used per animal (8 or 16 ng protein/mL), and samples were collected and quenched in acetonitrile at 0, 5 or 10 min (adapted from [61]). The concentration of palmitoyl-carnitine produced was measured according to the acylcarnitine profiling described above after the addition of internal standard.

### Western blots

For Western blot analyses, frozen quadriceps samples were used to prepare 7.5% homogenates (w/v) in 0.1% NP-40 buffer (0.4 M NaCl) in the presence of 1 tablet of cOmplete protease inhibitor cocktail (Merck, 11836145001), 0.5 mL phosphatase inhibitor cocktail 2 (Sigma-Aldrich P5726), 0.5 mL phosphatase inhibitor cocktail 3 (Sigma-Aldrich P0044) and 1 mM dithiothreitol (DTT). Samples were homogenized in a BeadBeater, mixed with SDS loading buffer and heated at 95 °C for 5 min. SDS-PAGE was used to separate proteins in 8% acrylamide gels. Ninety to 150 V was used to separate the proteins using a MiniPROTEAN Tetra Vertical Electrophoresis Cell system (Cat. No. 1658029FC; Bio-Rad) in the presence of running buffer (0.2 M glycine, 25 mM Tris and 0.1% SDS). Transfer was performed using polyvinylidene difluoride (PVDF) in blotting buffer (0.1 M glycine, 50 mM Tris, 0.01% SDS and 10% methanol, pH 8.3) at 45 V for 1 h 45 min. The membranes were blocked in 5% BSA in TBST for 1 h, and primary antibodies were incubated overnight at 4 °C. Subsequently, the membranes were washed three times with TBST and incubated with HRP-coupled secondary antibody (goat anti-mouse or goat anti-rabbit) for 2 h followed by wash before detection. Pierce ECL substrate (Thermo Fisher Scientific, 32209) was used for detection using Image Quant LAS4000 Mini (GE Healthcare) [62]. Antibodies used are as follows: actin (Millipore MAB1501, 1:100,000), ACC (Cell Signalling, 3676, 1:1000), pACC(S79) (Cell Signalling, 3661, 1:2000), MCD (Abcam, ab95945, 1:1000), GAPDH (Abcam, ab37187, 1:20,000) and PGC1α (Abcam, ab54481, 1:1000). Original blots can be found in Additional File 7.

### TCA cycle intermediates

Fifteen per cent quadriceps homogenates (w/v) were prepared as described for the acylcarnitine profiling. Samples were subsequently sonicated at 40% amplitude for 30 s. Samples were diluted 3 times, and 1 mL was loaded in glass tubes and 150 μL of internal standard (1:2 nor-leucine/4-phenylbutyrate) was added. One millilitre of methanol was added, followed by the addition of 2 mL ice-cold chloroform. Samples were vortexed for 30 min at 4 °C and centrifuged at 3000 rpm for 10 min. The upper aqueous phase was collected and evaporated under N_2_ stream at 37 °C. Dried polar metabolites were resuspended in 40 μL 2% methoxyamine in pyridine and incubated at 37 °C for further 90 min. Subsequently, 60 μL of MTBSTFA + 1% TBDMCS silylation reagent (Sigma 375934) was added and derivatization proceeded at 55 °C for 1 h. A standard curve for TCA metabolites was included for all procedures. Samples were transferred to glass vials prior to analysis by gas chromatography coupled to mass spectrometry (GC-MS). The analysis proceeded according to the specifications described in Evers et al. [63].

### Computational modelling

The computational model of mouse mitochondrial β-oxidation previously described [29] was parameterized on an individual mouse basis. This was based on the quantitative proteomics data obtained for this study, thereby yielding a set of results for each experimental group (Additional file 8). The rate through the CPT1 reaction with palmitoyl-CoA as substrate (vcpt1c16 in the computational model of the mitochondrial β-oxidation) was used as the β-oxidation flux in each simulation. Flux control coefficients (FCC) were calculated as previously described [19]. The definition of the parameter and the estimation used for calculations can be found in Eqs. 2 and 3, respectively. It corresponds to which extent a certain enzyme controls the flux (J) through a pathway (∆V_max_ = V_max_∙10^−6^ μmol ∙ min^−1^∙mg protein^−1^). Summation theorem was checked [31]. A full description of the modelling strategy and the script can be found in Additional file 6 [16, 26, 29, 64, 65] and the full code in Additional file 9.

2 $$ {FCC}_{enzyme}^J=\frac{\partial J/J}{\partial {V}_{max}/{V}_{max}} $$

3 $$ {FCC}_{enzyme}^J\approx \frac{\Delta J}{\Delta {V}_{max}}\cdot \frac{V_{max}}{J} $$

### Statistical analysis

Data were visualized using the GraphPad Prism software (GraphPad Software Inc., version 8.0, 2018) and analysed with either the same software or IBM SPSS Statistics (IBM Corp., version 25.0, 2017). Body weight and glucose and insulin time courses were analysed with 3-way ANOVA (age, diet and time). Delta fat mass, HOMA-IR, insulin action, plasma TGs and NEFAs were analysed by 2-way ANOVA (HOMA-IR and TGs after log_2_ transformation) followed by Tukey’s multiple comparisons. Energy expenditure was adjusted for total body weight with ANCOVA according to the guidelines of the National Mouse Metabolic Phenotyping Centers. RER and adjusted energy expenditure were analysed with 3-way ANOVA (diet, age and light/dark cycle), followed by Holm-Sidak’s multiple comparisons test. Lipidomics data were transformed by mean centring and multiple Student’s t-tests were performed on data (comparing the effect of the diets on matched age groups) after log_2_ transformation. Correction for multiple comparisons used the false discovery rate (FDR) approach (Q-value = 5%). A significant change was defined as having a q value < 0.05 and a fold change ≤ 0.66 or ≥ 1.5. Individual lipid species and PC/PE ratio were analysed with 2-way ANOVA followed by Tukey’s multiple comparison analysis. Association between MISI and lipid species was assessed by Pearson correlation coefficients. Remaining comparisons (Western blots, enzyme activity, O_2_ consumption and protein data) were performed by 2-way ANOVA followed by Tukey’s multiple comparisons. The effect of each factor based on the 2-way ANOVA (p_age_ and p_diet_), as well as the interaction between them (p_age × diet_), was calculated for each comparison.

## Supplementary Information

**Additional file 1.** Supplemental figures.

**Additional file 2.** Supplemental tables.

**Additional file 3.** Lipidomics dataset.

**Additional file 4.** Proteomics dataset.

**Additional file 5.** Average proteomics.

**Additional file 6.** Modelling strategy.

**Additional file 7.** Original blots.

**Additional file 8.** V_max_ values.

**Additional file 9.** Computational model script.

**Additional file 10.** All supporting data values.

## Data Availability

All data generated or analysed during this study are included in this published article and its supplementary information files.
