# Supplementary material for: Age-related susceptibility to insulin resistance arises from a combination of CPT1B decline and lipid overload
Source: BMC Biol. 2021 Jul 30;19:154. doi: 10.1186/s12915-021-01082-5 (PMC8323306; doi:10.1186/s12915-021-01082-5)
Supplement: Supplementary file 1 — Additional file 1. Supplemental figures. [file 12915_2021_1082_MOESM1_ESM.pdf]

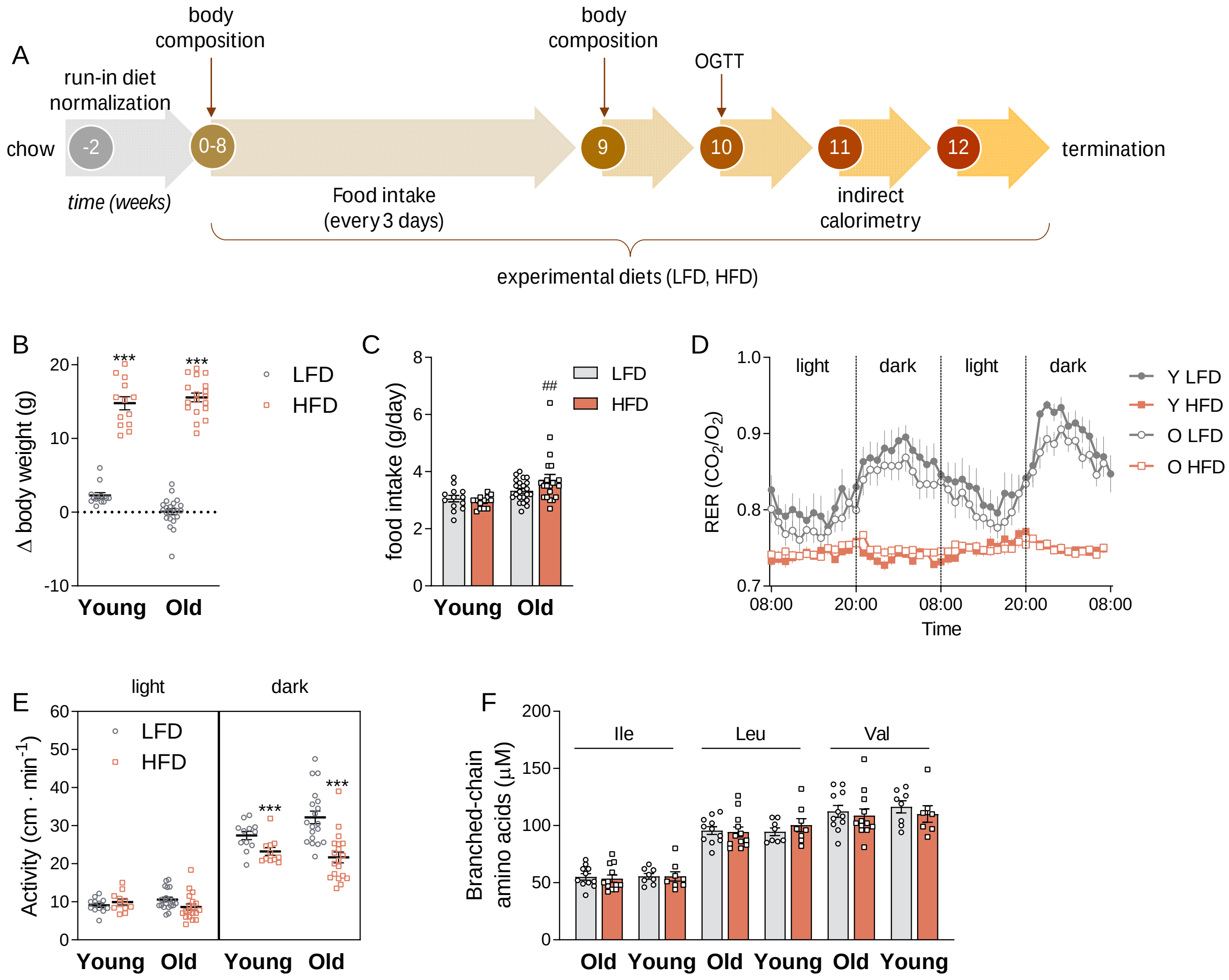
A
body composition
run-in diet normalization
chow
-2
0-8
9
10
11
12
OGTT
termination
time (weeks)
Food intake (every 3 days)
indirect calorimetry
experimental diets (LFD, HFD)
B
Δ body weight (g)
***
LFD
HFD
Young
Old
C
food intake (g/day)
##
D
RER (CO2/O2)
light
dark
Y LFD
Y HFD
O LFD
O HFD
08:00
20:00
Time
E
Activity (cm · min-1)
F
Branched-chain amino acids (μM)
Ile
Leu
Val

Supp. Fig. 1

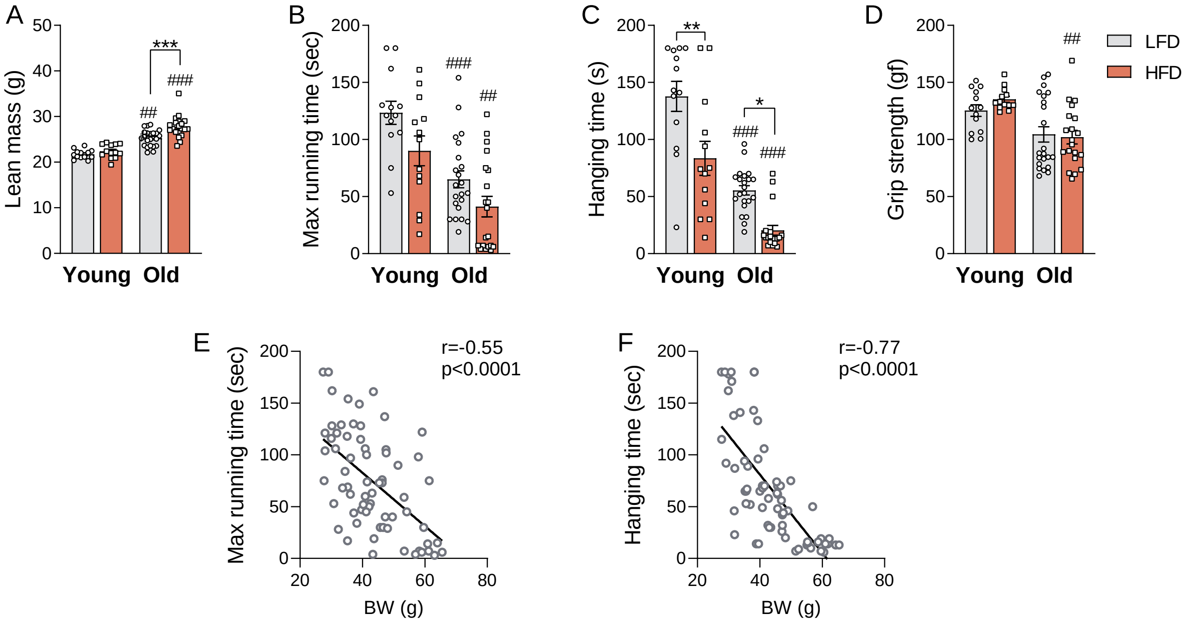
A
Lean mass (g)
50
40
30
20
10
0
Young
Old
***
##
###
B
Max running time (sec)
200
150
100
50
0
Young
Old
###
##
C
Hanging time (s)
200
150
100
50
0
Young
Old
**
*
###
###
D
Grip strength (gf)
200
150
100
50
0
Young
Old
##
LFD
HFD
E
Max running time (sec)
200
150
100
50
0
20
40
60
80
BW (g)
r=-0.55
p<0.0001
F
Hanging time (sec)
200
150
100
50
0
20
40
60
80
BW (g)
r=-0.77
p<0.0001

Supp. Fig. 2

A

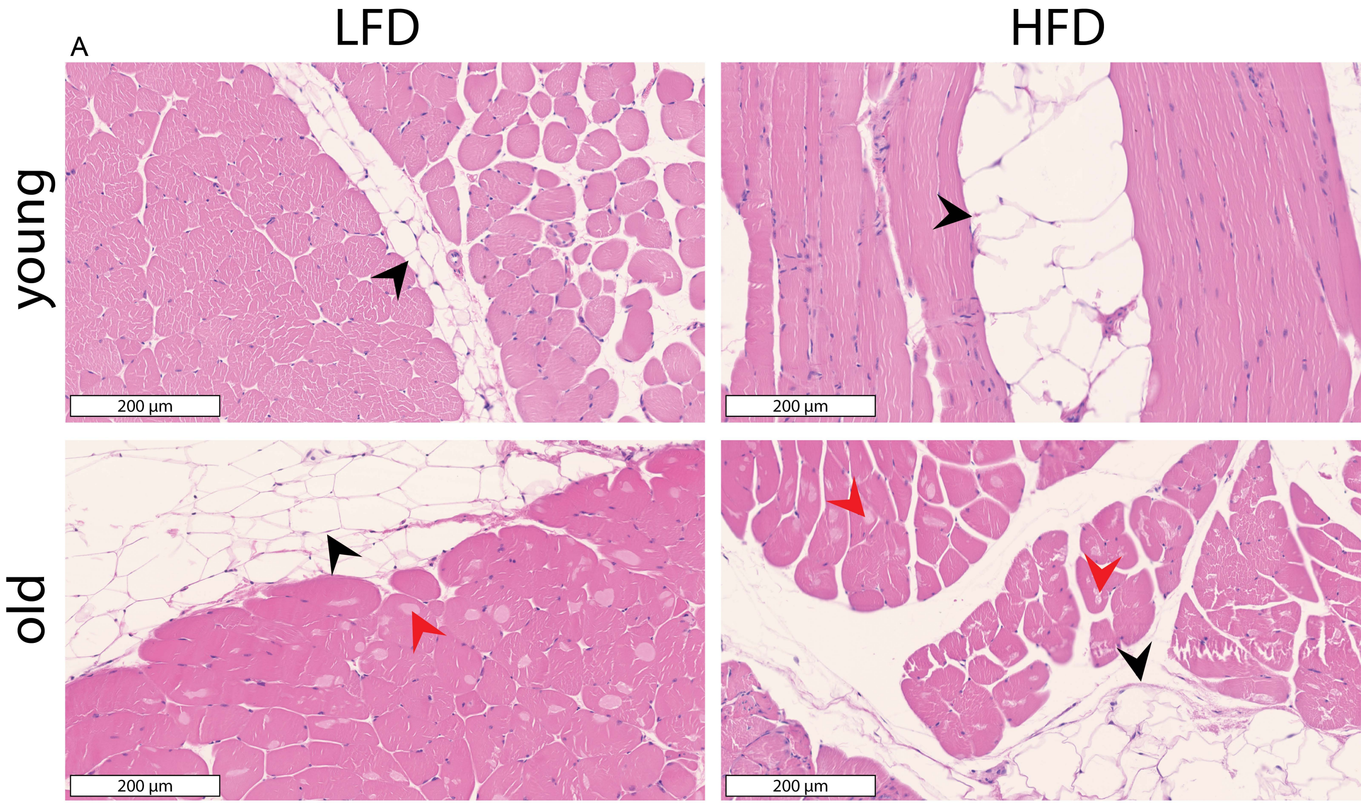

B

Group	Vacuolization (mean)	Fat infiltration (mean)
Young LFD	0	1
Young HFD	0	2
Old LFD	2	2
Old HFD	3	3

C

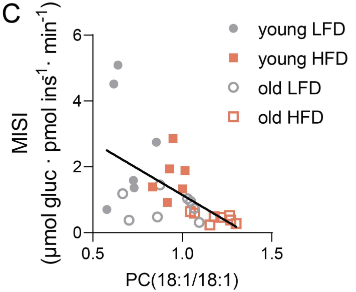

Supp. Fig. 3

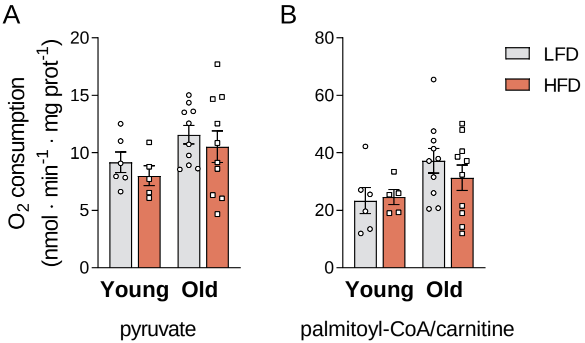
A
B
O_2 consumption
(nmol · min^{-1} · mg prot^{-1})
20
15
10
5
0
80
60
40
20
0
LFD
HFD
Young Old
Young Old
pyruvate
palmitoyl-CoA/carnitine

Supp. Fig. 4

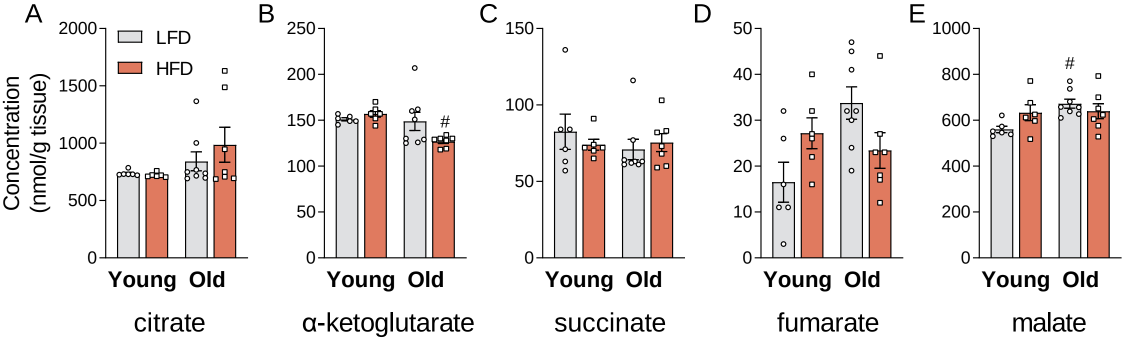
A
B
C
D
E
LFD
HFD
Concentration (nmol/g tissue)
2000
1500
1000
500
0
250
200
150
100
50
0
150
100
50
0
50
40
30
20
10
0
1000
800
600
400
200
0
#
#
Young Old
Young Old
Young Old
Young Old
Young Old
citrate
α-ketoglutarate
succinate
fumarate
malate

Supp. Fig. 5

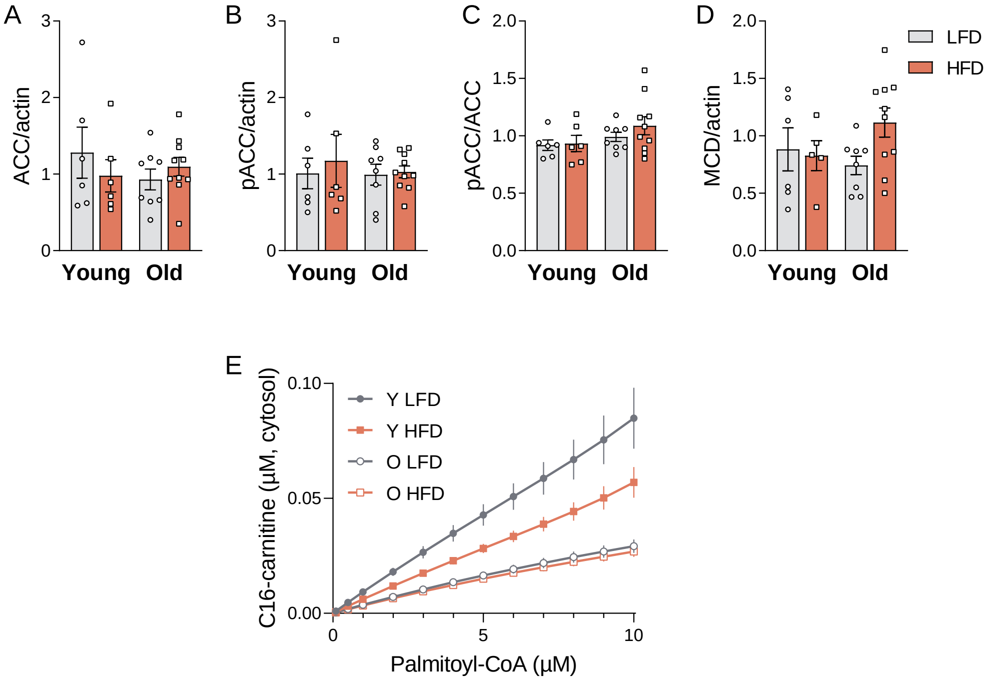
A
B
C
D
ACC/actin
pACC/actin
pACC/ACC
MCD/actin
Young
Old
LFD
HFD
E
C16-carnitine (µM, cytosol)
Palmitoyl-CoA (µM)
Y LFD
Y HFD
O LFD
O HFD

Supp. Fig. 6

Supplemental Figure - Legends

Supplemental Figure 1: A: Schematic design of animal experiments. B: Change in body weight for both age groups after 8 weeks on either a LFD or HFD. C: Average food intake per day from several measurements over the course of 8 weeks. D: 48h time course for respiratory exchange ratios (RER) in both light/dark phases following 10 weeks for each age/diet group. E: Activity of animals (cm · min^{-1}) measured in metabolic cages with the use of lasers in parallel with indirect calorimetry. F: Plasma branched-chain amino acids measured at termination blood spots for all age/diet groups. Data are shown as mean ± SEM. n=13-22 per group, ***p<0.001 (LFD vs HFD, matched age), Two-Way-ANOVA followed by Tukey's post hoc test.

Supplemental figure 2: A: lean mass, B: maximal running time in seconds, C: hanging time in seconds, D: grip strength (gf). Spearman correlations between body weight (BW) in grams and either maximal running time (E) or hanging time (F). Data are shown as mean ± SEM. n=13-22 per group, *p<0.05, **p<0.01,***p<0.001 (LFD vs HFD, matched age), $^{\#\#}$p<0.01, $^{\#\#\#}$p<0.001 (old vs young, matched diet), Two-Way-ANOVA followed by Tukey's post hoc test.

Supplemental figure 3: A: H&E staining of quadriceps samples. Representative images per experimental group. Black arrows represent fat infiltration and red arrows show vacuolization. B: quantification of fat infiltration and vacuolization per group (n=3-4). C: Linear regression between phosphatidylcholine PC(18:1/18:1) and MISI (Pearson r = -0.56, p = 0.002).

Supplemental figure 4: Maximal ADP-stimulated O_2 consumption in liver corrected for total tissue protein. A: pyruvate and B: palmitoyl-CoA and carnitine (D) were used as substrates, all in the presence of malate.

Supplemental Figure 5: Absolute quantification of TCA cycle intermediates in nmol/g tissue. A: citrate, B: α-ketoglutarate, C: succinate, D: fumarate, E: malate. Data are shown as mean ± SEM. n=6-9 per group, $^{\#}$p<0.05 (old vs young, matched diet), Two-Way-ANOVA followed by Tukey's post hoc test.

Supplemental figure 6: A-D: Total ACC, pACC (S79), pACC/ACC and MCD levels in quadriceps homogenates normalized by actin levels. E: predicted cytosolic C16-carnitine concentrations, n=5-10 per group.
