# Supplementary material for: Age-related susceptibility to insulin resistance arises from a combination of CPT1B decline and lipid overload
Source: BMC Biol. 2021 Jul 30;19:154. doi: 10.1186/s12915-021-01082-5 (PMC8323306; doi:10.1186/s12915-021-01082-5)
Supplement: Supplementary file 2 — Additional file 2. Supplemental tables. [file 12915_2021_1082_MOESM2_ESM.pdf]

Supplemental Material

Supplemental table 1: Diet composition (D17041407, -09)

Product #	D17041407		D17041409	
	Low Fat (20 kcal %)		High Fat (60 kcal %)	
**Ingredient**	(g)		(g)	
**Casein**	220		220	
**Cystine**	3		3	
**Corn Starch**	368		67	
**Maltodextrin 10**	122.2		22.3	
**Sucrose**	107.078		107.078	
**Cellulose**	50		50	
**Soybean Oil**	90		90	
**Lard**	0		178.4	
**t-butylhydroquinone**	0.014		0.014	
**Mineral Mix S10022C**	3.5		3.5	
**Calcium Carbonate**	12.495		12.495	
**Potassium Citrate, 1 H20**	1.773		1.773	
**Potassium Phosphate, monobasic**	7.763		7.763	
**Sodium Chloride**	2.59		2.59	
**Vitamin Mix V10037**	10		10	
**Choline Bitartrate**	2.5		2.5	
**FD&C Yellow Dye #5**	0.05		0	
**FD&C Red Dye #40**	0		0.025	
**FD&C Blue Dye #1**	0		0.025	
**Total**	1000.96		778.463	
**kcal/gm**	4		5.2	
	gram	gram%	gram	gram%
**Protein**	197	20	197	25
**Carbohydrate**	607	61	206	27
**Fat**	90	9	268	34
**Fiber**	50	5	50	6
	kcal	kcal%	kcal	kcal%
**Protein**	786	20	786	20
**Carbohydrate**	2429	60	826	20
**Fat**	810	20	2416	60
**Total**	4026	100	4028	100
	gram	gram%	gram	gram%
**Calcium**	5	0.5	5	0.64
**Phosphorus**	3.53	0.35	3.53	0.45
**Potassium**	3.6	0.36	3.6	0.46
	Typical Amino Acids (g)			
Essential Amino Acids	per kg diet	per 1000 kcal	per kg diet	per 1000 kcal

**Histidine**	4.95	1.23	6.36	1.23
**Isoleucine**	8.24	2.05	10.6	2.05
**Leucine**	17.25	4.29	22.18	4.29
**Lysine**	14.29	3.55	18.37	3.55
**Methionine**	5.49	1.37	7.07	1.37
**Phenylalanine**	9.12	2.27	11.73	2.27
**Threonine**	7.8	1.94	10.03	1.94
**Tryptophan**	2.31	0.57	2.97	0.57
**Valine**	10.11	2.51	13	2.51
Non-Essential Amino Acids				
**Alanine**	5.49	1.37	7.07	1.37
**Arginine**	6.48	1.61	8.34	1.61
**Asparagine**	7.69	1.91	9.89	1.91
**Aspartate**	5.49	1.37	7.07	1.37
**Cystine**	4.32	1.07	4.7	1.07
**Glutamine**	18.68	4.65	24.02	4.64
**Glutamate**	22.75	5.66	29.25	5.65
**Glycine**	3.3	0.82	4.24	0.82
**Proline**	19.34	4.81	24.87	4.81
**Serine**	10.88	2.71	13.99	2.7
**Tyrosine**	9.89	2.46	12.72	2.46

Supplemental table 2: Lipids that are changed by a HFD in either old or young mice.

Lipid name	Detailed composition	Significant result LFD vs HFD	Fold change	q-value
**TG(54:1)**	**TG(18:0_18:0_18:1)**	old	7.7	0.0017
**TG(41:1)**		old	6.0	0.0258
**23OH-C20:0-carnitine**		old	5.7	0.0035
**TG(52:0)**	**TG(16:0_18:0_18:0)**	old	5.5	0.0017
**C18:0-carnitine**		old	4.9	0.0005
**DG(36:1)**	**DG(18:0_18:1)**	old	4.3	0.0025
**TG(50:0)**	**TG(16:0_16:0_18:0)**	old	4.1	0.0022
**TG(53:1)**	**TG(17:0_18:0_18:1)**	old	3.9	0.0137
**C16:0-carnitine**		old	3.8	0.0055
**CL(72:8)**	**CL(18:2/18:2/18:2/18:2)**	old	3.5	0.0129
**TG(54:2)**	**TG(18:0_18:1_18:1)**	old	3.4	0.0147
**3OH-C18:1-carnitine**		old	3.2	0.0297
**TG(51:1)**	**TG(16:0_17:0_18:1)**	old	2.9	0.0317
**PC(40:4)**	**PC(18:0_22:4)**	old	2.8	0.0033
**TG(51:0)**	**TG(16:0_17:0_18:0)**	old	2.7	0.0156
**TG(59:2)**		old	2.7	0.0499
**DG(38:4)**	**DG(16:0_22:4)**	old	2.7	0.0054
**C14-carnitine**		old	2.6	0.0352
**PC(38:4)**	**PE(16:0_22:4)**	old	2.6	0.0352
**TG(42:0)**	**TG(12:0_14:0_16:0)**	old	2.3	0.0240
**PC(37:4)**	**PC(17:0_20:4)**	old	2.2	0.0005
**PC(37:3)**		old	2.2	0.0189
**PEp(40:5)/PEa(40:6)**		young/old	2.2/1.9	0.0438/0.0129
**PEa(40:5)/PEp(40:4)**		young/old	2.1/2.2	0.0307/0.0021
**SM(d36:2)**		young/old	2.1/2.2	0.0264/0.0005
**TG(49:0)**	**TG(16:0_16:0_17:0)**	old	2.1	0.0475
**SM(d34:2)**		old	2.1	0.0119
**PC(38:2)**		old	2.1	0.0033
**PC(38:3)**	**PC(18:0_20:3)**	old	2.1	0.0116
**DG(32:0)**	**DG(16:0/16:0)**	old	2.0	0.0035
**PC(39:5)**		young/old	1.9/2.0	0.0198/0.0033
**Cer(d36:1)**	**Cer(d18:1/18:0)**	old	1.9	0.0033
**DG(34:0)**	**DG(16:0_18:0)**	old	1.9	0.0005
**DG(35:2)**		old	1.9	0.0309
**PC(38:4)**	**PC(16:0_22:4)**	young/old	1.8/2.6	0.0198/0.0001
**PC(36:2)**	**PC(18:0_18:2)**	old	1.8	0.0071
**PC(35:2)**	**PC(17:0_18:2)**	old	1.8	0.0021
**PCa(38:4)/PCp(38:3)**		old	1.8	0.0129
**PC(38:4)**	**PC(18:0_20:4)**	old	1.8	0.0035
**PC(36:3)**	**PC(16:0_20:3)**	old	1.8	0.0055
**LPE(18:0)**		old	1.8	0.0054

**LPC(20:4)**		old	1.8	0.0147
**SM(d40:2)**		old	1.8	0.0317
**LPC(22:5)**		old	1.7	0.0335
**Cer(d40:1)**	**Cer(d18:1/22:0)**	old	1.7	0.0156
**LPC(18:0)**		old	1.7	0.0182
**PC(33:0)**		old	1.7	0.0116
**PC(36:4)**	**PC(16:0_20:4)**	old	1.7	0.0054
**PC(35:1)**		young/old	1.6/1.8	0.0198/0.0035
**PC(38:5)**	**PC(16:0_22:5) major**	old	1.6	0.0220
**PEp(38:4)/PEa(38:5)**		old	1.6	0.0054
**PEp(38:4)/PEa(38:5)**		old	1.6	0.0303
**PC(36:1)**	**PC(18:0_18:1)**	old	1.6	0.0198
**DG(38:6)**	**DG(16:0_22:6)**	old	1.6	0.0208
**PEp(38:5(OH))**		young/old	1.5/1.7	0.0307/0.0035
**SM(d35:1)**		old	1.5	0.0116
**DG(38:4)**	**DG(18:0_20:4)**	old	1.5	0.0261
**PC(34:2)**	**PC(16:0_18:2) major**	old	1.5	0.0129
**DG(33:2)**		old	0.6	0.0137
**PC(32:2)**	**PC(14:0_18:2)**	young	0.5	0.0209
**PC(34:4)**		young/old	0.4/0.6	0.0198/0.0147
**TG(59: 3)**		old	0.4	0.0178

Supplemental table 3: Acylcarnitine composition in quadriceps (nmol * g tissue $^{-1}$)

	YOUNG				OLD			
	LFD		HFD		LFD		HFD	
**Species**	**Mean**	**SEM**	**Mean**	**SEM**	**Mean**	**SEM**	**Mean**	**SEM**
**total**	129.8	9.4	145.7	3.6	165.7	20.1	162.6	6.6
**C0**	56.5	4.2	67.6	2.3	71.4	8.5	73.0	3.4
**C2***	57.0	5.2	63.8	4.4	81.1	10.7	72.4	4.7
**C3**	0.47	0.05	0.49	0.04	0.59	0.08	0.57	0.04
**C4**	0.85	0.11	0.86	0.05	0.89	0.11	1.02	0.06
**C5**	0.12	0.01	0.16	0.01	0.16	0.02	0.23	0.05
**C6**	0.24	0.03	0.29	0.01	0.25	0.03	0.31	0.01
**C8**	0.14	0.01	0.16	0.02	0.13	0.01	0.15	0.01
**C10:1**	0.03	0.01	0.02	0.00	0.02	0.01	0.02	0.00
**C10**	0.05	0.01	0.04	0.01	0.03	0.00	0.04	0.01
**C12:1**	0.03	0.01	0.02	0.00	0.02	0.01	0.02	0.00
**C12**	0.09	0.02	0.05	0.02	0.05	0.01	0.06	0.01
**C14:1**	0.28	0.07	0.17	0.04	0.21	0.04	0.22	0.05
**C14**	0.83	0.14	0.69	0.16	0.52	0.09	0.69	0.12
**C16:1**	0.84	0.16	0.47	0.11	0.66	0.14	0.59	0.14
**C16**	3.92	0.70	3.51	0.97	2.35	0.46	4.00	0.73
**C18:2**	1.05	0.23	0.54	0.13	0.83	0.18	0.81	0.21
**C18:1**	1.92	0.36	1.46	0.38	1.83	0.35	2.33	0.66
**C18**	1.03	0.18	1.22	0.30	0.78	0.15	**1.49**$^{\#}$	0.23
**C12OH**	0.07	0.01	0.10	0.01	0.10	0.04	0.14	0.02
**C14OH**	0.08	0.01	0.10	0.01	0.06	0.01	0.10	0.01
**C16OH**$^{\$}$	0.26	0.03	0.35	0.05	0.19	0.03	**0.36**$^{\&}$	0.03
**C18:1OH**$^{\$}$	0.25	0.04	0.34	0.05	0.26	0.04	**0.43**$^{\#}$	0.05
**C18OH**$^{\$}$	0.09	0.01	0.14	0.03	0.08	0.01	**0.17**$^{+}$	0.01

*p<0.05 for age and $p<0.01 for diet, 2-way ANOVA. #p<0.05 for LFD vs HFD, old, &p<0.01 for LFD vs HFD, old, and +p<0.001 for LFD vs HFD, old.
