# Supplementary figures and images for: Age-related susceptibility to insulin resistance arises from a combination of CPT1B decline and lipid overload
Source: BMC Biol. 2021 Jul 30;19:154. doi: 10.1186/s12915-021-01082-5 (PMC8323306; doi:10.1186/s12915-021-01082-5)

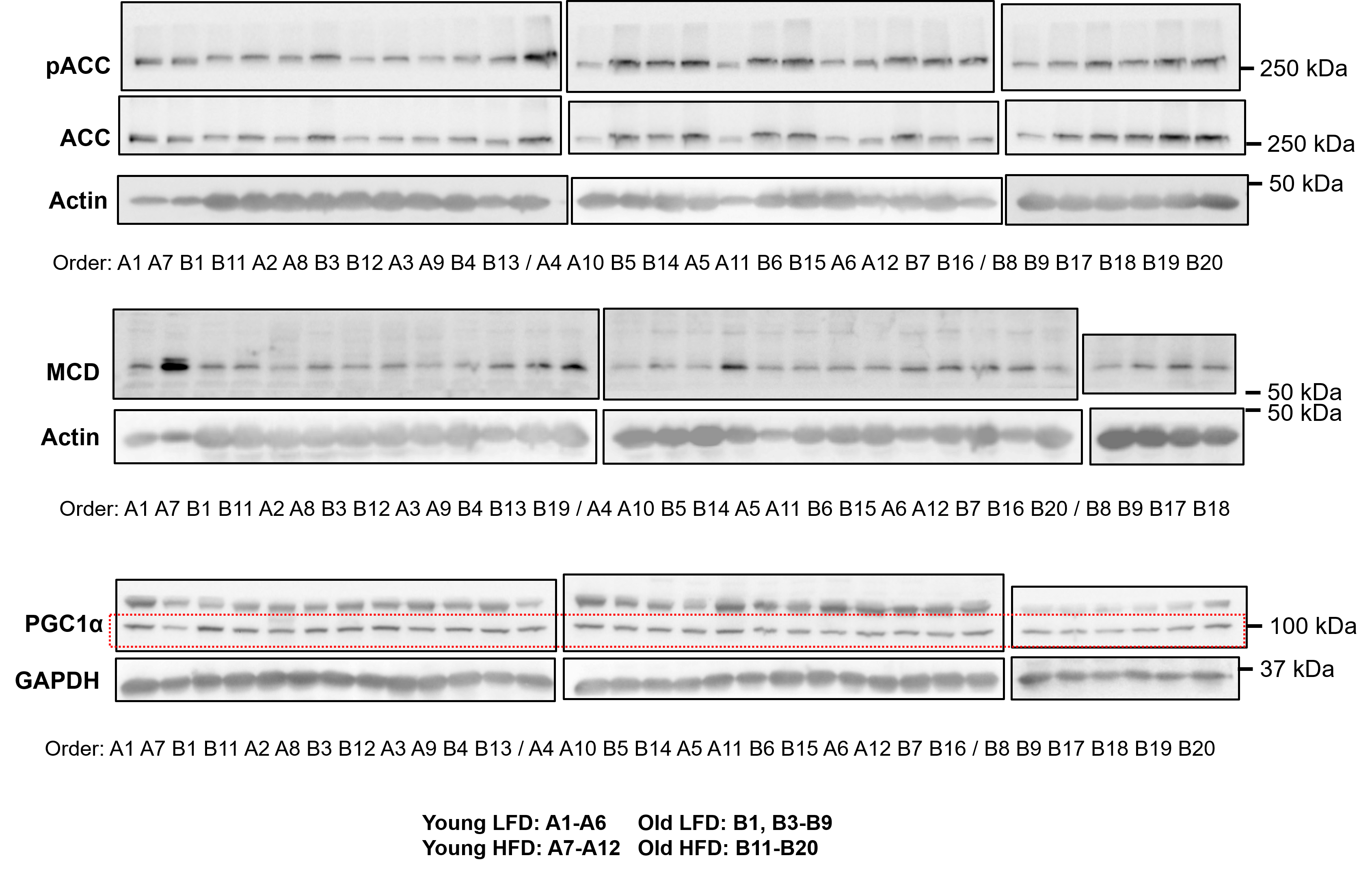

Supplement: Supplementary file 7 — Additional file 7. Original blots. [file 12915_2021_1082_MOESM7_ESM.tif]
