# Supplementary material for: Age-related susceptibility to insulin resistance arises from a combination of CPT1B decline and lipid overload
Source: BMC Biol. 2021 Jul 30;19:154. doi: 10.1186/s12915-021-01082-5 (PMC8323306; doi:10.1186/s12915-021-01082-5)
Supplement: Supplementary file 9 — Additional file 9. Computational model script. [file 12915_2021_1082_MOESM9_ESM.pdf]

Kinetic model

Definitions of the various functions

CPT1[sf_, V_, Kms1_, Kms2_, Kmp1_, Kmp2_, Ki1_, Keq_, S1_, S2_, P1_, P2_, I1_, n_] :=

$$\frac{sf * V * \left(\frac{S1*S2}{Kms1*Kms2} - \frac{P1*P2}{Kms1*Kms2*Keq}\right)}{\left(1 + \frac{S1}{Kms1} + \frac{P1}{Kmp1} + \left(\frac{I1}{Ki1}\right)^{n}\right) * \left(1 + \frac{S2}{Kms2} + \frac{P2}{Kmp2}\right)}$$

CACT[Vf_, Vr_, Kms1_, Kms2_, Kmp1_, Kmp2_, Kis1_, Kip2_, Keq_, S1_, S2_, P1_, P2_] :=

$$\left(Vf * \left(S1 * S2 - \frac{P1 * P2}{Keq}\right)\right) \Big/ \left(S1 * S2 + Kms2 * S1 + Kms1 * S2 * \left(1 + \frac{P2}{Kip2}\right) + \frac{Vf}{Vr * Keq} * \left(Kmp2 * P1 * \left(1 + \frac{S1}{Kis1}\right) + P2 * (Kmp1 + P1)\right)\right)$$

CPT2[sf_, V_, Kms1_, Kms2_, Kms3_, Kms4_, Kms5_, Kms6_, Kms7_, Kms8_, Kmp1_, Kmp2_, Kmp3_, Kmp4_, Kmp5_, Kmp6_, Kmp7_, Kmp8_, Keq_, S1_, S2_, S3_, S4_, S5_, S6_, S7_, S8_, P1_, P2_, P3_, P4_, P5_, P6_, P7_, P8_] :=

$$\left(sf * V * \left(\frac{S1 * S8}{Kms1 * Kms8} - \frac{P1 * P8}{Kms1 * Kms8 * Keq}\right)\right) \Big/ \left(\left(1 + \frac{S1}{Kms1} + \frac{P1}{Kmp1} + \frac{S2}{Kms2} + \frac{P2}{Kmp2} + \frac{S3}{Kms3} + \frac{P3}{Kmp3} + \frac{S4}{Kms4} + \frac{P4}{Kmp4} + \frac{S5}{Kms5} + \frac{P5}{Kmp5} + \frac{S6}{Kms6} + \frac{P6}{Kmp6} + \frac{S7}{Kms7} + \frac{P7}{Kmp7}\right) * \left(1 + \frac{S8}{Kms8} + \frac{P8}{Kmp8}\right)\right)$$

VLCAD[sf_, V_, Kms1_, Kms2_, Kms3_, Kms4_, Kmp1_, Kmp2_, Kmp3_, Kmp4_, Keq_, S1_, S2_, S3_, S4_, P1_, P2_, P3_, P4_] :=

$$\frac{sf * V * \left(\frac{S1*(S4-P4)}{Kms1*Kms4} - \frac{P1*P4}{Kms1*Kms4*Keq}\right)}{\left(1 + \frac{S1}{Kms1} + \frac{P1}{Kmp1} + \frac{S2}{Kms2} + \frac{P2}{Kmp2} + \frac{S3}{Kms3} + \frac{P3}{Kmp3}\right) * \left(1 + \frac{(S4-P4)}{Kms4} + \frac{P4}{Kmp4}\right)}$$

LCAD[sf_, V_, Kms1_, Kms2_, Kms3_, Kms4_, Kms5_, Kms6_, Kmp1_, Kmp2_, Kmp3_, Kmp4_, Kmp5_, Kmp6_, Keq_, S1_, S2_, S3_, S4_, S5_, S6_, P1_, P2_, P3_, P4_, P5_, P6_] :=

$$\left(sf * V * \left(\frac{S1 * (S6 - P6)}{Kms1 * Kms6} - \frac{P1 * P6}{Kms1 * Kms6 * Keq}\right)\right) \Big/ \left(\left(1 + \frac{S1}{Kms1} + \frac{P1}{Kmp1} + \frac{S2}{Kms2} + \frac{P2}{Kmp2} + \frac{S3}{Kms3} + \frac{P3}{Kmp3} + \frac{S4}{Kms4} + \frac{P4}{Kmp4} + \frac{S5}{Kms5} + \frac{P5}{Kmp5}\right) * \left(1 + \frac{(S6 - P6)}{Kms6} + \frac{P6}{Kmp6}\right)\right)$$

MCAD[sf_, V_, Kms1_, Kms2_, Kms3_, Kms4_, Kms5_, Kms6_, Kmp1_, Kmp2_, Kmp3_, Kmp4_, Kmp5_, Kmp6_, Keq_, S1_, S2_, S3_, S4_, S5_, S6_, P1_, P2_, P3_, P4_, P5_, P6_] :=

$$\left(sf * V * \left(\frac{S1 * (S6 - P6)}{Kms1 * Kms6} - \frac{P1 * P6}{Kms1 * Kms6 * Keq}\right)\right) \Big/ \left(\left(1 + \frac{S1}{Kms1} + \frac{P1}{Kmp1} + \frac{S2}{Kms2} + \frac{P2}{Kmp2} + \frac{S3}{Kms3} + \frac{P3}{Kmp3} + \frac{S4}{Kms4} + \frac{P4}{Kmp4} + \frac{S5}{Kms5} + \frac{P5}{Kmp5}\right) * \left(1 + \frac{(S6 - P6)}{Kms6} + \frac{P6}{Kmp6}\right)\right)$$

SCAD[sf_, V_, Kms1_, Kms2_, Kms3_, Kmp1_, Kmp2_, Kmp3_, Keq_, S1_, S2_, S3_, P1_, P2_, P3_] :=

$$\frac{sf*V*\left(\frac{S1*(S3-P3)}{Kms1*Kms3}-\frac{P1*P3}{Kms1*Kms3*Keq}\right)}{\left(1+\frac{S1}{Kms1}+\frac{P1}{Kmp1}+\frac{S2}{Kms2}+\frac{P2}{Kmp2}\right)*\left(1+\frac{(S3-P3)}{Kms3}+\frac{P3}{Kmp3}\right)}$$

CROT[sf_, V_, Kms1_, Kms2_, Kms3_, Kms4_, Kms5_, Kms6_, Kms7_, Kmp1_, Kmp2_, Kmp3_, Kmp4_, Kmp5_, Kmp6_, Kmp7_, Ki1_, Keq_, S1_, S2_, S3_, S4_, S5_, S6_, S7_, P1_, P2_, P3_, P4_, P5_, P6_, P7_, I1_] :=

$$\left(sf*V*\left(\frac{S1}{Kms1}-\frac{P1}{Kms1*Keq}\right)\right)\Big/\left(1+\frac{S1}{Kms1}+\frac{P1}{Kmp1}+\frac{S2}{Kms2}+\frac{P2}{Kmp2}+\frac{S3}{Kms3}+\frac{P3}{Kmp3}+\frac{S4}{Kms4}+\frac{P4}{Kmp4}+\frac{S5}{Kms5}+\frac{P5}{Kmp5}+\frac{S6}{Kms6}+\frac{P6}{Kmp6}+\frac{S7}{Kms7}+\frac{P7}{Kmp7}+\frac{I1}{Ki1}\right)$$

MSCHAD[sf_, V_, Kms1_, Kms2_, Kms3_, Kms4_, Kms5_, Kms6_, Kms7_, Kms8_, Kmp1_, Kmp2_, Kmp3_, Kmp4_, Kmp5_, Kmp6_, Kmp7_, Kmp8_, Keq_, S1_, S2_, S3_, S4_, S5_, S6_, S7_, S8_, P1_, P2_, P3_, P4_, P5_, P6_, P7_, P8_] :=

$$\left(sf*V*\left(\frac{S1*(S8-P8)}{Kms1*Kms8}-\frac{P1*P8}{Kms1*Kms8*Keq}\right)\right)\Big/\left(\left(1+\frac{S1}{Kms1}+\frac{P1}{Kmp1}+\frac{S2}{Kms2}+\frac{P2}{Kmp2}+\frac{S3}{Kms3}+\frac{P3}{Kmp3}+\frac{S4}{Kms4}+\frac{P4}{Kmp4}+\frac{S5}{Kms5}+\frac{P5}{Kmp5}+\frac{S6}{Kms6}+\frac{P6}{Kmp6}+\frac{S7}{Kms7}+\frac{P7}{Kmp7}\right)*\left(1+\frac{(S8-P8)}{Kms8}+\frac{P8}{Kmp8}\right)\right)$$

MCKATA[sf_, V_, Kms1_, Kms2_, Kms3_, Kms4_, Kms5_, Kms6_, Kms7_, Kms8_, Kmp1_, Kmp2_, Kmp3_, Kmp4_, Kmp5_, Kmp6_, Kmp7_, Kmp8_, Keq_, S1_, S2_, S3_, S4_, S5_, S6_, S7_, S8_, P1_, P2_, P3_, P4_, P5_, P6_, P7_, P8_] :=

$$\left(sf*V*\left(\frac{S1*S8}{Kms1*Kms8}-\frac{P1*P8}{Kms1*Kms8*Keq}\right)\right)\Big/\left(\left(1+\frac{S1}{Kms1}+\frac{P1}{Kmp1}+\frac{S2}{Kms2}+\frac{P2}{Kmp2}+\frac{S3}{Kms3}+\frac{P3}{Kmp3}+\frac{S4}{Kms4}+\frac{P4}{Kmp4}+\frac{S5}{Kms5}+\frac{P5}{Kmp5}+\frac{S6}{Kms6}+\frac{P6}{Kmp6}+\frac{S7}{Kms7}+\frac{P7}{Kmp7}+\frac{P8}{Kmp8}\right)*\left(1+\frac{S8}{Kms8}+\frac{P8}{Kmp8}\right)\right)$$

MCKATB[sf_, V_, Kms1_, Kms2_, Kms3_, Kms4_, Kms5_, Kms6_, Kms7_, Kms8_, Kmp1_, Kmp2_, Kmp3_, Kmp4_, Kmp5_, Kmp6_, Kmp7_, Kmp8_, Keq_, S1_, S2_, S3_, S4_, S5_, S6_, S7_, S8_, P1_, P2_, P3_, P4_, P5_, P6_, P7_, P8_] :=

$$\left(sf*V*\left(\frac{S1*S8}{Kms1*Kms8}-\frac{P8*P8}{Kms1*Kms8*Keq}\right)\right)\Big/\left(\left(1+\frac{S1}{Kms1}+\frac{S2}{Kms2}+\frac{P2}{Kmp2}+\frac{S3}{Kms3}+\frac{P3}{Kmp3}+\frac{S4}{Kms4}+\frac{P4}{Kmp4}+\frac{S5}{Kms5}+\frac{P5}{Kmp5}+\frac{S6}{Kms6}+\frac{P6}{Kmp6}+\frac{S7}{Kms7}+\frac{P7}{Kmp7}+\frac{P8}{Kmp8}\right)*\left(1+\frac{S8}{Kms8}+\frac{P8}{Kmp8}\right)\right)$$

MTP[sf_, V_, Kms1_, Kms2_, Kms3_, Kms4_, Kms5_, Kms7_, Kms8_, Kmp1_, Kmp2_, Kmp3_, Kmp4_, Kmp5_, Kmp6_, Kmp7_, Kmp8_, Ki1_, Keq_, S1_, S2_, S3_, S4_, S5_, S7_, S8_, P1_, P2_, P3_, P4_, P5_, P6_, P7_, P8_, I1_] :=

$$\left(sf*V*\left(\frac{S1*(S7-P7)*S8}{Kms1*Kms7*Kms8}-\frac{P1*P7*P8}{Kms1*Kms7*Kms8*Keq}\right)\right)\Big/\left(\left(1+\frac{S1}{Kms1}+\frac{P1}{Kmp1}+\frac{S2}{Kms2}+\frac{P2}{Kmp2}+\frac{S3}{Kms3}+\frac{P3}{Kmp3}+\frac{S4}{Kms4}+\frac{P4}{Kmp4}+\frac{S5}{Kms5}+\frac{P5}{Kmp5}+\frac{P6}{Kmp6}+\frac{I1}{Ki1}\right)*\left(1+\frac{(S7-P7)}{Kms7}+\frac{P7}{Kmp7}\right)*\left(1+\frac{S8}{Kms8}+\frac{P8}{Kmp8}\right)\right)$$

```
RES[Ks_, S_, K1_] := Ks * (S - K1)
```

Define the differential equations

```
Odes = {
   C16AcylCarCYT'[t] == (vcpt1C16 - vcactC16)/VCYT,
   C16AcylCarMAT'[t] == (vcactC16 - vcpt2C16)/VMAT,
   C16AcylCoAMAT'[t] == (vcpt2C16 - vvlcadC16 - vlcadC16)/VMAT,
   C16EnoylCoAMAT'[t] == (vvlcadC16 + vlcadC16 - vcrotC16 - vmtpC16)/VMAT,
   C16HydroxyacylCoAMAT'[t] == (vcrotC16 - vmschadC16)/VMAT,
   C16KetoacylCoAMAT'[t] == (vmschadC16 - vmckatC16)/VMAT,
   C14AcylCarCYT'[t] == (-vcactC14)/VCYT,
   C14AcylCarMAT'[t] == (vcactC14 - vcpt2C14)/VMAT,
   C14AcylCoAMAT'[t] == 1/VMAT (vcpt2C14 + vmtpC16 + vmckatC16 - vvlcadC14 - vlcadC14),
   C14EnoylCoAMAT'[t] == (vvlcadC14 + vlcadC14 - vcrotC14 - vmtpC14)/VMAT,
   C14HydroxyacylCoAMAT'[t] == (vcrotC14 - vmschadC14)/VMAT,
   C14KetoacylCoAMAT'[t] == (vmschadC14 - vmckatC14)/VMAT,
   C12AcylCarCYT'[t] == (-vcactC12)/VCYT,
   C12AcylCarMAT'[t] == (vcactC12 - vcpt2C12)/VMAT,
   C12AcylCoAMAT'[t] ==
    1/VMAT (vcpt2C12 + vmtpC14 + vmckatC14 - vvlcadC12 - vlcadC12 - vmcadC12),
   C12EnoylCoAMAT'[t] == 1/VMAT (vvlcadC12 + vlcadC12 + vmcadC12 - vcrotC12 - vmtpC12),
   C12HydroxyacylCoAMAT'[t] == (vcrotC12 - vmschadC12)/VMAT,
   C12KetoacylCoAMAT'[t] == (vmschadC12 - vmckatC12)/VMAT,
   C10AcylCarCYT'[t] == (-vcactC10)/VCYT,
   C10AcylCarMAT'[t] == (vcactC10 - vcpt2C10)/VMAT,
```

$$\text{C10AcylCoAMAT}'[t] == \frac{1}{\text{VMAT}}(\text{vcpt2C10} + \text{vmtpC12} + \text{vmckatC12} - \text{vlcadC10} - \text{vmcadC10}),$$

$$\text{C10EnoylCoAMAT}'[t] == \frac{\text{vlcadC10} + \text{vmcadC10} - \text{vcrotC10} - \text{vmtpC10}}{\text{VMAT}},$$

$$\text{C10HydroxyacylCoAMAT}'[t] == \frac{\text{vcrotC10} - \text{vmschadC10}}{\text{VMAT}},$$

$$\text{C10KetoacylCoAMAT}'[t] == \frac{\text{vmschadC10} - \text{vmckatC10}}{\text{VMAT}},$$

$$\text{C8AcylCarCYT}'[t] == \frac{-\text{vcactC8}}{\text{VCYT}},$$

$$\text{C8AcylCarMAT}'[t] == \frac{\text{vcactC8} - \text{vcpt2C8}}{\text{VMAT}},$$

$$\text{C8AcylCoAMAT}'[t] == \frac{\text{vcpt2C8} + \text{vmtpC10} + \text{vmckatC10} - \text{vlcadC8} - \text{vmcadC8}}{\text{VMAT}},$$

$$\text{C8EnoylCoAMAT}'[t] == \frac{\text{vlcadC8} + \text{vmcadC8} - \text{vcrotC8} - \text{vmtpC8}}{\text{VMAT}},$$

$$\text{C8HydroxyacylCoAMAT}'[t] == \frac{\text{vcrotC8} - \text{vmschadC8}}{\text{VMAT}},$$

$$\text{C8KetoacylCoAMAT}'[t] == \frac{\text{vmschadC8} - \text{vmckatC8}}{\text{VMAT}},$$

$$\text{C6AcylCarCYT}'[t] == \frac{-\text{vcactC6}}{\text{VCYT}},$$

$$\text{C6AcylCarMAT}'[t] == \frac{\text{vcactC6} - \text{vcpt2C6}}{\text{VMAT}},$$

$$\text{C6AcylCoAMAT}'[t] == \frac{\text{vcpt2C6} + \text{vmtpC8} + \text{vmckatC8} - \text{vmcadC6} - \text{vscadC6}}{\text{VMAT}},$$

$$\text{C6EnoylCoAMAT}'[t] == \frac{\text{vmcadC6} + \text{vscadC6} - \text{vcrotC6}}{\text{VMAT}},$$

$$\text{C6HydroxyacylCoAMAT}'[t] == \frac{\text{vcrotC6} - \text{vmschadC6}}{\text{VMAT}},$$

$$\text{C6KetoacylCoAMAT}'[t] == \frac{\text{vmschadC6} - \text{vmckatC6}}{\text{VMAT}},$$

$$\text{C4AcylCarCYT}'[t] == \frac{-\text{vcactC4}}{\text{VCYT}},$$

$$\text{C4AcylCarMAT}'[t] == \frac{\text{vcactC4} - \text{vcpt2C4}}{\text{VMAT}},$$

$$\text{C4AcylCoAMAT}'[t] == \frac{\text{vcpt2C4} + \text{vmckatC6} - \text{vmcadC4} - \text{vscadC4}}{\text{VMAT}},$$

$$\text{C4EnoylCoAMAT}'[t] == \frac{\text{vmcadC4} + \text{vscadC4} - \text{vcrotC4}}{\text{VMAT}},$$

$$\text{C4HydroxyacylCoAMAT}'[t] == \frac{\text{vcrotC4} - \text{vmschadC4}}{\text{VMAT}},$$

$$\text{C4AcetoacylCoAMAT}'[t] == \frac{\text{vmschadC4} - \text{vmckatC4}}{\text{VMAT}},$$

$$\text{AcetylCoAMAT}'[t] ==$$

$$\frac{1}{\text{VMAT}}(\text{vmtpC16} + \text{vmckatC16} + \text{vmtpC14} + \text{vmckatC14} + \text{vmtpC12} + \text{vmckatC12} + \text{vmtpC10} +$$

```
      vmckatC10 + vmtpC8 + vmckatC8 + vmckatC6 + 2 * vmckatC4 - vacesink),
   FADHMAT'[t] == 1/VMAT (vvlcadC16 + vvlcadC14 + vvlcadC12 + vlcadC16 +
      vlcadC14 + vlcadC12 + vlcadC10 + vlcadC8 + vmcadC12 + vmcadC10 +
      vmcadC8 + vmcadC6 + vmcadC4 + vscadC6 + vscadC4 - vfadhsink),
   NADHMAT'[t] == 1/VMAT (vmtpC16 + vmtpC14 + vmtpC12 + vmtpC10 + vmtpC8 +
      vmschadC16 + vmschadC14 + vmschadC12 + vmschadC10 +
      vmschadC8 + vmschadC6 + vmschadC4 - vnadhsink)};

RateEqs = {
   vcpt1C16 → CPT1[sfcpt1C16, Vcpt1, Kmcpt1C16AcylCoACYT, Kmcpt1CarCYT,
     Kmcpt1C16AcylCarCYT, Kmcpt1CoACYT, Kicpt1MalCoACYT, Keqcpt1,
     C16AcylCoACYT, CarCYT, C16AcylCarCYT[t], CoACYT, MalCoACYT, ncpt1],
   vcactC16 → CACT[Vfcact, Vrcact, KmcactC16AcylCarCYT, KmcactCarMAT,
     KmcactC16AcylCarMAT, KmcactCarCYT, KicactC16AcylCarCYT, KicactCarCYT,
     Keqcact, C16AcylCarCYT[t], CarMAT, C16AcylCarMAT[t], CarCYT],
   vcactC14 → CACT[Vfcact, Vrcact, KmcactC14AcylCarCYT, KmcactCarMAT,
     KmcactC14AcylCarMAT, KmcactCarCYT, KicactC14AcylCarCYT, KicactCarCYT,
     Keqcact, C14AcylCarCYT[t], CarMAT, C14AcylCarMAT[t], CarCYT],
   vcactC12 → CACT[Vfcact, Vrcact, KmcactC12AcylCarCYT, KmcactCarMAT,
     KmcactC12AcylCarMAT, KmcactCarCYT, KicactC12AcylCarCYT, KicactCarCYT,
     Keqcact, C12AcylCarCYT[t], CarMAT, C12AcylCarMAT[t], CarCYT],
   vcactC10 → CACT[Vfcact, Vrcact, KmcactC10AcylCarCYT, KmcactCarMAT,
     KmcactC10AcylCarMAT, KmcactCarCYT, KicactC10AcylCarCYT, KicactCarCYT,
     Keqcact, C10AcylCarCYT[t], CarMAT, C10AcylCarMAT[t], CarCYT],
   vcactC8 → CACT[Vfcact, Vrcact, KmcactC8AcylCarCYT, KmcactCarMAT,
     KmcactC8AcylCarMAT, KmcactCarCYT, KicactC8AcylCarCYT, KicactCarCYT,
     Keqcact, C8AcylCarCYT[t], CarMAT, C8AcylCarMAT[t], CarCYT],
   vcactC6 → CACT[Vfcact, Vrcact, KmcactC6AcylCarCYT, KmcactCarMAT,
     KmcactC6AcylCarMAT, KmcactCarCYT, KicactC6AcylCarCYT, KicactCarCYT,
     Keqcact, C6AcylCarCYT[t], CarMAT, C6AcylCarMAT[t], CarCYT],
   vcactC4 → CACT[Vfcact, Vrcact, KmcactC4AcylCarCYT, KmcactCarMAT,
     KmcactC4AcylCarMAT, KmcactCarCYT, KicactC4AcylCarCYT, KicactCarCYT,
     Keqcact, C4AcylCarCYT[t], CarMAT, C4AcylCarMAT[t], CarCYT],
   vcpt2C16 → CPT2[sfcpt2C16, Vcpt2, Kmcpt2C16AcylCarMAT, Kmcpt2C14AcylCarMAT,
     Kmcpt2C12AcylCarMAT, Kmcpt2C10AcylCarMAT, Kmcpt2C8AcylCarMAT, Kmcpt2C6AcylCarMAT,
     Kmcpt2C4AcylCarMAT, Kmcpt2CoAMAT, Kmcpt2C16AcylCoAMAT, Kmcpt2C14AcylCoAMAT,
     Kmcpt2C12AcylCoAMAT, Kmcpt2C10AcylCoAMAT, Kmcpt2C8AcylCoAMAT, Kmcpt2C6AcylCoAMAT,
     Kmcpt2C4AcylCoAMAT, Kmcpt2CarMAT, Keqcpt2, C16AcylCarMAT[t], C14AcylCarMAT[t],
     C12AcylCarMAT[t], C10AcylCarMAT[t], C8AcylCarMAT[t], C6AcylCarMAT[t],
     C4AcylCarMAT[t], CoAMAT, C16AcylCoAMAT[t], C14AcylCoAMAT[t], C12AcylCoAMAT[t],
     C10AcylCoAMAT[t], C8AcylCoAMAT[t], C6AcylCoAMAT[t], C4AcylCoAMAT[t], CarMAT],
   vcpt2C14 → CPT2[sfcpt2C14, Vcpt2, Kmcpt2C14AcylCarMAT, Kmcpt2C16AcylCarMAT,
     Kmcpt2C12AcylCarMAT, Kmcpt2C10AcylCarMAT, Kmcpt2C8AcylCarMAT, Kmcpt2C6AcylCarMAT,
     Kmcpt2C4AcylCoAMAT, Kmcpt2CoAMAT, Kmcpt2C14AcylCoAMAT, Kmcpt2C16AcylCoAMAT,
     Kmcpt2C12AcylCoAMAT, Kmcpt2C10AcylCoAMAT, Kmcpt2C8AcylCoAMAT, Kmcpt2C6AcylCoAMAT,
     Kmcpt2C4AcylCoAMAT, Kmcpt2CarMAT, Keqcpt2, C14AcylCarMAT[t], C16AcylCarMAT[t],
     C12AcylCarMAT[t], C10AcylCarMAT[t], C8AcylCarMAT[t], C6AcylCarMAT[t],
```

```
    C4AcylCarMAT[t], CoAMAT, C14AcylCoAMAT[t], C16AcylCoAMAT[t], C12AcylCoAMAT[t],
    C10AcylCoAMAT[t], C8AcylCoAMAT[t], C6AcylCoAMAT[t], C4AcylCoAMAT[t], CarMAT],
  vcpt2C12 → CPT2[sfcpt2C12, Vcpt2, Kmcpt2C12AcylCarMAT, Kmcpt2C16AcylCarMAT,
    Kmcpt2C14AcylCarMAT, Kmcpt2C10AcylCarMAT, Kmcpt2C8AcylCarMAT, Kmcpt2C6AcylCarMAT,
    Kmcpt2C4AcylCarMAT, Kmcpt2CoAMAT, Kmcpt2C12AcylCoAMAT, Kmcpt2C16AcylCoAMAT,
    Kmcpt2C14AcylCoAMAT, Kmcpt2C10AcylCoAMAT, Kmcpt2C8AcylCoAMAT, Kmcpt2C6AcylCoAMAT,
    Kmcpt2C4AcylCoAMAT, Kmcpt2CarMAT, Keqcpt2, C12AcylCarMAT[t], C16AcylCarMAT[t],
    C14AcylCarMAT[t], C10AcylCarMAT[t], C8AcylCarMAT[t], C6AcylCarMAT[t],
    C4AcylCarMAT[t], CoAMAT, C12AcylCoAMAT[t], C16AcylCoAMAT[t], C14AcylCoAMAT[t],
    C10AcylCoAMAT[t], C8AcylCoAMAT[t], C6AcylCoAMAT[t], C4AcylCoAMAT[t], CarMAT],
  vcpt2C10 → CPT2[sfcpt2C10, Vcpt2, Kmcpt2C10AcylCarMAT, Kmcpt2C16AcylCarMAT,
    Kmcpt2C14AcylCarMAT, Kmcpt2C12AcylCarMAT, Kmcpt2C8AcylCarMAT, Kmcpt2C6AcylCarMAT,
    Kmcpt2C4AcylCarMAT, Kmcpt2CoAMAT, Kmcpt2C10AcylCoAMAT, Kmcpt2C16AcylCoAMAT,
    Kmcpt2C14AcylCoAMAT, Kmcpt2C12AcylCoAMAT, Kmcpt2C8AcylCoAMAT, Kmcpt2C6AcylCoAMAT,
    Kmcpt2C4AcylCoAMAT, Kmcpt2CarMAT, Keqcpt2, C10AcylCarMAT[t], C16AcylCarMAT[t],
    C14AcylCarMAT[t], C12AcylCarMAT[t], C8AcylCarMAT[t], C6AcylCarMAT[t],
    C4AcylCarMAT[t], CoAMAT, C10AcylCoAMAT[t], C16AcylCoAMAT[t], C14AcylCoAMAT[t],
    C12AcylCoAMAT[t], C8AcylCoAMAT[t], C6AcylCoAMAT[t], C4AcylCoAMAT[t], CarMAT],
  vcpt2C8 → CPT2[sfcpt2C8, Vcpt2, Kmcpt2C8AcylCarMAT, Kmcpt2C16AcylCarMAT,
    Kmcpt2C14AcylCarMAT, Kmcpt2C12AcylCarMAT, Kmcpt2C10AcylCarMAT, Kmcpt2C6AcylCarMAT,
    Kmcpt2C4AcylCarMAT, Kmcpt2CoAMAT, Kmcpt2C8AcylCoAMAT, Kmcpt2C16AcylCoAMAT,
    Kmcpt2C14AcylCoAMAT, Kmcpt2C12AcylCoAMAT, Kmcpt2C10AcylCoAMAT, Kmcpt2C6AcylCoAMAT,
    Kmcpt2C4AcylCoAMAT, Kmcpt2CarMAT, Keqcpt2, C8AcylCarMAT[t], C16AcylCarMAT[t],
    C14AcylCarMAT[t], C12AcylCarMAT[t], C10AcylCarMAT[t], C6AcylCarMAT[t],
    C4AcylCarMAT[t], CoAMAT, C8AcylCoAMAT[t], C16AcylCoAMAT[t], C14AcylCoAMAT[t],
    C12AcylCoAMAT[t], C10AcylCoAMAT[t], C6AcylCoAMAT[t], C4AcylCoAMAT[t], CarMAT],
  vcpt2C6 → CPT2[sfcpt2C6, Vcpt2, Kmcpt2C6AcylCarMAT, Kmcpt2C16AcylCarMAT,
    Kmcpt2C14AcylCarMAT, Kmcpt2C12AcylCarMAT, Kmcpt2C10AcylCarMAT, Kmcpt2C8AcylCarMAT,
    Kmcpt2C4AcylCarMAT, Kmcpt2CoAMAT, Kmcpt2C6AcylCoAMAT, Kmcpt2C16AcylCoAMAT,
    Kmcpt2C14AcylCoAMAT, Kmcpt2C12AcylCoAMAT, Kmcpt2C10AcylCoAMAT, Kmcpt2C8AcylCoAMAT,
    Kmcpt2C4AcylCoAMAT, Kmcpt2CarMAT, Keqcpt2, C6AcylCarMAT[t], C16AcylCarMAT[t],
    C14AcylCarMAT[t], C12AcylCarMAT[t], C10AcylCarMAT[t], C8AcylCarMAT[t],
    C4AcylCarMAT[t], CoAMAT, C6AcylCoAMAT[t], C16AcylCoAMAT[t], C14AcylCoAMAT[t],
    C12AcylCoAMAT[t], C10AcylCoAMAT[t], C8AcylCoAMAT[t], C4AcylCoAMAT[t], CarMAT],
  vcpt2C4 → CPT2[sfcpt2C4, Vcpt2, Kmcpt2C4AcylCarMAT, Kmcpt2C16AcylCarMAT,
    Kmcpt2C14AcylCarMAT, Kmcpt2C12AcylCarMAT, Kmcpt2C10AcylCarMAT, Kmcpt2C8AcylCarMAT,
    Kmcpt2C6AcylCarMAT, Kmcpt2CoAMAT, Kmcpt2C4AcylCoAMAT, Kmcpt2C16AcylCoAMAT,
    Kmcpt2C14AcylCoAMAT, Kmcpt2C12AcylCoAMAT, Kmcpt2C10AcylCoAMAT, Kmcpt2C8AcylCoAMAT,
    Kmcpt2C6AcylCoAMAT, Kmcpt2CarMAT, Keqcpt2, C4AcylCarMAT[t], C16AcylCarMAT[t],
    C14AcylCarMAT[t], C12AcylCarMAT[t], C10AcylCarMAT[t], C8AcylCarMAT[t],
    C6AcylCarMAT[t], CoAMAT, C4AcylCoAMAT[t], C16AcylCoAMAT[t], C14AcylCoAMAT[t],
    C12AcylCoAMAT[t], C10AcylCoAMAT[t], C8AcylCoAMAT[t], C6AcylCoAMAT[t], CarMAT],
  vvlcadC16 → VLCAD[sfvlcadC16, Vvlcad, KmvlcadC16AcylCoAMAT, KmvlcadC14AcylCoAMAT,
    KmvlcadC12AcylCoAMAT, KmvlcadFAD, KmvlcadC16EnoylCoAMAT,
    KmvlcadC14EnoylCoAMAT, KmvlcadC12EnoylCoAMAT, KmvlcadFADH, Keqvlcad,
    C16AcylCoAMAT[t], C14AcylCoAMAT[t], C12AcylCoAMAT[t], FADtMAT,
    C16EnoylCoAMAT[t], C14EnoylCoAMAT[t], C12EnoylCoAMAT[t], FADHMAT[t]],
  vvlcadC14 → VLCAD[sfvlcadC14, Vvlcad, KmvlcadC14AcylCoAMAT, KmvlcadC16AcylCoAMAT,
    KmvlcadC12AcylCoAMAT, KmvlcadFAD, KmvlcadC14EnoylCoAMAT,
    KmvlcadC16EnoylCoAMAT, KmvlcadC12EnoylCoAMAT, KmvlcadFADH, Keqvlcad,
```

```
   C14AcylCoAMAT[t], C16AcylCoAMAT[t], C12AcylCoAMAT[t], FADtMAT,
   C14EnoylCoAMAT[t], C16EnoylCoAMAT[t], C12EnoylCoAMAT[t], FADHMAT[t]],
vvlcadC12 → VLCAD[sfvlcadC12, Vvlcad, KmvlcadC12AcylCoAMAT, KmvlcadC16AcylCoAMAT,
   KmvlcadC14AcylCoAMAT, KmvlcadFAD, KmvlcadC12EnoylCoAMAT,
   KmvlcadC16EnoylCoAMAT, KmvlcadC14EnoylCoAMAT, KmvlcadFADH, Keqvlcad,
   C12AcylCoAMAT[t], C16AcylCoAMAT[t], C14AcylCoAMAT[t], FADtMAT,
   C12EnoylCoAMAT[t], C16EnoylCoAMAT[t], C14EnoylCoAMAT[t], FADHMAT[t]],
vlcadC16 → LCAD[sflcadC16, Vlcad, KmlcadC16AcylCoAMAT, KmlcadC14AcylCoAMAT,
   KmlcadC12AcylCoAMAT, KmlcadC10AcylCoAMAT, KmlcadC8AcylCoAMAT, KmlcadFAD,
   KmlcadC16EnoylCoAMAT, KmlcadC14EnoylCoAMAT, KmlcadC12EnoylCoAMAT,
   KmlcadC10EnoylCoAMAT, KmlcadC8EnoylCoAMAT, KmlcadFADH, Keqlcad,
   C16AcylCoAMAT[t], C14AcylCoAMAT[t], C12AcylCoAMAT[t], C10AcylCoAMAT[t],
   C8AcylCoAMAT[t], FADtMAT, C16EnoylCoAMAT[t], C14EnoylCoAMAT[t],
   C12EnoylCoAMAT[t], C10EnoylCoAMAT[t], C8EnoylCoAMAT[t], FADHMAT[t]],
vlcadC14 → LCAD[sflcadC14, Vlcad, KmlcadC14AcylCoAMAT, KmlcadC16AcylCoAMAT,
   KmlcadC12AcylCoAMAT, KmlcadC10AcylCoAMAT, KmlcadC8AcylCoAMAT, KmlcadFAD,
   KmlcadC14EnoylCoAMAT, KmlcadC16EnoylCoAMAT, KmlcadC12EnoylCoAMAT,
   KmlcadC10EnoylCoAMAT, KmlcadC8EnoylCoAMAT, KmlcadFADH, Keqlcad,
   C14AcylCoAMAT[t], C16AcylCoAMAT[t], C12AcylCoAMAT[t], C10AcylCoAMAT[t],
   C8AcylCoAMAT[t], FADtMAT, C14EnoylCoAMAT[t], C16EnoylCoAMAT[t],
   C12EnoylCoAMAT[t], C10EnoylCoAMAT[t], C8EnoylCoAMAT[t], FADHMAT[t]],
vlcadC12 → LCAD[sflcadC12, Vlcad, KmlcadC12AcylCoAMAT, KmlcadC16AcylCoAMAT,
   KmlcadC14AcylCoAMAT, KmlcadC10AcylCoAMAT, KmlcadC8AcylCoAMAT, KmlcadFAD,
   KmlcadC12EnoylCoAMAT, KmlcadC16EnoylCoAMAT, KmlcadC14EnoylCoAMAT,
   KmlcadC10EnoylCoAMAT, KmlcadC8EnoylCoAMAT, KmlcadFADH, Keqlcad,
   C12AcylCoAMAT[t], C16AcylCoAMAT[t], C14AcylCoAMAT[t], C10AcylCoAMAT[t],
   C8AcylCoAMAT[t], FADtMAT, C14EnoylCoAMAT[t], C16EnoylCoAMAT[t],
   C14EnoylCoAMAT[t], C10EnoylCoAMAT[t], C8EnoylCoAMAT[t], FADHMAT[t]],
vlcadC10 → LCAD[sflcadC10, Vlcad, KmlcadC10AcylCoAMAT, KmlcadC16AcylCoAMAT,
   KmlcadC14AcylCoAMAT, KmlcadC12AcylCoAMAT, KmlcadC8AcylCoAMAT, KmlcadFAD,
   KmlcadC10EnoylCoAMAT, KmlcadC16EnoylCoAMAT, KmlcadC14EnoylCoAMAT,
   KmlcadC12EnoylCoAMAT, KmlcadC8EnoylCoAMAT, KmlcadFADH, Keqlcad,
   C10AcylCoAMAT[t], C16AcylCoAMAT[t], C14AcylCoAMAT[t], C12AcylCoAMAT[t],
   C8AcylCoAMAT[t], FADtMAT, C10EnoylCoAMAT[t], C16EnoylCoAMAT[t],
   C14EnoylCoAMAT[t], C12EnoylCoAMAT[t], C8EnoylCoAMAT[t], FADHMAT[t]],
vlcadC8 → LCAD[sflcadC8, Vlcad, KmlcadC8AcylCoAMAT, KmlcadC16AcylCoAMAT,
   KmlcadC14AcylCoAMAT, KmlcadC12AcylCoAMAT, KmlcadC10AcylCoAMAT, KmlcadFAD,
   KmlcadC8EnoylCoAMAT, KmlcadC16EnoylCoAMAT, KmlcadC14EnoylCoAMAT,
   KmlcadC12EnoylCoAMAT, KmlcadC10EnoylCoAMAT, KmlcadFADH, Keqlcad,
   C8AcylCoAMAT[t], C16AcylCoAMAT[t], C14AcylCoAMAT[t], C12AcylCoAMAT[t],
   C10AcylCoAMAT[t], FADtMAT, C8EnoylCoAMAT[t], C16EnoylCoAMAT[t],
   C14EnoylCoAMAT[t], C12EnoylCoAMAT[t], C10EnoylCoAMAT[t], FADHMAT[t]],
vmcadC12 → MCAD[sfmcadC12, Vmcad, KmmcadC12AcylCoAMAT, KmmcadC10AcylCoAMAT,
   KmmcadC8AcylCoAMAT, KmmcadC6AcylCoAMAT, KmmcadC4AcylCoAMAT, KmmcadFAD,
   KmmcadC12EnoylCoAMAT, KmmcadC10EnoylCoAMAT, KmmcadC8EnoylCoAMAT,
   KmmcadC6EnoylCoAMAT, KmmcadC4EnoylCoAMAT, KmmcadFADH, Keqmcad,
   C12AcylCoAMAT[t], C10AcylCoAMAT[t], C8AcylCoAMAT[t], C6AcylCoAMAT[t],
   C4AcylCoAMAT[t], FADtMAT, C12EnoylCoAMAT[t], C10EnoylCoAMAT[t],
   C8EnoylCoAMAT[t], C6EnoylCoAMAT[t], C4EnoylCoAMAT[t], FADHMAT[t]],
vmcadC10 → MCAD[sfmcadC10, Vmcad, KmmcadC10AcylCoAMAT, KmmcadC12AcylCoAMAT,
```

```
    KmmcadC8AcylCoAMAT, KmmcadC6AcylCoAMAT, KmmcadC4AcylCoAMAT, KmmcadFAD,
    KmmcadC10EnoylCoAMAT, KmmcadC12EnoylCoAMAT, KmmcadC8EnoylCoAMAT,
    KmmcadC6EnoylCoAMAT, KmmcadC4EnoylCoAMAT, KmmcadFADH, Keqmcad,
    C10AcylCoAMAT[t], C12AcylCoAMAT[t], C8AcylCoAMAT[t], C6AcylCoAMAT[t],
    C4AcylCoAMAT[t], FADtMAT, C10EnoylCoAMAT[t], C12EnoylCoAMAT[t],
    C8EnoylCoAMAT[t], C6EnoylCoAMAT[t], C4EnoylCoAMAT[t], FADHMAT[t]],
  vmcadC8 → MCAD[sfmcadC8, Vmcad, KmmcadC8AcylCoAMAT, KmmcadC12AcylCoAMAT,
    KmmcadC10AcylCoAMAT, KmmcadC6AcylCoAMAT, KmmcadC4AcylCoAMAT, KmmcadFAD,
    KmmcadC8EnoylCoAMAT, KmmcadC12EnoylCoAMAT, KmmcadC10EnoylCoAMAT,
    KmmcadC6EnoylCoAMAT, KmmcadC4EnoylCoAMAT, KmmcadFADH, Keqmcad,
    C8AcylCoAMAT[t], C12AcylCoAMAT[t], C10AcylCoAMAT[t], C6AcylCoAMAT[t],
    C4AcylCoAMAT[t], FADtMAT, C8EnoylCoAMAT[t], C12EnoylCoAMAT[t],
    C10EnoylCoAMAT[t], C6EnoylCoAMAT[t], C4EnoylCoAMAT[t], FADHMAT[t]],
  vmcadC6 → MCAD[sfmcadC6, Vmcad, KmmcadC6AcylCoAMAT, KmmcadC12AcylCoAMAT,
    KmmcadC10AcylCoAMAT, KmmcadC8AcylCoAMAT, KmmcadC4AcylCoAMAT, KmmcadFAD,
    KmmcadC6EnoylCoAMAT, KmmcadC12EnoylCoAMAT, KmmcadC10EnoylCoAMAT,
    KmmcadC8EnoylCoAMAT, KmmcadC4EnoylCoAMAT, KmmcadFADH, Keqmcad,
    C6AcylCoAMAT[t], C12AcylCoAMAT[t], C10AcylCoAMAT[t], C8AcylCoAMAT[t],
    C4AcylCoAMAT[t], FADtMAT, C6EnoylCoAMAT[t], C12EnoylCoAMAT[t],
    C10EnoylCoAMAT[t], C8EnoylCoAMAT[t], C4EnoylCoAMAT[t], FADHMAT[t]],
  vmcadC4 → MCAD[sfmcadC4, Vmcad, KmmcadC4AcylCoAMAT, KmmcadC12AcylCoAMAT,
    KmmcadC10AcylCoAMAT, KmmcadC8AcylCoAMAT, KmmcadC6AcylCoAMAT, KmmcadFAD,
    KmmcadC4EnoylCoAMAT, KmmcadC12EnoylCoAMAT, KmmcadC10EnoylCoAMAT,
    KmmcadC8EnoylCoAMAT, KmmcadC6EnoylCoAMAT, KmmcadFADH, Keqmcad,
    C4AcylCoAMAT[t], C12AcylCoAMAT[t], C10AcylCoAMAT[t], C8AcylCoAMAT[t],
    C6AcylCoAMAT[t], FADtMAT, C4EnoylCoAMAT[t], C12EnoylCoAMAT[t],
    C10EnoylCoAMAT[t], C8EnoylCoAMAT[t], C6EnoylCoAMAT[t], FADHMAT[t]],
  vscadC6 → SCAD[sfscadC6, Vscad, KmscadC6AcylCoAMAT, KmscadC4AcylCoAMAT, KmscadFAD,
    KmscadC6EnoylCoAMAT, KmscadC4EnoylCoAMAT, KmscadFADH, Keqscad, C6AcylCoAMAT[t],
    C4AcylCoAMAT[t], FADtMAT, C6EnoylCoAMAT[t], C4EnoylCoAMAT[t], FADHMAT[t]],
  vscadC4 → SCAD[sfscadC4, Vscad, KmscadC4AcylCoAMAT, KmscadC6AcylCoAMAT, KmscadFAD,
    KmscadC4EnoylCoAMAT, KmscadC6EnoylCoAMAT, KmscadFADH, Keqscad, C4AcylCoAMAT[t],
    C6AcylCoAMAT[t], FADtMAT, C4EnoylCoAMAT[t], C6EnoylCoAMAT[t], FADHMAT[t]],
  vcrotC16 → CROT[sfcrotC16, Vcrot, KmcrotC16EnoylCoAMAT, KmcrotC14EnoylCoAMAT,
    KmcrotC12EnoylCoAMAT, KmcrotC10EnoylCoAMAT, KmcrotC8EnoylCoAMAT,
    KmcrotC6EnoylCoAMAT, KmcrotC4EnoylCoAMAT, KmcrotC16HydroxyacylCoAMAT,
    KmcrotC14HydroxyacylCoAMAT, KmcrotC12HydroxyacylCoAMAT,
    KmcrotC10HydroxyacylCoAMAT, KmcrotC8HydroxyacylCoAMAT,
    KmcrotC6HydroxyacylCoAMAT, KmcrotC4HydroxyacylCoAMAT, KicrotC4AcetoacylCoA,
    Keqcrot, C16EnoylCoAMAT[t], C14EnoylCoAMAT[t], C12EnoylCoAMAT[t],
    C10EnoylCoAMAT[t], C8EnoylCoAMAT[t], C6EnoylCoAMAT[t],
    C4EnoylCoAMAT[t], C16HydroxyacylCoAMAT[t], C14HydroxyacylCoAMAT[t],
    C12HydroxyacylCoAMAT[t], C10HydroxyacylCoAMAT[t], C8HydroxyacylCoAMAT[t],
    C6HydroxyacylCoAMAT[t], C4HydroxyacylCoAMAT[t], C4AcetoacylCoAMAT[t]],
  vcrotC14 → CROT[sfcrotC14, Vcrot, KmcrotC14EnoylCoAMAT, KmcrotC16EnoylCoAMAT,
    KmcrotC12EnoylCoAMAT, KmcrotC10EnoylCoAMAT, KmcrotC8EnoylCoAMAT,
    KmcrotC6EnoylCoAMAT, KmcrotC4EnoylCoAMAT, KmcrotC14HydroxyacylCoAMAT,
    KmcrotC16HydroxyacylCoAMAT, KmcrotC12HydroxyacylCoAMAT,
    KmcrotC10HydroxyacylCoAMAT, KmcrotC8HydroxyacylCoAMAT,
    KmcrotC6HydroxyacylCoAMAT, KmcrotC4HydroxyacylCoAMAT, KicrotC4AcetoacylCoA,
```

```
  Keqcrot, C14EnoylCoAMAT[t], C16EnoylCoAMAT[t], C12EnoylCoAMAT[t],
  C10EnoylCoAMAT[t], C8EnoylCoAMAT[t], C6EnoylCoAMAT[t],
  C4EnoylCoAMAT[t], C14HydroxyacylCoAMAT[t], C16HydroxyacylCoAMAT[t],
  C12HydroxyacylCoAMAT[t], C10HydroxyacylCoAMAT[t], C8HydroxyacylCoAMAT[t],
  C6HydroxyacylCoAMAT[t], C4HydroxyacylCoAMAT[t], C4AcetoacylCoAMAT[t]],
vcrotC12 → CROT[sfcrotC12, Vcrot, KmcrotC12EnoylCoAMAT, KmcrotC16EnoylCoAMAT,
  KmcrotC14EnoylCoAMAT, KmcrotC10EnoylCoAMAT, KmcrotC8EnoylCoAMAT,
  KmcrotC6EnoylCoAMAT, KmcrotC4EnoylCoAMAT, KmcrotC12HydroxyacylCoAMAT,
  KmcrotC16HydroxyacylCoAMAT, KmcrotC14HydroxyacylCoAMAT,
  KmcrotC10HydroxyacylCoAMAT, KmcrotC8HydroxyacylCoAMAT,
  KmcrotC6HydroxyacylCoAMAT, KmcrotC4HydroxyacylCoAMAT, KicrotC4AcetoacylCoA,
  Keqcrot, C12EnoylCoAMAT[t], C16EnoylCoAMAT[t], C14EnoylCoAMAT[t],
  C10EnoylCoAMAT[t], C8EnoylCoAMAT[t], C6EnoylCoAMAT[t],
  C4EnoylCoAMAT[t], C12HydroxyacylCoAMAT[t], C16HydroxyacylCoAMAT[t],
  C14HydroxyacylCoAMAT[t], C10HydroxyacylCoAMAT[t], C8HydroxyacylCoAMAT[t],
  C6HydroxyacylCoAMAT[t], C4HydroxyacylCoAMAT[t], C4AcetoacylCoAMAT[t]],
vcrotC10 → CROT[sfcrotC10, Vcrot, KmcrotC10EnoylCoAMAT, KmcrotC16EnoylCoAMAT,
  KmcrotC14EnoylCoAMAT, KmcrotC12EnoylCoAMAT, KmcrotC8EnoylCoAMAT,
  KmcrotC6EnoylCoAMAT, KmcrotC4EnoylCoAMAT, KmcrotC10HydroxyacylCoAMAT,
  KmcrotC16HydroxyacylCoAMAT, KmcrotC14HydroxyacylCoAMAT,
  KmcrotC12HydroxyacylCoAMAT, KmcrotC8HydroxyacylCoAMAT,
  KmcrotC6HydroxyacylCoAMAT, KmcrotC4HydroxyacylCoAMAT, KicrotC4AcetoacylCoA,
  Keqcrot, C10EnoylCoAMAT[t], C16EnoylCoAMAT[t], C14EnoylCoAMAT[t],
  C12EnoylCoAMAT[t], C8EnoylCoAMAT[t], C6EnoylCoAMAT[t],
  C4EnoylCoAMAT[t], C10HydroxyacylCoAMAT[t], C16HydroxyacylCoAMAT[t],
  C14HydroxyacylCoAMAT[t], C12HydroxyacylCoAMAT[t], C8HydroxyacylCoAMAT[t],
  C6HydroxyacylCoAMAT[t], C4HydroxyacylCoAMAT[t], C4AcetoacylCoAMAT[t]],
vcrotC8 → CROT[sfcrotC8, Vcrot, KmcrotC8EnoylCoAMAT, KmcrotC16EnoylCoAMAT,
  KmcrotC14EnoylCoAMAT, KmcrotC12EnoylCoAMAT, KmcrotC10EnoylCoAMAT,
  KmcrotC6EnoylCoAMAT, KmcrotC4EnoylCoAMAT, KmcrotC8HydroxyacylCoAMAT,
  KmcrotC16HydroxyacylCoAMAT, KmcrotC14HydroxyacylCoAMAT,
  KmcrotC12HydroxyacylCoAMAT, KmcrotC10HydroxyacylCoAMAT,
  KmcrotC6HydroxyacylCoAMAT, KmcrotC4HydroxyacylCoAMAT, KicrotC4AcetoacylCoA,
  Keqcrot, C8EnoylCoAMAT[t], C16EnoylCoAMAT[t], C14EnoylCoAMAT[t],
  C12EnoylCoAMAT[t], C10EnoylCoAMAT[t], C6EnoylCoAMAT[t],
  C4EnoylCoAMAT[t], C8HydroxyacylCoAMAT[t], C16HydroxyacylCoAMAT[t],
  C14HydroxyacylCoAMAT[t], C12HydroxyacylCoAMAT[t], C10HydroxyacylCoAMAT[t],
  C6HydroxyacylCoAMAT[t], C4HydroxyacylCoAMAT[t], C4AcetoacylCoAMAT[t]],
vcrotC6 → CROT[sfcrotC6, Vcrot, KmcrotC6EnoylCoAMAT, KmcrotC16EnoylCoAMAT,
  KmcrotC14EnoylCoAMAT, KmcrotC12EnoylCoAMAT, KmcrotC10EnoylCoAMAT,
  KmcrotC8EnoylCoAMAT, KmcrotC4EnoylCoAMAT, KmcrotC6HydroxyacylCoAMAT,
  KmcrotC16HydroxyacylCoAMAT, KmcrotC14HydroxyacylCoAMAT,
  KmcrotC12HydroxyacylCoAMAT, KmcrotC10HydroxyacylCoAMAT,
  KmcrotC8HydroxyacylCoAMAT, KmcrotC4HydroxyacylCoAMAT, KicrotC4AcetoacylCoA,
  Keqcrot, C6EnoylCoAMAT[t], C16EnoylCoAMAT[t], C14EnoylCoAMAT[t],
  C12EnoylCoAMAT[t], C10EnoylCoAMAT[t], C8EnoylCoAMAT[t],
  C4EnoylCoAMAT[t], C6HydroxyacylCoAMAT[t], C16HydroxyacylCoAMAT[t],
  C14HydroxyacylCoAMAT[t], C12HydroxyacylCoAMAT[t], C10HydroxyacylCoAMAT[t],
  C8HydroxyacylCoAMAT[t], C4HydroxyacylCoAMAT[t], C4AcetoacylCoAMAT[t]],
vcrotC4 → CROT[sfcrotC4, Vcrot, KmcrotC4EnoylCoAMAT, KmcrotC16EnoylCoAMAT,
```

```
   KmcrotC14EnoylCoAMAT, KmcrotC12EnoylCoAMAT, KmcrotC10EnoylCoAMAT,
   KmcrotC8EnoylCoAMAT, KmcrotC6EnoylCoAMAT, KmcrotC4HydroxyacylCoAMAT,
   KmcrotC16HydroxyacylCoAMAT, KmcrotC14HydroxyacylCoAMAT,
   KmcrotC12HydroxyacylCoAMAT, KmcrotC10HydroxyacylCoAMAT,
   KmcrotC8HydroxyacylCoAMAT, KmcrotC6HydroxyacylCoAMAT, KicrotC4AcetoacylCoA,
   Keqcrot, C4EnoylCoAMAT[t], C16EnoylCoAMAT[t], C14EnoylCoAMAT[t],
   C12EnoylCoAMAT[t], C10EnoylCoAMAT[t], C8EnoylCoAMAT[t],
   C6EnoylCoAMAT[t], C4HydroxyacylCoAMAT[t], C16HydroxyacylCoAMAT[t],
   C14HydroxyacylCoAMAT[t], C12HydroxyacylCoAMAT[t], C10HydroxyacylCoAMAT[t],
   C8HydroxyacylCoAMAT[t], C6HydroxyacylCoAMAT[t], C4AcetoacylCoAMAT[t]],
 vmschadC16 → MSCHAD[sfmschadC16, Vmschad, KmmschadC16HydroxyacylCoAMAT,
   KmmschadC14HydroxyacylCoAMAT, KmmschadC12HydroxyacylCoAMAT,
   KmmschadC10HydroxyacylCoAMAT, KmmschadC8HydroxyacylCoAMAT,
   KmmschadC6HydroxyacylCoAMAT, KmmschadC4HydroxyacylCoAMAT,
   KmmschadNADMAT, KmmschadC16KetoacylCoAMAT, KmmschadC14KetoacylCoAMAT,
   KmmschadC12KetoacylCoAMAT, KmmschadC10KetoacylCoAMAT, KmmschadC8KetoacylCoAMAT,
   KmmschadC6KetoacylCoAMAT, KmmschadC4AcetoacylCoAMAT, KmmschadNADHMAT,
   Keqmschad, C16HydroxyacylCoAMAT[t], C14HydroxyacylCoAMAT[t],
   C12HydroxyacylCoAMAT[t], C10HydroxyacylCoAMAT[t], C8HydroxyacylCoAMAT[t],
   C6HydroxyacylCoAMAT[t], C4HydroxyacylCoAMAT[t], NADtMAT, C16KetoacylCoAMAT[t],
   C14KetoacylCoAMAT[t], C12KetoacylCoAMAT[t], C10KetoacylCoAMAT[t],
   C8KetoacylCoAMAT[t], C6KetoacylCoAMAT[t], C4AcetoacylCoAMAT[t], NADHMAT[t]],
 vmschadC14 → MSCHAD[sfmschadC14, Vmschad, KmmschadC14HydroxyacylCoAMAT,
   KmmschadC16HydroxyacylCoAMAT, KmmschadC12HydroxyacylCoAMAT,
   KmmschadC10HydroxyacylCoAMAT, KmmschadC8HydroxyacylCoAMAT,
   KmmschadC6HydroxyacylCoAMAT, KmmschadC4HydroxyacylCoAMAT,
   KmmschadNADMAT, KmmschadC14KetoacylCoAMAT, KmmschadC16KetoacylCoAMAT,
   KmmschadC12KetoacylCoAMAT, KmmschadC10KetoacylCoAMAT, KmmschadC8KetoacylCoAMAT,
   KmmschadC6KetoacylCoAMAT, KmmschadC4AcetoacylCoAMAT, KmmschadNADHMAT,
   Keqmschad, C14HydroxyacylCoAMAT[t], C16HydroxyacylCoAMAT[t],
   C12HydroxyacylCoAMAT[t], C10HydroxyacylCoAMAT[t], C8HydroxyacylCoAMAT[t],
   C6HydroxyacylCoAMAT[t], C4HydroxyacylCoAMAT[t], NADtMAT, C14KetoacylCoAMAT[t],
   C16KetoacylCoAMAT[t], C12KetoacylCoAMAT[t], C10KetoacylCoAMAT[t],
   C8KetoacylCoAMAT[t], C6KetoacylCoAMAT[t], C4AcetoacylCoAMAT[t], NADHMAT[t]],
 vmschadC12 → MSCHAD[sfmschadC12, Vmschad, KmmschadC12HydroxyacylCoAMAT,
   KmmschadC16HydroxyacylCoAMAT, KmmschadC14HydroxyacylCoAMAT,
   KmmschadC10HydroxyacylCoAMAT, KmmschadC8HydroxyacylCoAMAT,
   KmmschadC6HydroxyacylCoAMAT, KmmschadC4HydroxyacylCoAMAT,
   KmmschadNADMAT, KmmschadC12KetoacylCoAMAT, KmmschadC16KetoacylCoAMAT,
   KmmschadC14KetoacylCoAMAT, KmmschadC10KetoacylCoAMAT, KmmschadC8KetoacylCoAMAT,
   KmmschadC6KetoacylCoAMAT, KmmschadC4AcetoacylCoAMAT, KmmschadNADHMAT,
   Keqmschad, C12HydroxyacylCoAMAT[t], C16HydroxyacylCoAMAT[t],
   C14HydroxyacylCoAMAT[t], C10HydroxyacylCoAMAT[t], C8HydroxyacylCoAMAT[t],
   C6HydroxyacylCoAMAT[t], C4HydroxyacylCoAMAT[t], NADtMAT, C12KetoacylCoAMAT[t],
   C16KetoacylCoAMAT[t], C14KetoacylCoAMAT[t], C10KetoacylCoAMAT[t],
   C8KetoacylCoAMAT[t], C6KetoacylCoAMAT[t], C4AcetoacylCoAMAT[t], NADHMAT[t]],
 vmschadC10 → MSCHAD[sfmschadC10, Vmschad, KmmschadC10HydroxyacylCoAMAT,
   KmmschadC16HydroxyacylCoAMAT, KmmschadC14HydroxyacylCoAMAT,
   KmmschadC12HydroxyacylCoAMAT, KmmschadC8HydroxyacylCoAMAT,
   KmmschadC6HydroxyacylCoAMAT, KmmschadC4HydroxyacylCoAMAT,
```

```
   KmmschadNADMAT, KmmschadC10KetoacylCoAMAT, KmmschadC16KetoacylCoAMAT,
   KmmschadC14KetoacylCoAMAT, KmmschadC12KetoacylCoAMAT, KmmschadC8KetoacylCoAMAT,
   KmmschadC6KetoacylCoAMAT, KmmschadC4AcetoacylCoAMAT, KmmschadNADHMAT,
   Keqmschad, C10HydroxyacylCoAMAT[t], C16HydroxyacylCoAMAT[t],
   C14HydroxyacylCoAMAT[t], C12HydroxyacylCoAMAT[t], C8HydroxyacylCoAMAT[t],
   C6HydroxyacylCoAMAT[t], C4HydroxyacylCoAMAT[t], NADtMAT, C10KetoacylCoAMAT[t],
   C16KetoacylCoAMAT[t], C14KetoacylCoAMAT[t], C12KetoacylCoAMAT[t],
   C8KetoacylCoAMAT[t], C6KetoacylCoAMAT[t], C4AcetoacylCoAMAT[t], NADHMAT[t]],
vmschadC8 → MSCHAD[sfmschadC8, Vmschad, KmmschadC8HydroxyacylCoAMAT,
   KmmschadC16HydroxyacylCoAMAT, KmmschadC14HydroxyacylCoAMAT,
   KmmschadC12HydroxyacylCoAMAT, KmmschadC10HydroxyacylCoAMAT,
   KmmschadC6HydroxyacylCoAMAT, KmmschadC4HydroxyacylCoAMAT,
   KmmschadNADMAT, KmmschadC8KetoacylCoAMAT, KmmschadC16KetoacylCoAMAT,
   KmmschadC14KetoacylCoAMAT, KmmschadC12KetoacylCoAMAT, KmmschadC10KetoacylCoAMAT,
   KmmschadC6KetoacylCoAMAT, KmmschadC4AcetoacylCoAMAT, KmmschadNADHMAT,
   Keqmschad, C8HydroxyacylCoAMAT[t], C16HydroxyacylCoAMAT[t],
   C14HydroxyacylCoAMAT[t], C12HydroxyacylCoAMAT[t], C10HydroxyacylCoAMAT[t],
   C6HydroxyacylCoAMAT[t], C4HydroxyacylCoAMAT[t], NADtMAT, C8KetoacylCoAMAT[t],
   C16KetoacylCoAMAT[t], C14KetoacylCoAMAT[t], C12KetoacylCoAMAT[t],
   C10KetoacylCoAMAT[t], C6KetoacylCoAMAT[t], C4AcetoacylCoAMAT[t], NADHMAT[t]],
vmschadC6 → MSCHAD[sfmschadC6, Vmschad, KmmschadC6HydroxyacylCoAMAT,
   KmmschadC16HydroxyacylCoAMAT, KmmschadC14HydroxyacylCoAMAT,
   KmmschadC12HydroxyacylCoAMAT, KmmschadC10HydroxyacylCoAMAT,
   KmmschadC8HydroxyacylCoAMAT, KmmschadC4HydroxyacylCoAMAT,
   KmmschadNADMAT, KmmschadC6KetoacylCoAMAT, KmmschadC16KetoacylCoAMAT,
   KmmschadC14KetoacylCoAMAT, KmmschadC12KetoacylCoAMAT, KmmschadC10KetoacylCoAMAT,
   KmmschadC8KetoacylCoAMAT, KmmschadC4AcetoacylCoAMAT, KmmschadNADHMAT,
   Keqmschad, C6HydroxyacylCoAMAT[t], C16HydroxyacylCoAMAT[t],
   C14HydroxyacylCoAMAT[t], C12HydroxyacylCoAMAT[t], C10HydroxyacylCoAMAT[t],
   C8HydroxyacylCoAMAT[t], C4HydroxyacylCoAMAT[t], NADtMAT, C6KetoacylCoAMAT[t],
   C16KetoacylCoAMAT[t], C14KetoacylCoAMAT[t], C12KetoacylCoAMAT[t],
   C10KetoacylCoAMAT[t], C8KetoacylCoAMAT[t], C4AcetoacylCoAMAT[t], NADHMAT[t]],
vmschadC4 → MSCHAD[sfmschadC4, Vmschad, KmmschadC4HydroxyacylCoAMAT,
   KmmschadC16HydroxyacylCoAMAT, KmmschadC14HydroxyacylCoAMAT,
   KmmschadC12HydroxyacylCoAMAT, KmmschadC10HydroxyacylCoAMAT,
   KmmschadC8HydroxyacylCoAMAT, KmmschadC6HydroxyacylCoAMAT,
   KmmschadNADMAT, KmmschadC4AcetoacylCoAMAT, KmmschadC16KetoacylCoAMAT,
   KmmschadC14KetoacylCoAMAT, KmmschadC12KetoacylCoAMAT, KmmschadC10KetoacylCoAMAT,
   KmmschadC8KetoacylCoAMAT, KmmschadC6KetoacylCoAMAT, KmmschadNADHMAT,
   Keqmschad, C4HydroxyacylCoAMAT[t], C16HydroxyacylCoAMAT[t],
   C14HydroxyacylCoAMAT[t], C12HydroxyacylCoAMAT[t], C10HydroxyacylCoAMAT[t],
   C8HydroxyacylCoAMAT[t], C6HydroxyacylCoAMAT[t], NADtMAT, C4AcetoacylCoAMAT[t],
   C16KetoacylCoAMAT[t], C14KetoacylCoAMAT[t], C12KetoacylCoAMAT[t],
   C10KetoacylCoAMAT[t], C8KetoacylCoAMAT[t], C6KetoacylCoAMAT[t], NADHMAT[t]],
vmckatC16 → MCKATA[sfmckatC16, Vmckat, KmmckatC16KetoacylCoAMAT,
   KmmckatC14KetoacylCoAMAT, KmmckatC12KetoacylCoAMAT, KmmckatC10KetoacylCoAMAT,
   KmmckatC8KetoacylCoAMAT, KmmckatC6KetoacylCoAMAT, KmmckatC4AcetoacylCoAMAT,
   KmmckatCoAMAT, KmmckatC14AcylCoAMAT, KmmckatC16AcylCoAMAT, KmmckatC12AcylCoAMAT,
   KmmckatC10AcylCoAMAT, KmmckatC8AcylCoAMAT, KmmckatC6AcylCoAMAT,
   KmmckatC4AcylCoAMAT, KmmckatAcetylCoAMAT, Keqmckat, C16KetoacylCoAMAT[t],
```

```
    C14KetoacylCoAMAT[t], C12KetoacylCoAMAT[t], C10KetoacylCoAMAT[t],
    C8KetoacylCoAMAT[t], C6KetoacylCoAMAT[t], C4AcetoacylCoAMAT[t], CoAMAT,
    C14AcylCoAMAT[t], C16AcylCoAMAT[t], C12AcylCoAMAT[t], C10AcylCoAMAT[t],
    C8AcylCoAMAT[t], C6AcylCoAMAT[t], C4AcylCoAMAT[t], AcetylCoAMAT[t]],
  vmckatC14 → MCKATA[sfmckatC14, Vmckat, KmmckatC14KetoacylCoAMAT,
    KmmckatC16KetoacylCoAMAT, KmmckatC12KetoacylCoAMAT, KmmckatC10KetoacylCoAMAT,
    KmmckatC8KetoacylCoAMAT, KmmckatC6KetoacylCoAMAT, KmmckatC4AcetoacylCoAMAT,
    KmmckatCoAMAT, KmmckatC12AcylCoAMAT, KmmckatC16AcylCoAMAT, KmmckatC14AcylCoAMAT,
    KmmckatC10AcylCoAMAT, KmmckatC8AcylCoAMAT, KmmckatC6AcylCoAMAT,
    KmmckatC4AcylCoAMAT, KmmckatAcetylCoAMAT, Keqmckat, C14KetoacylCoAMAT[t],
    C16KetoacylCoAMAT[t], C12KetoacylCoAMAT[t], C10KetoacylCoAMAT[t],
    C8KetoacylCoAMAT[t], C6KetoacylCoAMAT[t], C4AcetoacylCoAMAT[t], CoAMAT,
    C12AcylCoAMAT[t], C16AcylCoAMAT[t], C14AcylCoAMAT[t], C10AcylCoAMAT[t],
    C8AcylCoAMAT[t], C6AcylCoAMAT[t], C4AcylCoAMAT[t], AcetylCoAMAT[t]],
  vmckatC12 → MCKATA[sfmckatC12, Vmckat, KmmckatC12KetoacylCoAMAT,
    KmmckatC16KetoacylCoAMAT, KmmckatC14KetoacylCoAMAT, KmmckatC10KetoacylCoAMAT,
    KmmckatC8KetoacylCoAMAT, KmmckatC6KetoacylCoAMAT, KmmckatC4AcetoacylCoAMAT,
    KmmckatCoAMAT, KmmckatC10AcylCoAMAT, KmmckatC16AcylCoAMAT, KmmckatC14AcylCoAMAT,
    KmmckatC12AcylCoAMAT, KmmckatC8AcylCoAMAT, KmmckatC6AcylCoAMAT,
    KmmckatC4AcylCoAMAT, KmmckatAcetylCoAMAT, Keqmckat, C12KetoacylCoAMAT[t],
    C16KetoacylCoAMAT[t], C14KetoacylCoAMAT[t], C10KetoacylCoAMAT[t],
    C8KetoacylCoAMAT[t], C6KetoacylCoAMAT[t], C4AcetoacylCoAMAT[t], CoAMAT,
    C10AcylCoAMAT[t], C16AcylCoAMAT[t], C14AcylCoAMAT[t], C12AcylCoAMAT[t],
    C8AcylCoAMAT[t], C6AcylCoAMAT[t], C4AcylCoAMAT[t], AcetylCoAMAT[t]],
  vmckatC10 → MCKATA[sfmckatC10, Vmckat, KmmckatC10KetoacylCoAMAT,
    KmmckatC16KetoacylCoAMAT, KmmckatC14KetoacylCoAMAT, KmmckatC12KetoacylCoAMAT,
    KmmckatC8KetoacylCoAMAT, KmmckatC6KetoacylCoAMAT, KmmckatC4AcetoacylCoAMAT,
    KmmckatCoAMAT, KmmckatC8AcylCoAMAT, KmmckatC16AcylCoAMAT, KmmckatC14AcylCoAMAT,
    KmmckatC12AcylCoAMAT, KmmckatC10AcylCoAMAT, KmmckatC6AcylCoAMAT,
    KmmckatC4AcylCoAMAT, KmmckatAcetylCoAMAT, Keqmckat, C10KetoacylCoAMAT[t],
    C16KetoacylCoAMAT[t], C14KetoacylCoAMAT[t], C12KetoacylCoAMAT[t],
    C8KetoacylCoAMAT[t], C6KetoacylCoAMAT[t], C4AcetoacylCoAMAT[t], CoAMAT,
    C8AcylCoAMAT[t], C16AcylCoAMAT[t], C14AcylCoAMAT[t], C12AcylCoAMAT[t],
    C10AcylCoAMAT[t], C6AcylCoAMAT[t], C4AcylCoAMAT[t], AcetylCoAMAT[t]],
  vmckatC8 → MCKATA[sfmckatC8, Vmckat, KmmckatC8KetoacylCoAMAT,
    KmmckatC16KetoacylCoAMAT, KmmckatC14KetoacylCoAMAT, KmmckatC12KetoacylCoAMAT,
    KmmckatC10KetoacylCoAMAT, KmmckatC6KetoacylCoAMAT, KmmckatC4AcetoacylCoAMAT,
    KmmckatCoAMAT, KmmckatC6AcylCoAMAT, KmmckatC16AcylCoAMAT, KmmckatC14AcylCoAMAT,
    KmmckatC12AcylCoAMAT, KmmckatC10AcylCoAMAT, KmmckatC8AcylCoAMAT,
    KmmckatC4AcylCoAMAT, KmmckatAcetylCoAMAT, Keqmckat, C8KetoacylCoAMAT[t],
    C16KetoacylCoAMAT[t], C14KetoacylCoAMAT[t], C12KetoacylCoAMAT[t],
    C10KetoacylCoAMAT[t], C6KetoacylCoAMAT[t], C4AcetoacylCoAMAT[t], CoAMAT,
    C6AcylCoAMAT[t], C16AcylCoAMAT[t], C14AcylCoAMAT[t], C12AcylCoAMAT[t],
    C10AcylCoAMAT[t], C8AcylCoAMAT[t], C4AcylCoAMAT[t], AcetylCoAMAT[t]],
  vmckatC6 → MCKATA[sfmckatC6, Vmckat, KmmckatC6KetoacylCoAMAT,
    KmmckatC16KetoacylCoAMAT, KmmckatC14KetoacylCoAMAT, KmmckatC12KetoacylCoAMAT,
    KmmckatC10KetoacylCoAMAT, KmmckatC8KetoacylCoAMAT, KmmckatC4AcetoacylCoAMAT,
    KmmckatCoAMAT, KmmckatC4AcylCoAMAT, KmmckatC16AcylCoAMAT, KmmckatC14AcylCoAMAT,
    KmmckatC12AcylCoAMAT, KmmckatC10AcylCoAMAT, KmmckatC8AcylCoAMAT,
    KmmckatC6AcylCoAMAT, KmmckatAcetylCoAMAT, Keqmckat, C6KetoacylCoAMAT[t],
```

```
    C16KetoacylCoAMAT[t], C14KetoacylCoAMAT[t], C12KetoacylCoAMAT[t],
    C10KetoacylCoAMAT[t], C8KetoacylCoAMAT[t], C4AcetoacylCoAMAT[t], CoAMAT,
    C4AcylCoAMAT[t], C16AcylCoAMAT[t], C14AcylCoAMAT[t], C12AcylCoAMAT[t],
    C10AcylCoAMAT[t], C8AcylCoAMAT[t], C6AcylCoAMAT[t], AcetylCoAMAT[t]],
  vmckatC4 → MCKATB[sfmckatC4, Vmckat, KmmckatC4AcetoacylCoAMAT,
    KmmckatC16KetoacylCoAMAT, KmmckatC14KetoacylCoAMAT, KmmckatC12KetoacylCoAMAT,
    KmmckatC10KetoacylCoAMAT, KmmckatC8KetoacylCoAMAT, KmmckatC6KetoacylCoAMAT,
    KmmckatCoAMAT, KmmckatC4AcylCoAMAT, KmmckatC16AcylCoAMAT, KmmckatC14AcylCoAMAT,
    KmmckatC12AcylCoAMAT, KmmckatC10AcylCoAMAT, KmmckatC8AcylCoAMAT,
    KmmckatC6AcylCoAMAT, KmmckatAcetylCoAMAT, Keqmckat, C4AcetoacylCoAMAT[t],
    C16KetoacylCoAMAT[t], C14KetoacylCoAMAT[t], C12KetoacylCoAMAT[t],
    C10KetoacylCoAMAT[t], C8KetoacylCoAMAT[t], C6KetoacylCoAMAT[t], CoAMAT,
    C4AcylCoAMAT[t], C16AcylCoAMAT[t], C14AcylCoAMAT[t], C12AcylCoAMAT[t],
    C10AcylCoAMAT[t], C8AcylCoAMAT[t], C6AcylCoAMAT[t], AcetylCoAMAT[t]],
  vmtpC16 → MTP[sfmtpC16, Vmtp, KmmtpC16EnoylCoAMAT, KmmtpC14EnoylCoAMAT,
    KmmtpC12EnoylCoAMAT, KmmtpC10EnoylCoAMAT, KmmtpC8EnoylCoAMAT,
    KmmtpNADMAT, KmmtpCoAMAT, KmmtpC14AcylCoAMAT, KmmtpC16AcylCoAMAT,
    KmmtpC12AcylCoAMAT, KmmtpC10AcylCoAMAT, KmmtpC8AcylCoAMAT,
    KmmtpC6AcylCoAMAT, KmmtpNADHMAT, KmmtpAcetylCoAMAT, KicrotC4AcetoacylCoA,
    Keqmtp, C16EnoylCoAMAT[t], C14EnoylCoAMAT[t], C12EnoylCoAMAT[t],
    C10EnoylCoAMAT[t], C8EnoylCoAMAT[t], NADtMAT, CoAMAT, C14AcylCoAMAT[t],
    C16AcylCoAMAT[t], C12AcylCoAMAT[t], C10AcylCoAMAT[t], C8AcylCoAMAT[t],
    C6AcylCoAMAT[t], NADHMAT[t], AcetylCoAMAT[t], C4AcetoacylCoAMAT[t]],
  vmtpC14 → MTP[sfmtpC14, Vmtp, KmmtpC14EnoylCoAMAT, KmmtpC16EnoylCoAMAT,
    KmmtpC12EnoylCoAMAT, KmmtpC10EnoylCoAMAT, KmmtpC8EnoylCoAMAT,
    KmmtpNADMAT, KmmtpCoAMAT, KmmtpC12AcylCoAMAT, KmmtpC16AcylCoAMAT,
    KmmtpC14AcylCoAMAT, KmmtpC10AcylCoAMAT, KmmtpC8AcylCoAMAT,
    KmmtpC6AcylCoAMAT, KmmtpNADHMAT, KmmtpAcetylCoAMAT, KicrotC4AcetoacylCoA,
    Keqmtp, C14EnoylCoAMAT[t], C16EnoylCoAMAT[t], C12EnoylCoAMAT[t],
    C10EnoylCoAMAT[t], C8EnoylCoAMAT[t], NADtMAT, CoAMAT, C12AcylCoAMAT[t],
    C16AcylCoAMAT[t], C14AcylCoAMAT[t], C10AcylCoAMAT[t], C8AcylCoAMAT[t],
    C6AcylCoAMAT[t], NADHMAT[t], AcetylCoAMAT[t], C4AcetoacylCoAMAT[t]],
  vmtpC12 → MTP[sfmtpC12, Vmtp, KmmtpC12EnoylCoAMAT, KmmtpC16EnoylCoAMAT,
    KmmtpC14EnoylCoAMAT, KmmtpC10EnoylCoAMAT, KmmtpC8EnoylCoAMAT,
    KmmtpNADMAT, KmmtpCoAMAT, KmmtpC10AcylCoAMAT, KmmtpC16AcylCoAMAT,
    KmmtpC14AcylCoAMAT, KmmtpC12AcylCoAMAT, KmmtpC8AcylCoAMAT,
    KmmtpC6AcylCoAMAT, KmmtpNADHMAT, KmmtpAcetylCoAMAT, KicrotC4AcetoacylCoA,
    Keqmtp, C12EnoylCoAMAT[t], C16EnoylCoAMAT[t], C14EnoylCoAMAT[t],
    C10EnoylCoAMAT[t], C8EnoylCoAMAT[t], NADtMAT, CoAMAT, C10AcylCoAMAT[t],
    C16AcylCoAMAT[t], C14AcylCoAMAT[t], C12AcylCoAMAT[t], C8AcylCoAMAT[t],
    C6AcylCoAMAT[t], NADHMAT[t], AcetylCoAMAT[t], C4AcetoacylCoAMAT[t]],
  vmtpC10 → MTP[sfmtpC10, Vmtp, KmmtpC10EnoylCoAMAT, KmmtpC16EnoylCoAMAT,
    KmmtpC14EnoylCoAMAT, KmmtpC12EnoylCoAMAT, KmmtpC8EnoylCoAMAT,
    KmmtpNADMAT, KmmtpCoAMAT, KmmtpC8AcylCoAMAT, KmmtpC16AcylCoAMAT,
    KmmtpC14AcylCoAMAT, KmmtpC12AcylCoAMAT, KmmtpC10AcylCoAMAT,
    KmmtpC6AcylCoAMAT, KmmtpNADHMAT, KmmtpAcetylCoAMAT, KicrotC4AcetoacylCoA,
    Keqmtp, C10EnoylCoAMAT[t], C16EnoylCoAMAT[t], C14EnoylCoAMAT[t],
    C12EnoylCoAMAT[t], C8EnoylCoAMAT[t], NADtMAT, CoAMAT, C8AcylCoAMAT[t],
    C16AcylCoAMAT[t], C14AcylCoAMAT[t], C12AcylCoAMAT[t], C10AcylCoAMAT[t],
    C6AcylCoAMAT[t], NADHMAT[t], AcetylCoAMAT[t], C4AcetoacylCoAMAT[t]],
```

```
   vmtpC8 → MTP[sfmtpC8, Vmtp, KmmtpC8EnoylCoAMAT, KmmtpC16EnoylCoAMAT,
     KmmtpC14EnoylCoAMAT, KmmtpC12EnoylCoAMAT, KmmtpC10EnoylCoAMAT,
     KmmtpNADMAT, KmmtpCoAMAT, KmmtpC6AcylCoAMAT, KmmtpC16AcylCoAMAT,
     KmmtpC14AcylCoAMAT, KmmtpC12AcylCoAMAT, KmmtpC10AcylCoAMAT,
     KmmtpC8AcylCoAMAT, KmmtpNADHMAT, KmmtpAcetylCoAMAT, KicrotC4AcetoacylCoA,
     Keqmtp, C8EnoylCoAMAT[t], C16EnoylCoAMAT[t], C14EnoylCoAMAT[t],
     C12EnoylCoAMAT[t], C10EnoylCoAMAT[t], NADtMAT, CoAMAT, C6AcylCoAMAT[t],
     C16AcylCoAMAT[t], C14AcylCoAMAT[t], C12AcylCoAMAT[t], C10AcylCoAMAT[t],
     C8AcylCoAMAT[t], NADHMAT[t], AcetylCoAMAT[t], C4AcetoacylCoAMAT[t]],
   vacesink → RES[Ksacesink, AcetylCoAMAT[t], K1acesink],
   vfadhsink → RES[Ksfadhsink, FADHMAT[t], K1fadhsink],
   vnadhsink → RES[Ksnadhsink, NADHMAT[t], K1nadhsink]};

CoAMATX =
  {CoAMAT → CoAMATt - C16AcylCoAMAT[t] - C16EnoylCoAMAT[t] - C16HydroxyacylCoAMAT[t] -
     C16KetoacylCoAMAT[t] - C14AcylCoAMAT[t] - C14EnoylCoAMAT[t] -
     C14HydroxyacylCoAMAT[t] - C14KetoacylCoAMAT[t] - C12AcylCoAMAT[t] -
     C12EnoylCoAMAT[t] - C12HydroxyacylCoAMAT[t] - C12KetoacylCoAMAT[t] -
     C10AcylCoAMAT[t] - C10EnoylCoAMAT[t] - C10HydroxyacylCoAMAT[t] -
     C10KetoacylCoAMAT[t] - C8AcylCoAMAT[t] - C8EnoylCoAMAT[t] -
     C8HydroxyacylCoAMAT[t] - C8KetoacylCoAMAT[t] - C6AcylCoAMAT[t] - C6EnoylCoAMAT[t] -
     C6HydroxyacylCoAMAT[t] - C6KetoacylCoAMAT[t] - C4AcylCoAMAT[t] - C4EnoylCoAMAT[t] -
     C4HydroxyacylCoAMAT[t] - C4AcetoacylCoAMAT[t] - AcetylCoAMAT[t]};

Parm = {
   sfcpt1C16 → 1, Kmcpt1C16AcylCoACYT → 13.8,
   Kmcpt1CarCYT → 500, Kmcpt1C16AcylCarCYT → 136, Kmcpt1CoACYT → 40.7,
   Kicpt1MalCoACYT → 0.2, Keqcpt1 → 0.45,  ncpt1 → 2.4799,
   KmcactC16AcylCarCYT → 15, KmcactC14AcylCarCYT → 15, KmcactC12AcylCarCYT → 15,
   KmcactC10AcylCarCYT → 15, KmcactC8AcylCarCYT → 15, KmcactC6AcylCarCYT → 15,
   KmcactC4AcylCarCYT → 15, KmcactCarMAT → 130, KmcactC16AcylCarMAT → 15,
   KmcactC14AcylCarMAT → 15, KmcactC12AcylCarMAT → 15, KmcactC10AcylCarMAT → 15,
   KmcactC8AcylCarMAT → 15, KmcactC6AcylCarMAT → 15, KmcactC4AcylCarMAT → 15,
   KmcactCarCYT → 130, KicactC16AcylCarCYT → 56, KicactC14AcylCarCYT → 56,
   KicactC12AcylCarCYT → 56, KicactC10AcylCarCYT → 56, KicactC8AcylCarCYT → 56,
   KicactC6AcylCarCYT → 56, KicactC4AcylCarCYT → 56, KicactCarCYT → 200, Keqcact → 1,
   sfcpt2C16 → 1.0, sfcpt2C14 → 1, sfcpt2C12 → 0.42, sfcpt2C10 → 0.39, sfcpt2C8 → 0.20,
   sfcpt2C6 →  0.02, sfcpt2C4 → 0.002, Kmcpt2C16AcylCarMAT → 51, Kmcpt2C14AcylCarMAT → 51,
   Kmcpt2C12AcylCarMAT → 51, Kmcpt2C10AcylCarMAT → 51, Kmcpt2C8AcylCarMAT → 51,
   Kmcpt2C6AcylCarMAT → 51, Kmcpt2C4AcylCarMAT → 51, Kmcpt2CoAMAT → 30,
   Kmcpt2C16AcylCoAMAT → 2.75, Kmcpt2C14AcylCoAMAT → 19.1, Kmcpt2C12AcylCoAMAT → 15.0,
   Kmcpt2C10AcylCoAMAT → 326, Kmcpt2C8AcylCoAMAT → 140 235, Kmcpt2C6AcylCoAMAT → 79.4,
   Kmcpt2C4AcylCoAMAT → 28 116, Kmcpt2CarMAT → 350, Keqcpt2 → 2.22,
   sfvlcadC16 → 0.8, sfvlcadC14 → 0.46, sfvlcadC12 → 0.08, KmvlcadC16AcylCoAMAT → 6.5,
   KmvlcadC14AcylCoAMAT → 8, KmvlcadC12AcylCoAMAT → 1, KmvlcadFAD → 0.12,
   KmvlcadC16EnoylCoAMAT → 1.08, KmvlcadC14EnoylCoAMAT → 1.08,
   KmvlcadC12EnoylCoAMAT → 1.08, KmvlcadFADH → 24.2, Keqvlcad → 6,
   sflcadC16 → 0.68, sflcadC14 → 0.69, sflcadC12 → 0.8, sflcadC10 → 0.59,
   sflcadC8 → 0.72, KmlcadC16AcylCoAMAT → 2.52, KmlcadC14AcylCoAMAT → 46.3,
   KmlcadC12AcylCoAMAT → 35.3, KmlcadC10AcylCoAMAT → 23.4, KmlcadC8AcylCoAMAT → 123,
```

```
KmlcadFAD → 0.12, KmlcadC16EnoylCoAMAT → 1.08, KmlcadC14EnoylCoAMAT → 1.08,
KmlcadC12EnoylCoAMAT → 1.08, KmlcadC10EnoylCoAMAT → 1.08,
KmlcadC8EnoylCoAMAT → 1.08, KmlcadFADH → 24.2, Keqlcad → 6,
sfmcadC12 → 0.15, sfmcadC10 → 1, sfmcadC8 → 0.89, sfmcadC6 → 0.73,
sfmcadC4 → 1, KmmcadC12AcylCoAMAT → 63.6, KmmcadC10AcylCoAMAT → 1,
KmmcadC8AcylCoAMAT → 1, KmmcadC6AcylCoAMAT → 4.1, KmmcadC4AcylCoAMAT → 135,
KmmcadFAD → 0.12, KmmcadC12EnoylCoAMAT → 1.08, KmmcadC10EnoylCoAMAT → 1.08,
KmmcadC8EnoylCoAMAT → 1.08, KmmcadC6EnoylCoAMAT → 1.08,
KmmcadC4EnoylCoAMAT → 1.08, KmmcadFADH → 24.2, Keqmcad → 6,
sfscadC6 → 1, sfscadC4 → 1, KmscadC6AcylCoAMAT → 285,
KmscadC4AcylCoAMAT → 5.78, KmscadFAD → 0.12, KmscadC6EnoylCoAMAT → 1.08,
KmscadC4EnoylCoAMAT → 1.08, KmscadFADH → 24.2, Keqscad → 6,
sfcrotC16 → 0.13, sfcrotC14 → 0.2, sfcrotC12 → 0.25, sfcrotC10 → 0.33,
sfcrotC8 → 0.58, sfcrotC6 → 0.83, sfcrotC4 → 1, KmcrotC16EnoylCoAMAT → 150,
KmcrotC14EnoylCoAMAT → 100, KmcrotC12EnoylCoAMAT → 25, KmcrotC10EnoylCoAMAT → 25,
KmcrotC8EnoylCoAMAT → 25, KmcrotC6EnoylCoAMAT → 25, KmcrotC4EnoylCoAMAT → 40,
KmcrotC16HydroxyacylCoAMAT → 45, KmcrotC14HydroxyacylCoAMAT → 45,
KmcrotC12HydroxyacylCoAMAT → 45, KmcrotC10HydroxyacylCoAMAT → 45,
KmcrotC8HydroxyacylCoAMAT → 45, KmcrotC6HydroxyacylCoAMAT → 45,
KmcrotC4HydroxyacylCoAMAT → 45, KicrotC4AcetoacylCoA → 1.6, Keqcrot → 3.13,
sfmschadC16 → 0.6, sfmschadC14 → 0.5, sfmschadC12 → 0.43,
sfmschadC10 → 0.64, sfmschadC8 → 0.89, sfmschadC6 → 1, sfmschadC4 → 0.67,
KmmschadC16HydroxyacylCoAMAT → 1.5, KmmschadC14HydroxyacylCoAMAT → 1.8,
KmmschadC12HydroxyacylCoAMAT → 3.7, KmmschadC10HydroxyacylCoAMAT → 8.8,
KmmschadC8HydroxyacylCoAMAT → 16.3, KmmschadC6HydroxyacylCoAMAT → 28.6,
KmmschadC4HydroxyacylCoAMAT → 69.9, KmmschadNADMAT → 58.5,
KmmschadC16KetoacylCoAMAT → 1.4, KmmschadC14KetoacylCoAMAT → 1.4,
KmmschadC12KetoacylCoAMAT → 1.6, KmmschadC10KetoacylCoAMAT → 2.3,
KmmschadC8KetoacylCoAMAT → 4.1, KmmschadC6KetoacylCoAMAT → 5.8,
KmmschadC4AcetoacylCoAMAT → 16.9, KmmschadNADHMAT → 5.4, Keqmschad → 2.17 * 10^-4,
sfmckatC16 → 0, sfmckatC14 → 0.2, sfmckatC12 → 0.38, sfmckatC10 → 0.65,
sfmckatC8 → 0.81, sfmckatC6 → 1, sfmckatC4 → 0.49, KmmckatC16KetoacylCoAMAT → 1.1,
KmmckatC14KetoacylCoAMAT → 1.2, KmmckatC12KetoacylCoAMAT → 1.3,
KmmckatC10KetoacylCoAMAT → 2.1, KmmckatC8KetoacylCoAMAT → 3.2,
KmmckatC6KetoacylCoAMAT → 0.67, KmmckatC4AcetoacylCoAMAT → 1.24,
KmmckatCoAMAT → 26.6, KmmckatC14AcylCoAMAT → 13.83, KmmckatC16AcylCoAMAT → 13.83,
KmmckatC12AcylCoAMAT → 13.83, KmmckatC10AcylCoAMAT → 13.83,
KmmckatC8AcylCoAMAT → 13.83, KmmckatC6AcylCoAMAT → 13.83,
KmmckatC4AcylCoAMAT → 13.83, KmmckatAcetylCoAMAT → 30, Keqmckat → 1051,
sfmtpC16 → 1, sfmtpC14 → 0.9, sfmtpC12 → 0.81, sfmtpC10 → 0.73, sfmtpC8 → 0.34,
KmmtpC16EnoylCoAMAT → 25, KmmtpC14EnoylCoAMAT → 25, KmmtpC12EnoylCoAMAT → 25,
KmmtpC10EnoylCoAMAT → 25, KmmtpC8EnoylCoAMAT → 25, KmmtpNADMAT → 60,
KmmtpCoAMAT → 30, KmmtpC14AcylCoAMAT → 13.83, KmmtpC16AcylCoAMAT → 13.83,
KmmtpC12AcylCoAMAT → 13.83, KmmtpC10AcylCoAMAT → 13.83, KmmtpC8AcylCoAMAT → 13.83,
KmmtpC6AcylCoAMAT → 13.83, KmmtpNADHMAT → 50, KmmtpAcetylCoAMAT → 30, Keqmtp → 0.71,
Ksacesink → 6 000 000, K1acesink → 70, Ksfadhsink → 6 000 000,
K1fadhsink → 0.46, Ksnadhsink → 6 000 000, K1nadhsink → 16, Kmdummy → 1 * 10^20,
C16AcylCoACYT → 25., CarCYT → 200., CoACYT → 140, MalCoACYT → 0.2,
CarMAT → 950, FADtMAT → 0.77, NADtMAT → 250, CoAMATt → 5000,
VCYT → 2.2 * 10^-6, VMAT → 1.8 * 10^-6};
```

```
Parm2 = {Vmckat → 0.012, Vmcad → 0.032, Vscad → 0.019,
   Vvlcad → 0.005, Vcpt1 → 0.008, Vcpt2 → 0.005, Vcrot → 3, Vmschad → 0.09,
   Vmtp → 0.116, Vfcact → 0.42, Vrcact → 0.42, Vlcad → 0.035};

InitialConditions = {
   C16AcylCarCYT[0] == 0, C16AcylCarMAT[0] == 0, C16AcylCoAMAT[0] == 0,
   C16EnoylCoAMAT[0] == 0, C16HydroxyacylCoAMAT[0] == 0, C16KetoacylCoAMAT[0] == 0,
   C14AcylCarCYT[0] == 0.0, C14AcylCarMAT[0] == 0, C14AcylCoAMAT[0] == 0,
   C14EnoylCoAMAT[0] == 0, C14HydroxyacylCoAMAT[0] == 0, C14KetoacylCoAMAT[0] == 0,
   C12AcylCarCYT[0] == 0.0, C12AcylCarMAT[0] == 0, C12AcylCoAMAT[0] == 0,
   C12EnoylCoAMAT[0] == 0, C12HydroxyacylCoAMAT[0] == 0, C12KetoacylCoAMAT[0] == 0,
   C10AcylCarCYT[0] == 0.0, C10AcylCarMAT[0] == 0, C10AcylCoAMAT[0] == 0,
   C10EnoylCoAMAT[0] == 0, C10HydroxyacylCoAMAT[0] == 0, C10KetoacylCoAMAT[0] == 0,
   C8AcylCarCYT[0] == 0.0, C8AcylCarMAT[0] == 0, C8AcylCoAMAT[0] == 0,
   C8EnoylCoAMAT[0] == 0, C8HydroxyacylCoAMAT[0] == 0, C8KetoacylCoAMAT[0] == 0,
   C6AcylCarCYT[0] == 0.0, C6AcylCarMAT[0] == 0, C6AcylCoAMAT[0] == 0,
   C6EnoylCoAMAT[0] == 0, C6HydroxyacylCoAMAT[0] == 0, C6KetoacylCoAMAT[0] == 0,
   C4AcylCarCYT[0] == 0.0, C4AcylCarMAT[0] == 0, C4AcylCoAMAT[0] == 0,
   C4EnoylCoAMAT[0] == 0, C4HydroxyacylCoAMAT[0] == 0, C4AcetoacylCoAMAT[0] == 0,
   AcetylCoAMAT[0] == 70, FADHMAT[0] == 0.46, NADHMAT[0] == 16};

Vars = {
   C16AcylCarCYT, C16AcylCarMAT, C16AcylCoAMAT,
   C16EnoylCoAMAT, C16HydroxyacylCoAMAT, C16KetoacylCoAMAT,
   C14AcylCarCYT, C14AcylCarMAT, C14AcylCoAMAT, C14EnoylCoAMAT,
   C14HydroxyacylCoAMAT, C14KetoacylCoAMAT,
   C12AcylCarCYT, C12AcylCarMAT, C12AcylCoAMAT, C12EnoylCoAMAT,
   C12HydroxyacylCoAMAT, C12KetoacylCoAMAT,
   C10AcylCarCYT, C10AcylCarMAT, C10AcylCoAMAT, C10EnoylCoAMAT,
   C10HydroxyacylCoAMAT, C10KetoacylCoAMAT,
   C8AcylCarCYT, C8AcylCarMAT, C8AcylCoAMAT, C8EnoylCoAMAT,
   C8HydroxyacylCoAMAT, C8KetoacylCoAMAT,
   C6AcylCarCYT, C6AcylCarMAT, C6AcylCoAMAT, C6EnoylCoAMAT,
   C6HydroxyacylCoAMAT, C6KetoacylCoAMAT,
   C4AcylCarCYT, C4AcylCarMAT, C4AcylCoAMAT, C4EnoylCoAMAT,
   C4HydroxyacylCoAMAT, C4AcetoacylCoAMAT,
   AcetylCoAMAT, FADHMAT, NADHMAT};

TableForm[Odes];
TableForm[RateEqs];
TableForm[Odes /. RateEqs /. CoAMATX /. Parm /. Parm2];
TableForm[RateEqs /. Parm /. Parm2];
TableForm[InitialConditions];


tsol = NDSolve[Join[Odes /. RateEqs /. CoAMATX /. Parm /. Parm2, InitialConditions],
   Vars, {t, 0, 1000}];
```

Flux Control Analysis

```
ParallelTable[{Vars[[i]][t], (Vars[[i]][1000] /. tsol)[[1]]}, {i, 1, Length[Vars]}];
ss1 = FindRoot[
   Table[Odes[[i, 2]] == 0, {i, 1, Length[Odes]}] /. RateEqs /. CoAMATX /. Parm /. Parm2,
   %];
fluxes1 = RateEqs /. CoAMATX /. Parm /. Parm2 /. ss1;
Metabolites =
  TableForm[ParallelTable[{ss1[[i]][[1]], ss1[[i]][[2]]}, {i, 1, Length[ss1]}]];
Flux = TableForm[ParallelTable[{fluxes1[[i]][[1]], fluxes1[[i]][[2]]},
    {i, 1, Length[fluxes1]}]];

 FCCMuscle[zstart_, dz_, zend_] := Monitor[Module[{vector2},
    k = {0.1, 0.5, 1.0, 2.0, 3.0, 4.0, 5.0, 6.0, 7.0, 8.0, 9.0, 10.0};
    Uptakefluxesallanimals = {};
    FCCvector = {};
    a = newvmaxes[[1]];
    Alldeltacalculations = {};
    AllFCCcalculations = {};
    For[z = zstart, z ≤ zend,

     vectorvmaxes = {0};
     For[i = 2, i ≤ 12, i++,
      AppendTo[vectorvmaxes, newvmaxes[[i, z]]];
     ];

     FCCvector = {};
     FCCmatrix = {};
     Uptakefluxesallanimals = {};

     For[x = 1, x ≤ Length[vectorvmaxes], x++,
      delta = 0.000001;
      vector2 = vectorvmaxes;
      cd = N[vector2[[x]] * (1 + delta), 30];
      vector2[[x]] = cd;

      Parm2replaced =
       {Vmckat → vector2[[2]], Vmcad → vector2[[3]], Vscad → vector2[[4]],
        Vvlcad → vector2[[5]], Vcpt1 → vector2[[6]], Vcpt2 → vector2[[7]],
        Vcrot → vector2[[8]], Vmschad → vector2[[9]], Vmtp → vector2[[10]],
        Vfcact → vector2[[11]], Vrcact → vector2[[11]], Vlcad → vector2[[12]]};

      tsol0 = ParallelTable[
        NDSolve[Join[Odes /. RateEqs /. CoAMATX /. ReplacePart[Parm, Position[Parm,
                C16AcylCoACYT][[1, 1]] → C16AcylCoACYT → k[[j]]] /. Parm2replaced,
          ReplacePart[InitialConditions, {ii_, 2} :> ss1[[ ii, 2]]]], Vars,
         {t, 0, 1000}], {j, 1, Length[k]}];
      ssp0 = Table[FindRoot[Table[Odes[[i, 2]] == 0, {i, 1, Length[Odes]}] /. RateEqs /.
            CoAMATX /. ReplacePart[Parm, Position[Parm, C16AcylCoACYT][[1, 1]] →
```

```
           C16AcylCoACYT → k[[j]]] /. Parm2replaced,
         ParallelTable[{Vars[[i]][t], (Vars[[i]][1000] /. tsol0[[j]])[[1]]},
          {i, 1, Length[Vars]}]], {j, 1, Length[k]}];

    Jk = ParallelTable[RateEqs[[All, 2]] /. CoAMATX /. ReplacePart[Parm,
           Position[Parm, C16AcylCoACYT][[1, 1]] → C16AcylCoACYT → k[[j]]] /.
          Parm2replaced /. ssp0[[j]], {j, 1, Length[k]}];
    J0 = TableForm[ParallelTable[Jk[[j, m]], {j, 1, Length[k]},
        {m, 1, Length[RateEqs]}]];

    Jknames = fluxes1[[All, 1]];
    Metk = ParallelTable[ssp0[[j, All, 2]], {j, 1, Length[k]}];
    Met0 = TableForm[Metk];
    Metknames = ss1[[All, 1]];
    Jkmat = ConstantArray[0, {Length[Jk[[1]]], Length [Jk[[All, 1]]] + 1}];

    For[i = 1, i ≤ Length[Jk[[1]]], i++,
     Jkmat[[i, 1]] = Jknames[[i]];
     For[j = 1, j ≤ Length[Jk], j++,
      Jkmat[[i, j + 1]] = Jk[[j, i]]
     ]
    ];

    AppendTo[Uptakefluxesallanimals, Jkmat[[1]]];
   ];
   For[xy = 2, xy ≤ Length[Uptakefluxesallanimals], xy++,
    For[yz = 2, yz ≤ Length[Uptakefluxesallanimals[[1]]], yz++,

     Jk1 = N[Uptakefluxesallanimals[[xy, yz]], 30];
     Jk0 = N[Uptakefluxesallanimals[[1, yz]], 30];

     DJk = Jk1 - Jk0;
     DVmax = N[vectorvmaxes[[xy]] * delta, 30];
     FCC = N[(DJk * vectorvmaxes[[xy]]) / (DVmax * Jk0), 30];
     AppendTo[FCCvector, FCC];
    ];
    AppendTo[FCCmatrix, FCCvector];
    FCCvector = {};
   ];

   string1 = "C:\\FCC_";
   string2 = ToString[z - 1];
   string3 = ToString[newvmaxes[[1, z]]];
   string4 = ".xls";
   filename = StringJoin[string1, string2, "_", string3, string4];

   Export[filename,
    {"PCoA" → k, "FCC" → FCCmatrix, "EnzymeIDs" → newvmaxes[[2 ;; 12, 1]]}];
   z = z + dz;]
 ], ProgressIndicator[z, {zstart, zend}]];
```

```
ParallelTable[{Vars[[i]][t], (Vars[[i]][1000] /. tsol)[[1]]}, {i, 1, Length[Vars]}];
ss1 = FindRoot[
   Table[Odes[[i, 2]] == 0, {i, 1, Length[Odes]}] /. RateEqs /. CoAMATX /. Parm /. Parm2,
   %];
fluxes1 = RateEqs /. CoAMATX /. Parm /. Parm2 /. ss1;
Metabolites =
  TableForm[ParallelTable[{ss1[[i]][[1]], ss1[[i]][[2]]}, {i, 1, Length[ss1]}]];
Flux = TableForm[ParallelTable[{fluxes1[[i]][[1]], fluxes1[[i]][[2]]},
    {i, 1, Length[fluxes1]}]];
```

Import data

```
newvmaxes = Import["C:\\vmaxestable_estimatedCPT1.csv", "Data"];
```

Run function FCCMuscle

```
FCCMuscle[2, 1, Length[newvmaxes[[1]]]]
```

FCC results exported within each loop

Steady-state values

```
ParallelTable[{Vars[[i]][t], (Vars[[i]][1000] /. tsol)[[1]]}, {i, 1, Length[Vars]}];
```

```
ss1 = FindRoot[
  Table[Odes[[i, 2]] == 0, {i, 1, Length[Odes]}] /. RateEqs /. CoAMATX /. Parm /. Parm2,
  %]
fluxes1 = RateEqs /. CoAMATX /. Parm /. Parm2 /. ss1
Metabolites =
 TableForm[ParallelTable[{ss1[[i]][[1]], ss1[[i]][[2]]}, {i, 1, Length[ss1]}]]
Flux = TableForm[ParallelTable[
   {fluxes1[[i]][[1]], fluxes1[[i]][[2]]}, {i, 1, Length[fluxes1]}]]
```

```
CoAMAT1 = CoAMATt - C16AcylCoAMAT[t] - C16EnoylCoAMAT[t] - C16HydroxyacylCoAMAT[t] -
      C16KetoacylCoAMAT[t] - C14AcylCoAMAT[t] - C14EnoylCoAMAT[t] -
      C14HydroxyacylCoAMAT[t] - C14KetoacylCoAMAT[t] - C12AcylCoAMAT[t] -
      C12EnoylCoAMAT[t] - C12HydroxyacylCoAMAT[t] - C12KetoacylCoAMAT[t] -
      C10AcylCoAMAT[t] - C10EnoylCoAMAT[t] - C10HydroxyacylCoAMAT[t] -
      C10KetoacylCoAMAT[t] - C8AcylCoAMAT[t] - C8EnoylCoAMAT[t] -
      C8HydroxyacylCoAMAT[t] - C8KetoacylCoAMAT[t] - C6AcylCoAMAT[t] -
      C6EnoylCoAMAT[t] - C6HydroxyacylCoAMAT[t] - C6KetoacylCoAMAT[t] -
      C4AcylCoAMAT[t] - C4EnoylCoAMAT[t] - C4HydroxyacylCoAMAT[t] -
      C4AcetoacylCoAMAT[t] - AcetylCoAMAT[t] /. RateEqs /. Parm /. Parm2 /. ss1
```

```
NumberForm[CoAMAT1, 16];
```

```
steadyStateMuscle[zstart_, dz_, zend_] := Monitor[Module[{vector2},
    k = {0.1, 0.5, 1.0, 1.5, 2.0, 2.5, 3.0, 3.5,
      4.0, 4.5, 5.0, 5.5, 6.0, 6.5, 7.0, 7.5, 8.0, 8.5, 9.0, 9.5, 10.0};
    Fluxesmatrix = {};
```

```
a = newvmaxes[[1]];
animalIDs = Delete[a, 1];
For[z = zstart, z ≤ zend,
 Fluxesvector = {};
 vectorvmaxes = {0};
 For[i = 2, i ≤ 12, i++,
  AppendTo[vectorvmaxes, newvmaxes[[i, z]]];
    ];
 vector2 = vectorvmaxes;
 Parm2replaced =
  {Vmckat → vector2[[2]], Vmcad → vector2[[3]], Vscad → vector2[[4]],
   Vvlcad → vector2[[5]], Vcpt1 → vector2[[6]], Vcpt2 → vector2[[7]],
   Vcrot → vector2[[8]], Vmschad → vector2[[9]], Vmtp → vector2[[10]],
   Vfcact → vector2[[11]], Vrcact → vector2[[11]], Vlcad → vector2[[12]]};

 tsol0 = ParallelTable[NDSolve[Join[Odes /. RateEqs /. CoAMATX /. ReplacePart[Parm,
        Position[Parm, C16AcylCoACYT][[1, 1]] → C16AcylCoACYT → k[[j]]] /.
      Parm2replaced, ReplacePart[InitialConditions, {ii_, 2} :> ss1[[ ii, 2]]]],
    Vars, {t, 0, 1000}], {j, 1, Length[k]}];
 ssp0 = Table[FindRoot[Table[Odes[[i, 2]] == 0, {i, 1, Length[Odes]}] /. RateEqs /.
       CoAMATX /. ReplacePart[Parm, Position[Parm, C16AcylCoACYT][[1, 1]] →
        C16AcylCoACYT → k[[j]]] /. Parm2replaced,
    ParallelTable[{Vars[[i]][t], (Vars[[i]][1000] /. tsol0[[j]])[[1]]},
     {i, 1, Length[Vars]}]], {j, 1, Length[k]}];

 Jk = ParallelTable[RateEqs[[All, 2]] /. CoAMATX /. ReplacePart[Parm,
       Position[Parm, C16AcylCoACYT][[1, 1]] → C16AcylCoACYT → k[[j]]] /.
     Parm2replaced /. ssp0[[j]], {j, 1, Length[k]}];
 J0 = TableForm[ParallelTable[Jk[[j, m]], {j, 1, Length[k]},
    {m, 1, Length[RateEqs]}]];

 Jknames = fluxes1[[All, 1]];
 Metk = ParallelTable[ssp0[[j, All, 2]], {j, 1, Length[k]}];
 Met0 = TableForm[Metk];
 Metknames = ss1[[All, 1]];
 Jkmat = ConstantArray[0, {Length[Jk[[1]]], Length [Jk[[All, 1]]] + 1}];
For[i = 1, i ≤ Length[Jk[[1]]], i++,
  Jkmat[[i, 1]] = Jknames[[i]];
  For[j = 1, j ≤ Length[Jk], j++,
   Jkmat[[i, j + 1]] = Jk[[j, i]]
  ]
 ];
Metkmat = ConstantArray[0, {Length[Metk[[1]]], Length [Metk[[All, 1]]] + 1}];
 For[i = 1, i ≤ Length[Metk[[1]]], i++,
  Metkmat[[i, 1]] = Metknames[[i]];
  For[j = 1, j ≤ Length[Metk], j++,
   Metkmat[[i, j + 1]] = Metk[[j, i]]
  ]
 ];
 Fluxesvector = Jkmat[[1]];
```

```
      AppendTo[Fluxesmatrix, Fluxesvector];
      z = z + dz;]
   ], ProgressIndicator[z, {zstart, zend}]];
```

Import data

```
newvmaxes = Import["C:\\vmaxestable_estimatedCPT1.csv", "Data"];
```

Run function timeCourseMuscle

```
steadyStateMuscle[2, 1, Length[newvmaxes[[1]]]]
```

Export results

```
Export["C:\\SteadyStateFluxes.xls",
  {"PCoA" → k, "AllFluxes" → Fluxesmatrix, "Animal IDS" → animalIDs}];
```
